# Quenched Lewis Acidity: Studies on the Medium Dependent Fluorescence of Zinc(II) Complexes

**DOI:** 10.1002/chem.202102086

**Published:** 2021-10-04

**Authors:** Hannah Kurz, Gerald Hörner, Oskar Weser, Giovanni Li Manni, Birgit Weber

**Affiliations:** ^1^ Inorganic Chemistry IV University of Bayreuth Universitätsstraße 30 95447 Bayreuth Germany; ^2^ Max Planck Institute for Solid State Research Heisenbergstraße 1 70569 Stuttgart Germany

**Keywords:** CASSCF, dimerization, fluorescence, Schiff base, zinc

## Abstract

Three new zinc(II) coordination units **[Zn(1–3)]** based on planar‐directing tetradentate Schiff base‐like ligands **H_2_(1**–**3)** were synthesized. Their solid‐state structures were investigated by single crystal X‐ray diffraction, showing the tendency to overcome the square‐planar coordination sphere by axial ligation. Affinity in solution towards axial ligation has been tested by extended spectroscopic studies, both in the absorption and emission mode. The electronic spectrum of the pyridine complex **[Zn(1)(py)]** has been characterized by MC‐PDFT to validate the results of extended TD‐DFT studies. Green emission of non‐emissive solutions of **[Zn(1–3)]** in chloroform could be switched on in the presence of potent Lewis‐bases. While interpretation in terms of an equilibrium of stacked/non‐fluorescent and destacked/fluorescent species is in line with precedents from literature, the sensitivity of **[Zn(1–3)]** was greatly reduced. Results of a computation‐based structure search allow to trace the hidden Lewis acidity of **[Zn(1–3)]** to a new stacking motif, resulting in a strongly enhanced stability of the dimers.

## Graphical abstract

**Three new zinc(II) coordination units** based on Schiff base‐like ligands equipped with nitrile groups were synthesized. In non‐coordinating solvents, such as chloroform, a close‐to‐zero emission was observed; this increased drastically upon addition of the Lewis base pyridine (Φ=15 %). Interestingly, DFT calculations indicate that the intrinsically high Lewis‐acidity of the zinc(II) metal center is hidden through formation of highly stable dimers.

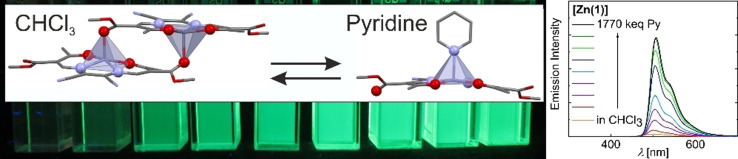

## Introduction

Switchable materials are of great interest for applications as molecular sensor materials.^[1]^ These materials need to combine a high sensitivity toward external physical or chemical stimuli with an easily detectable change of discrete properties such as photoluminescence. For this purpose, metal complexes with photoluminescent ligands are an interesting class of materials, as they can combine an easily tunable photoluminescence based on the ligand with a switching behavior based on the metal center. Various switching processes are possible that depend on the metal center and its electronic configuration. One possibility is the switching based on externally induced spin transitions. Two well‐known phenomena are *spin crossover*, which is mostly investigated for iron(II) complexes,^[2]^ and *coordination‐induced spin state switching* that can be observed with nickel(II) complexes.^[3]^ In recent years, many workgroups focused on combining one of these two switching mechanisms with a photoluminescent ligand to obtain emissive sensor materials.^[4]^ However, electronic coupling of metal and ligand often results in predominant non‐radiative excited‐state decay into low‐lying *d*‐*d* states, independent of the spin state.^[5]^

Due to these drawbacks, recently much emphasis was put on metal centers with closed *d* ^0^ or *d* ^10^ shells such as Zr(IV), Cu(I), or Zn(II). As the *d* orbitals of the metal center are not directly electronically involved in the photophysical processes, the emission is not quenched and a wide range of photoluminescent complexes has been reported.[^5b^, ^6^] Especially, zinc(II) complexes are interesting for the application as molecular sensor materials, as their emission is often medium dependent. In the last decade many zinc(II) complexes have been reported that show aggregation‐induced emission enhancement (AIEE).^[7]^

Interestingly, the opposite case – an emission quenching due to stacking – has been observed as well with zinc(II) complexes. Many studies focus on zinc(II) complexes based on a Schiff base tetradentate ligand equipped with nitrile substituents.^[8]^ Coordination of additional ligands resulted in a strong increase of the emission intensity. Experimental and computational studies suggested that the zinc(II) complexes stack into dimers or oligomers in solvents that do not support axial coordination.[^8d^, ^8f^] The high emission intensity increase in coordinating solvents was assigned to de‐stacking of the zinc(II) complexes.[^8b^, ^8d^] This effect was used previously for bioimaging and biosensing in living cells.[^8c^, ^8g^, ^9^] Furthermore, derivatives of this zinc(II) complex type were investigated for their mechanochromic luminescence behavior, and as emitters for optical temperature sensing via thermally activated delayed fluorescence (TADF).^[10]^

**Scheme 1:**
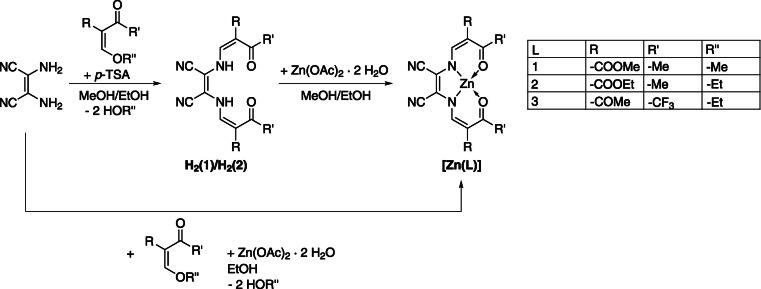
Synthetic pathway toward the reported zinc(II) coordination units **[Zn(1–3)]**.

Herein, we present a concerted experiment‐theory approach to investigate the influence of the molecular structure on the fluorescence of zinc(II) complexes. A family of neutral complexes of Schiff base‐like ligands with appended nitrile groups **[Zn(L)X]** (for the nature of **L**, see Scheme 1; **X**=H_2_O, EtOH, THF, py) was synthesized. Please note that the simplified notation **[Zn(1–3)]** is used whenever the nature of the axial ligand cannot be specified. In agreement with the established stacking/destacking hypothesis put forward by Di Bella and others for analogue Schiff base derived complexes,[^8d^, ^8f^] turn‐on emission behavior is observed in spectroscopic titrations with Lewis‐bases, but requires much higher base loads. The seemingly weakened Lewis‐base affinity of **[Zn(L)X]** was found to be due to quenched Lewis‐acidity based on the formation of highly stable dimers in solvents that do not support axial coordination. Results from (time‐dependent) density functional theory are benchmarked through complete active space self‐consistent field methods. CASSCF, followed by the multi‐configurational pair‐density functional theory correction (MC‐PDFT) accurately captures correlation effects for the relevant states involved in the diagnostic ILCT process.

## Results and Discussion

### Complex syntheses and characterization

The zinc(II) coordination units **[Zn(1)]** and **[Zn(2)]** were synthesized in two steps as shown in Scheme 1; **[Zn(3)]** was synthesized in a one‐pot reaction as the synthesis of the free ligand **H_2_(3)** failed. In the case of **H_2_(1)** and **H_2_(2)** the free Schiff base‐like ligands were obtained directly through reaction of diaminomaleonitrile with the respective keto‐enol ether in the presence of *p*‐toluenesulfonic acid (*p*‐TSA); **H_2_(2)** has been reported previously.^[11]^ **H_2_(1)** was received from MeOH as an orange solid in 44 % yield. The identity and purity of the ligands was confirmed by ^1^H NMR spectroscopy, mass spectrometry, and elemental analysis. It is noted that resonances of the NH hydrogen atom could not be observed spectroscopically, neither in IR nor ^1^H NMR. This finding indicates fast exchange in solution and strong involvement of the NH hydrogen atoms in a hydrogen bond network in the solid.

From the ligands the respective zinc(II) complexes were obtained through conversion with stoichiometric amounts of Zn(OAc)_2_ ⋅ 2 H_2_O in alcohol media. Thereby, acetate provides the required base equivalents. In this manner **{[Zn(1)](H_2_O)(MeOH)}** and **{[Zn(2)]_2_(H_2_O)_3_}** were obtained as a red crystalline (70 %) and an orange solid (47 %), respectively. **{[Zn(3)](H_2_O)(EtOH)}** was received as an orange solid (66 %) in a zinc‐templated three‐component reaction in EtOH (diaminomaleonitrile, keto‐enol ether, Zn(OAc)_2_ ⋅ 2 H_2_O; 1 : 2.2 : 1.3). Sample homogeneity and purity was established by ^1^H NMR spectroscopy and mass spectrometry. Elemental analysis indicated the presence of stoichiometrically defined amounts of solvent molecules in the samples as indicated by the above empirical formulae. X‐ray diffraction of single‐crystalline samples indeed identified the complexes to be five (N_2_O_3_) and six‐coordinate (N_2_O_4_) in the solid, besides additional solvent molecules in the lattice.

### X‐ray diffraction analysis

**Figure 1:**
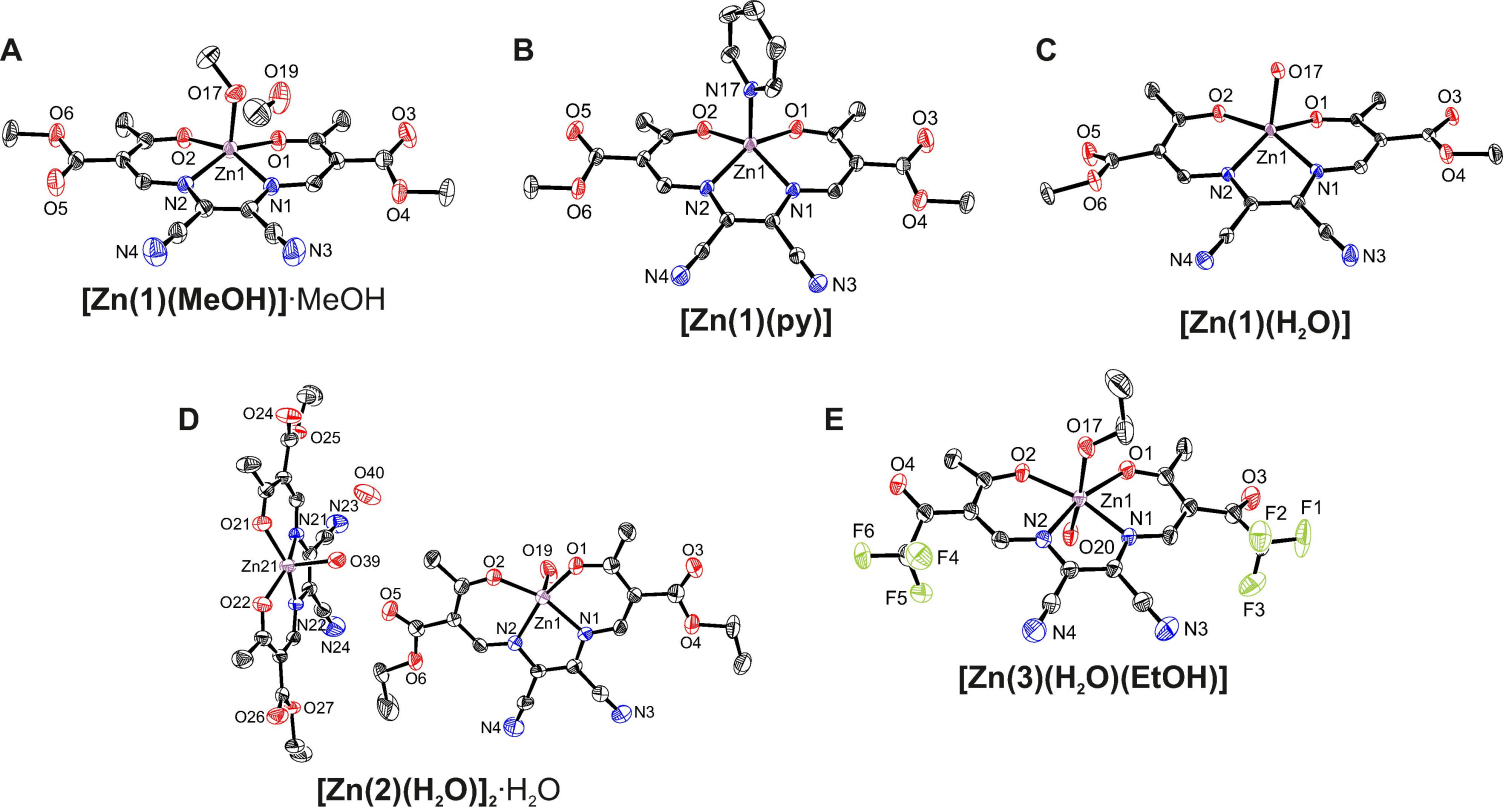
Structures of the asymmetric units of **[Zn(1)(MeOH)]⋅MeOH** (A), **[Zn(1)(py)]** (B), **[Zn(1)(H**_***2***_ **O)]** (C), **[Zn(2)(H_2_O)]_2_⋅H_2_O** (D), and **[Zn(3)(H_2_O)(EtOH)]** (E). Hydrogen atoms are omitted for clarity. Ellipsoids are shown at 50 % probability level.

Molecular structures and packing pattern were addressed by single‐crystal X‐ray diffraction. The crystallographic data of all crystal structures are summarized in Table S1/S2 in the Supporting Information. Orange block‐like crystals of **[Zn(1)(MeOH)]⋅MeOH** were obtained directly from the mother liquor. The material crystallises in the orthorhombic space group *Pbca*. The asymmetric unit consists of the five‐coordinate zinc(II) complex and one solvent MeOH molecule as shown in Figure 1A (see Figure S1 for a fully labelled representation of the asymmetric unit). The zinc(II) center is enclosed in a close‐to‐ideal tetragonal pyramidal N_2_O_3_ coordination sphere (*S*_p_(SqPy)=1.18),^[12]^ wherein an axial methanol ligand adds to the N_2_O_2_ chelate of the Schiff base‐like ligand. Selected bond lengths are given in Table 1. The average bond lengths within the chelate cycle are 1.99 Å (Zn1−O_eq_), 2.06 Å (Zn1−N_eq_), and 2.01 Å (Zn1−O_ax_). The *cis*‐angles including the zinc(II) metal center are in the range of 97–104°, which indicates an almost ideal square‐pyramidal coordination sphere.

**Table 1:** Selected bond lengths [Å] and bond angles [°] of **[Zn(1)(MeOH)]⋅MeOH**, **[Zn(1)(py)]**, **[Zn(1)(H**_***2***_ **O)]**, **[Zn(2)(H_2_O)]_2_⋅H_2_O**, and **[Zn(3)(H_2_O)(EtOH)]**.

Compound	Bond	Bond length	Bonds	Bond angle
**[Zn(1)(MeOH)] ⋅ MeOH**	Zn1‐O_eq_	1.9925(15) 1.9909(15)	O_eq_‐Zn1‐O_eq_	99.21(6)
	Zn1‐N_eq_	2.0562(18) 2.0572(18)	N_eq_‐Zn‐O_ax_	97.78(7) 103.77(7)
	Zn1‐O_ax_	2.0091(19)	O_eq_‐Zn‐O_ax_	97.15(7) 103.34(7)
**[Zn(1)(py)]**	Zn1‐O_eq_	2.0060(18) 2.0074(16)	O_eq_‐Zn1‐O_eq_	93.86(7)
	Zn1‐N_eq_	2.0708(18) 2.0576(19)	N_eq_‐Zn1‐N_ax_	110.08(7) 108.28(8)
	Zn1‐N_ax_	2.0480(18)	O_eq_‐Zn1‐N_ax_	99.31(7) 101.00(7)
**[Zn(1)(H_2_O)]**	Zn1‐O_eq_	2.0117(11) 1.9866(11)	O_eq_‐Zn1‐O_eq_	98.92(4)
	Zn1‐N_eq_	2.0577(12) 2.0624(12)	N_eq_‐Zn‐O_ax_	102.68(5) 112.45(5)
	Zn1‐O_ax_	2.0555(12)	O_eq_‐Zn‐O_ax_	96.41(5) 97.30(5)
**[Zn(2)(H_2_O)]_2_⋅H_2_O**	Zn1‐O_eq_	2.000(3) 2.003(3)	O_eq_‐Zn1‐O_eq_	99.36(12)
	Zn1‐N_eq_	2.062(3) 2.055(3)	N_eq_‐Zn‐O_ax_	102.30(15) 103.35(16)
	Zn1‐O_ax_	1.991(5)	O_eq_‐Zn‐O_ax_	99.19(15) 100.36(15)
**[Zn(3)(H_2_O) (EtOH)]**	Zn1‐O_eq_	2.057(3) 2.043(3)	O_eq_‐Zn1‐O_eq_ O_ax_‐Zn1‐O_ax_	111.90(12) 166.53(16)
	Zn1‐N_eq_	2.090(4) 2.098(4)	O_eq_‐Zn‐O_ax,EtOH_	87.12(13) 86.91(14)
	Zn1‐O_ax_	2.129(4) 2.108(4)	O_eq_‐Zn‐O_ax,H2O_	85.91(13) 85.02(14)

Yellow plate‐like crystals of **[Zn(1)(py)]** were obtained by adding deuterated pyridine to a solution of **{[Zn(1)](H_2_O)(MeOH)}** in deuterated acetonitrile. In the triclinic space group *P* $\bar{1}$ , the asymmetric unit consists of one five‐coordinate zinc(II) complex as shown in Figure 1B (see Figure S2 for a fully labelled representation of the asymmetric unit). The zinc(II) center is enclosed in a N_3_O_2_ coordination sphere due to an axially coordinating pyridine molecule. The bond lengths (2.01 Å (Zn1−O_eq_), 2.06 Å (Zn1−N_eq_), 1.99 Å (Zn1−N_ax_) are very similar to the ones of **[Zn(1)(MeOH)]⋅MeOH**. However, the square‐pyramidal coordination sphere features a slightly higher distortion with bond angles of 94–110° (*S*_p_(SqPy)=2.07).^[12]^ A very similar asymmetric unit with five‐coordinate zinc(II) complexes could be obtained by crystallization from wet acetonitrile. Thereby, orange rhombohedral‐like crystals of **[Zn(1)(H_2_O)]** were obtained that crystallize in the triclinic space group *P* $\bar{1}$ . The asymmetric unit consists of a five‐coordinate zinc(II), where one water molecule acts as the axial ligand leading to a N_2_O_3_ coordination sphere (see Figure 1C and Figure S3 in the Supporting Information for a fully labelled representation of the asymmetric unit). The average bond lengths are with 2.00 Å (Zn1−O_eq_), 2.06 Å (Zn1−N_eq_), and 2.06 Å (Zn1−O_ax_) very similar to the ones of **[Zn(1)(MeOH)]⋅MeOH**. However, similar to **[Zn(1)(py)]**, the square‐pyramidal coordination sphere shows a slightly higher degree of distortion (*S*_p_(SqPy)=1.97).^[12]^

Similar to **[Zn(1)(MeOH)]⋅MeOH**, the zinc(II) center in **[Zn(2)(H_2_O)]_2_⋅H_2_O** is enclosed in a N_2_O_3_ coordination sphere where water ligates axially. Orange block‐like single crystals of **[Zn(2)(H_2_O)]_2_⋅H_2_O** obtained from the mother liquor were analysed in the triclinic space group *P* $\bar{1}$ . The asymmetric unit consists of two five‐coordinate zinc(II) complexes and one water solvent molecule (see Figure 1D and Figure S4 for a fully labelled representation of the asymmetric unit). The bond lengths and angles (2.00 Å (Zn1−O_eq_), 2.06 Å (Zn1−N_eq_), 1.99 Å (Zn1−O_ax_), 99–104°) of the largely undistorted square‐pyramidal coordination sphere (*S*_p_(SqPy)=1.28/1.36)^[12]^ closely match the data of **[Zn(1)(MeOH)]⋅MeOH**. Interestingly, the metrics of all these complexes closely mimic structures which have been reported of five‐coordinate zinc(II) complexes deriving from *salmant* (2,2‘‐[(1,2‐Dicyanoethene‐1,2‐diyl)bis‐(nitrilomethanylylidyne)]‐diphenol)^[13]^ and *salophen* (2,2’‐[1,2‐phenylenebis(nitrilomethyl‐idyne)]diphenol) ligands.^[14]^

Finally, red block‐like crystals of **[Zn(3)(H_2_O)(EtOH)]** were obtained from the mother liquor. Different from **[Zn(1/2)]**, the fragment **[Zn(3)]** exhibits electron withdrawing CF_3_ substituents. This increase in Lewis's acidity of the zinc(II) metal center is directly reflected in a coordination of two axial ligands leading to a six‐coordinate zinc(II) complex. In the monoclinic space group *P*2_1_/*c*, the asymmetric unit consists of one mononuclear six‐coordinate zinc(II) complex, where the axial positions are occupied by one water and one EtOH ligand (see Figure 1E and Figure S5 in the Supporting Information for a fully labelled representation of the asymmetric unit). This coordination results in an N_2_O_4_ coordination sphere. The average bond lengths (2.05 Å (Zn1−O_eq_), 2.09 Å (Zn1−N_eq_), 2.12 Å (Zn1−O_ax_)) are significantly longer than in the five‐coordinate congeners **[Zn(1)(MeOH)]** and **[Zn(2)(H_2_O)]**. The O_ax_‐Zn1‐O_ax_ *trans*‐angle of 166.53(16) and *cis*‐angles of 85–87° indicate a distorted octahedral coordination sphere (*S*_p_(O_h_)=1.83).^[12]^

**Figure 2:**
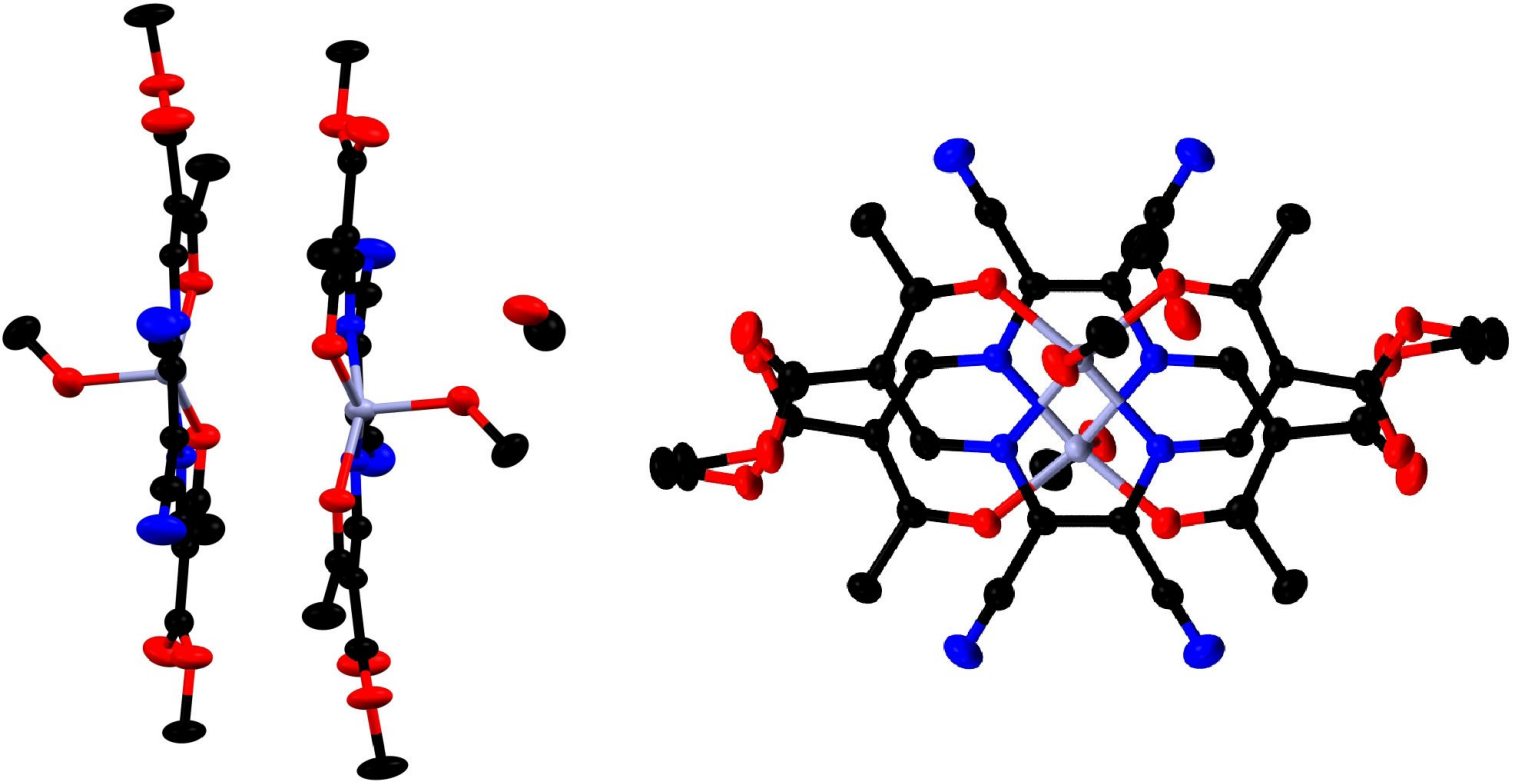
Side view and top view of the stacked dimers in **[Zn(1)(MeOH)]⋅MeOH**. Hydrogen bonds are omitted for clarity. Ellipsoids are shown at 50 % probability level.

In the crystal, *π*‐*π* interactions between the chelate Zn1−N1−C4−C5−N2 of neighboring complexes and towards the zinc(II) metal center result into stacked dimers, in **[Zn(1)(MeOH)]**, **[Zn(1)(H_2_O)]**, **[Zn(2)(H_2_O)]_2_⋅H_2_O** (see Figure 2 and Figure S6 and S9 in the Supporting Information; Table S3 in the Supporting Information for distances and angles of the *π*‐*π* and M‐*π* interactions). These stacked dimers interact with neighboring molecules through a hydrogen bond network (see Figure S7, S8, and S10 in the Supporting Information; Table S4 in the Supporting Information for hydrogen bond and angles). The supramolecular packing in **[Zn(1)py]** clearly differs from the above cases, as no stacked dimers are observed due to the coordination of pyridine. The packing is similar to the above described cases dominated by non‐classical hydrogen bonds forming columns (see Figure S11 in the Supporting Information; Table S3/4 in the Supporting Information). In the case of **[Zn(3)(H_2_O)(EtOH)]**, where the zinc(II) metal centers are six‐coordinated, the packing is likewise dominated by hydrogen bonds resulting in a 3D hydrogen bond network (see Figure S12 in the Supporting Information; Table S4 in the Supporting Information for hydrogen bond and angles).

Powder X‐ray diffraction patterns of the zinc(II) complexes **[Zn(2/3)]** at RT are matched by the calculated patterns of **[Zn(2/3)]** based on the single‐crystals data (Figure S13 in the Supporting Information), indicating conserved formulations of bulk and crystalline samples. In contrast, the calculated pattern of **[Zn(1)]** clearly differs from the experimental data, as could be expected from the different constitution of bulk powder and single crystal.

### Optical properties of the zinc(II) complexes

**Steady‐state absorption and emission**: Due to the closed shell character and a large net nuclear charge, zinc(II) centers are usually not directly involved in optical processes such as absorption or emission. In particular, radiative transitions with predominating MLCT character that are the basis of the rich photophysics and photochemistry of copper(I) are prohibited in isoelectronic zinc complexes. Instead, optical excitation and relaxation are largely dominated by the ligand. Nevertheless, coordination of zinc(II) by the ligands **H_2_(1)** and **H_2_(2)** after deprotonation clearly affects the optical properties in solution as they are strongly red‐shifted going from a yellow to an orange solution.

**Figure 3:**
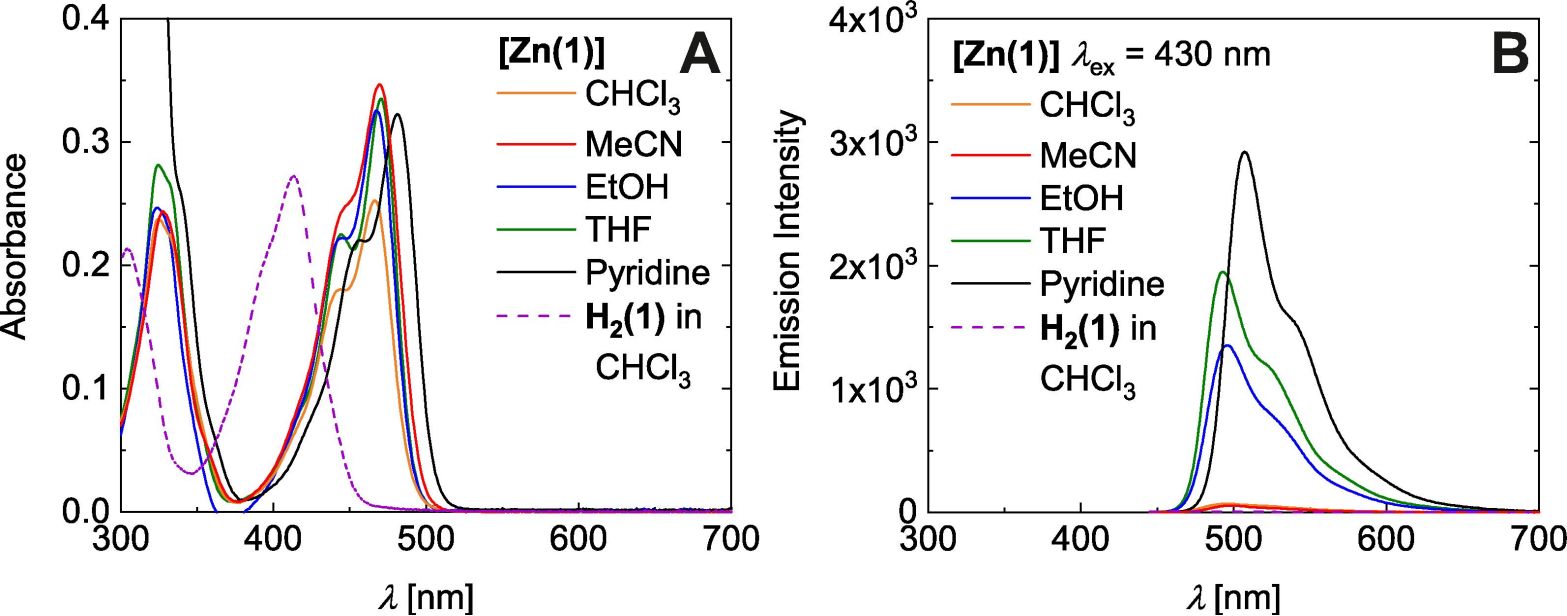
Absorbance (A) and emission (B) spectra of **[Zn(1)]** in CHCl_3_, MeCN, EtOH, THF, and pyridine (7×10^−6^ M).

Accordingly, the optical spectra are dominated by intense absorption bands centered at 413 nm (ϵ_M_=3.9/3.6 ⋅ 10^4^ M^−1^cm^−1^) and 467 nm (ϵ_M_=3.6 ⋅ 10^4^ M^−1^cm^−1^) for dilute solutions in chloroform (*c*=7 ⋅ 10^−6^ M) of the ligands **H_2_(1/2)** and the zinc complexes **[Zn(1/2)]**, respectively. The leading absorption in **[Zn(3)]** bearing CF_3_ substituents is located at 451 nm (see Figure 3A and Figure S14/15 in the Supporting Information). Exemplarily, this distinct red shift upon coordination by Δν=2800 cm^−1^ is shown for ligand **H_2_(1)** and **[Zn(1)]** in Figure 3A; similar observations hold for **H_2_(2)**/**[Zn(2)]** (Figure S14B and S15 A in the Supporting Information). The strong UV band in the ligand spectrum at 305 nm is likewise shifted to smaller energy by Δν=2100 cm^−1^. Spectral shifts of ligand‐centered bands upon coordination are not unique. For a topologically related couple deriving from salicylic aldehyde, a coordination‐dependent red shift has been recorded from 374 nm (in acetonitrile) for the ligand^[15]^ to 560 nm for the zinc complex (in DMSO);^[16]^ that is, excitation energy shifts by as much as Δν=8800 cm^−1^.

**[Zn(1)]** shows weak fluorescence in CHCl_3_, that is in a non‐coordinating environment (λ_Em_=495 nm; Φ_F_≈0.004; all reported quantum yields are relative to **[Zn(1)]** in pyridine by the comparison of the absorbance corrected integrated emission intensity). Similar observations hold for **[Zn(2)]** and **[Zn(3)]**, with somewhat smaller quantum yield Φ_F_ of the latter. By contrast the free ligands are essentially non‐emissive under the same conditions (Φ_Em_≈10^−4^); it is noted that the salicylic analogue of ligand **H_2_(1)** supports fluorescence with Φ_Em_=0.017.^[15]^ The spectra of all zinc(II) complexes indicate partly resolved vibrational structure, both in absorption and in emission. An energy spacing of the vibrational progression by Δ_vib_ *E*≈1100 cm^−1^ points to a leading role of chelate skeletal modes. This assignment is supported by analysis of DFT‐derived harmonic frequencies (see Figure S16 in the Supporting Information and animated gif).

Quite generally zinc complexes of planar‐directing N_2_O_2_ ligands show significant solvatochromism in both, absorption and emission.[^8b^, ^8e^] Different from these literature precedents, a solvent scan shows that the absorption spectra of unit **[Zn(1)]** are largely indifferent to solvent variation with respect to energy and band shape (Figure 3A). It is only in neat pyridine that absorption is affected. Notwithstanding the almost invariant absorption spectra, however, emission properties of **[Zn(1)]** vary substantially with solvent (see Figure 3B). While emission is very weak in chloroform and acetonitrile (Φ_Em_(CHCl_3_)=Φ_Em_(MeCN)≈0.004), it is greatly enhanced in EtOH and THF (Φ_Em_(EtOH)≈0.07 and Φ_Em_(THF)≈0.10) and reaches its maximum in neat pyridine (Φ_Em_(Py)≈0.15). As a matter of fact, fluorescence of **[Zn(1)]** which is silent in acetonitrile, can be increased successively through addition of water (see Figure S17 in the Supporting Information). Increasing the water content up to 50 vol‐% results in a quantum yield increase up to Φ_Em_(MeCN/H_2_O 1/1)≈0.013. Coordination of Lewis‐basic solvent molecules to the Lewis‐acidic zinc center must be considered as the underlying molecular factor that switches on fluorescence in **[Zn(1)]**. This notion is supported by the continuous increase of Φ_Em_ with the donor number of the solvent (see Figure S18 in the Supporting Information).

**Figure 4:**
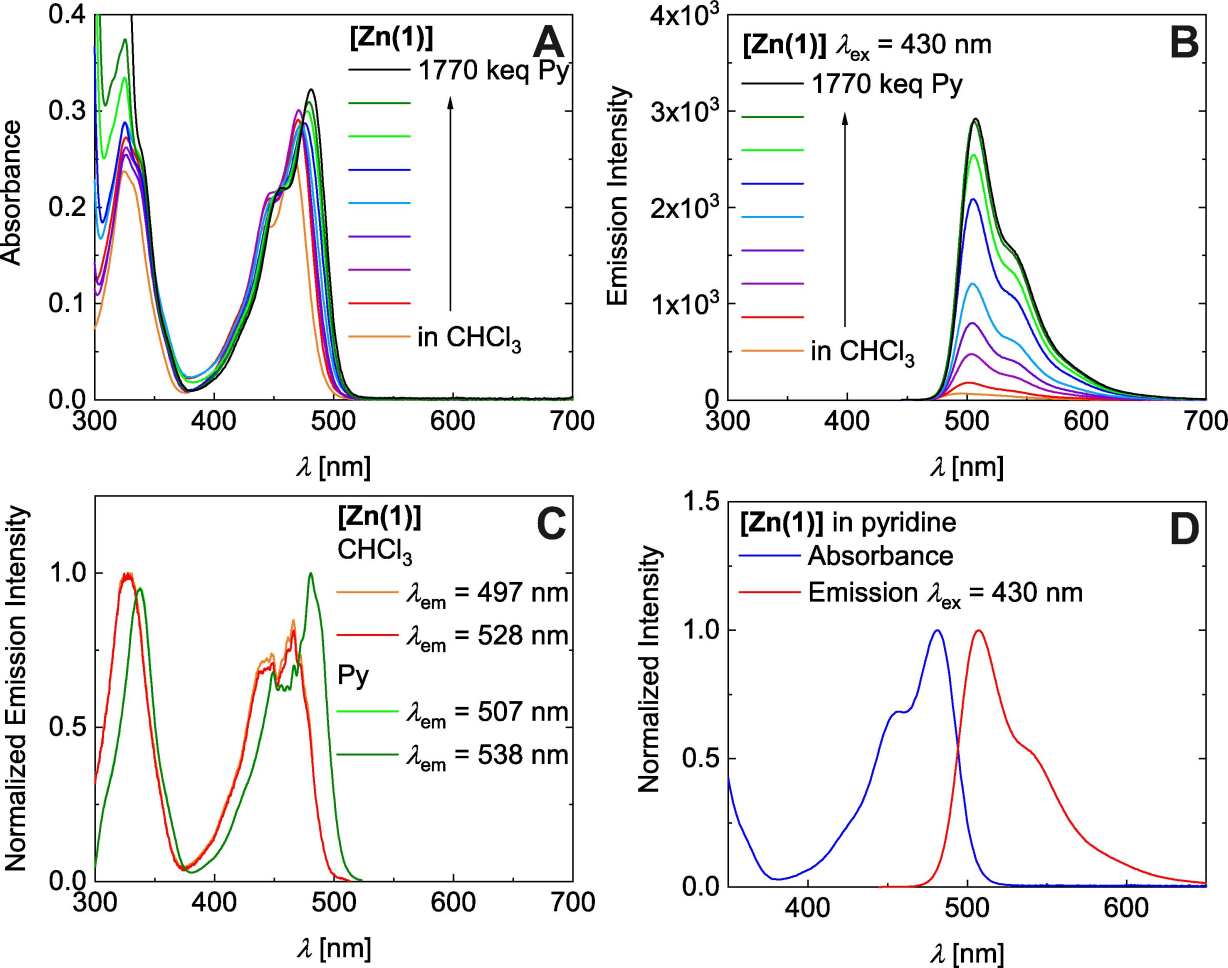
Chloroform/pyridine series: Absorption spectra of **[Zn(1)]** (**A**). Emission spectra of **[Zn(1)]** (*λ*_exc_=430 nm; **B**). Fluorescence excitation spectra of **[Zn(1)]** (**C**). Absorbance and emission spectra of **[Zn(1)]** in pyridine (**D**).

Shown in Figure 4A is a titration of equally concentrated solutions of **[Zn(1)]** in CHCl_3_ with pyridine, which yields a continuous red‐shift by up to 15 nm (see Figure S15A/B in the Supporting Information for titrations of **[Zn(2)]** and **[Zn(3)]**, see Figure S19 for photographs), coupled to an intensity variation between 3.6×10^4^ cm^−1^ M^−1^<ϵ_M_<4.6×10^4^ cm^−1^ M^−1^. (see Figure 4A). Fluorometric titration with pyridine shows a continuously enhanced emission for **[Zn(1)]** and **[Zn(2)]** which saturates only at the highest pyridine loads, which equals neat pyridine (see Figure 4B and Figures S15C in the Supporting Information). Fluorescence excitation spectra are indifferent to the observation wavelength and qualitatively map the absorption spectrum across the entire UV‐Vis range (red in Figure 4C, Figure S15E/F in the Supporting Information). Thereby, the pyridine‐dependent red shift in absorption is compensated by a parallel red shift in emission so that the Stokes‐shift of Δ_Stokes_ *E*≈1100 cm^−1^ is preserved. Likewise, the coincidence of absorption and fluorescence excitation spectra (deviation at λ<330 nm is due to uncompensated absorption by excess pyridine) and the vibrational structure of the diagnostic bands are maintained nearly constant (see Figure 4D and Figure S15E in the Supporting Information).

**DFT and wave‐function theory analysis of the electronic structure**: The nature of the leading optical transition of the zinc complexes has been addressed by means of (time‐dependent) density‐functional theory and wave function‐based approaches. Complete active space self‐consistent field, CASSCF, calculations were performed for the **[Zn(1)py]** complex, with an active space comprising the entire system of π‐orbitals and their electrons, CAS(18,16). This represents the main set of orbitals responsible for the luminescence process. The lowest two singlet spin states (S_0_ and S_1_ states) and the lowest triplet spin state (T_1_) wave functions have been optimized at the CASSCF level of theory, followed by a multiconfiguration pair‐density functional theory approach (MC‐PDFT) for treating dynamic correlation effects outside the chosen active space.^[17]^ The singlet state calculations were performed at the DFT optimized geometry (pertinent metrics in Tables S5/6, Supporting Information). Triplet spin state calculations were performed both at the optimized triplet geometry (*adiabatic excitation*) and at the singlet geometry (*vertical excitation*).

According to theory, the photophysics of **[Zn(1)X]** is invariably centered at the chelate ligand. Variation of the axial ligand, X, does not considerably affect the transition frequency, neither in experiment nor theory (see Table 2). MC‐PDFT calculations on **[Zn(1)py]** predict the S_0_→S_1_ transition at 512 nm, while DFT predicts the same transition at 440 nm, both in fair agreement with the experimental value (481 nm) (see Table 2). TD‐DFT tends to systematically overestimate the energy of the leading Vis transition in the entire ligand‐substitution series **[Zn(1)X]** (X: H_2_O; ROH; pyridine; average deviation over five‐coordinate complexes, Δν=1700 cm^−1^), while MC‐PDFT underestimates the transition energy for **[Zn(1)py]**. Nonetheless, our theoretical predictions match the overall pattern and intensities in the experimental data.

**Table 2:** Medium dependence of the S_0_→S_1_ transition of **[Zn(1)**] from experiment and theoretical modelling (λ_max_ [nm]; ϵ_max_ [10^4^ M^−1^ cm^−1^]).

	Experimental		Theoretical^[a]^		
Medium	λ_max_	ϵ_max_	λ_max_	f_osc_	Nature
chloroform	466	3.4	414 (487)^[b]^	0.784^[b]^	H−2→L (34 %); H→L (59 %)
			433^[c]^	1.244^[c]^	H−2→L+1 (44 %); H→L (39 %)
MeCN	469	5.0	430^[d]^	0.782^[d]^	H→L (92 %)
MeCN/water (1 : 1)	467	4.4	426^[d]^	0.791^[d]^	H→L (92 %)
EtOH/MeOH	467	4.6	427^[d]^	0.746^[d]^	H→L (90 %)
THF	471	4.8	430^[d]^	0.726^[d]^	H→L (90 %)
pyridine	481	4.6	440 (512)^[d]^	0.673^[d]^	H→L (91 %)

[a] On the TPSSh/TZVP level of theory; in parentheses: S_0_→S_1_ transition as predicted by MC‐PDFT. [b] *hypothetic* monomeric CN 4 species. [c] Dimeric species, **[Zn(1)]_2_**. [d] monomeric CN 5 species with axial solvent.

**Figure 5:**
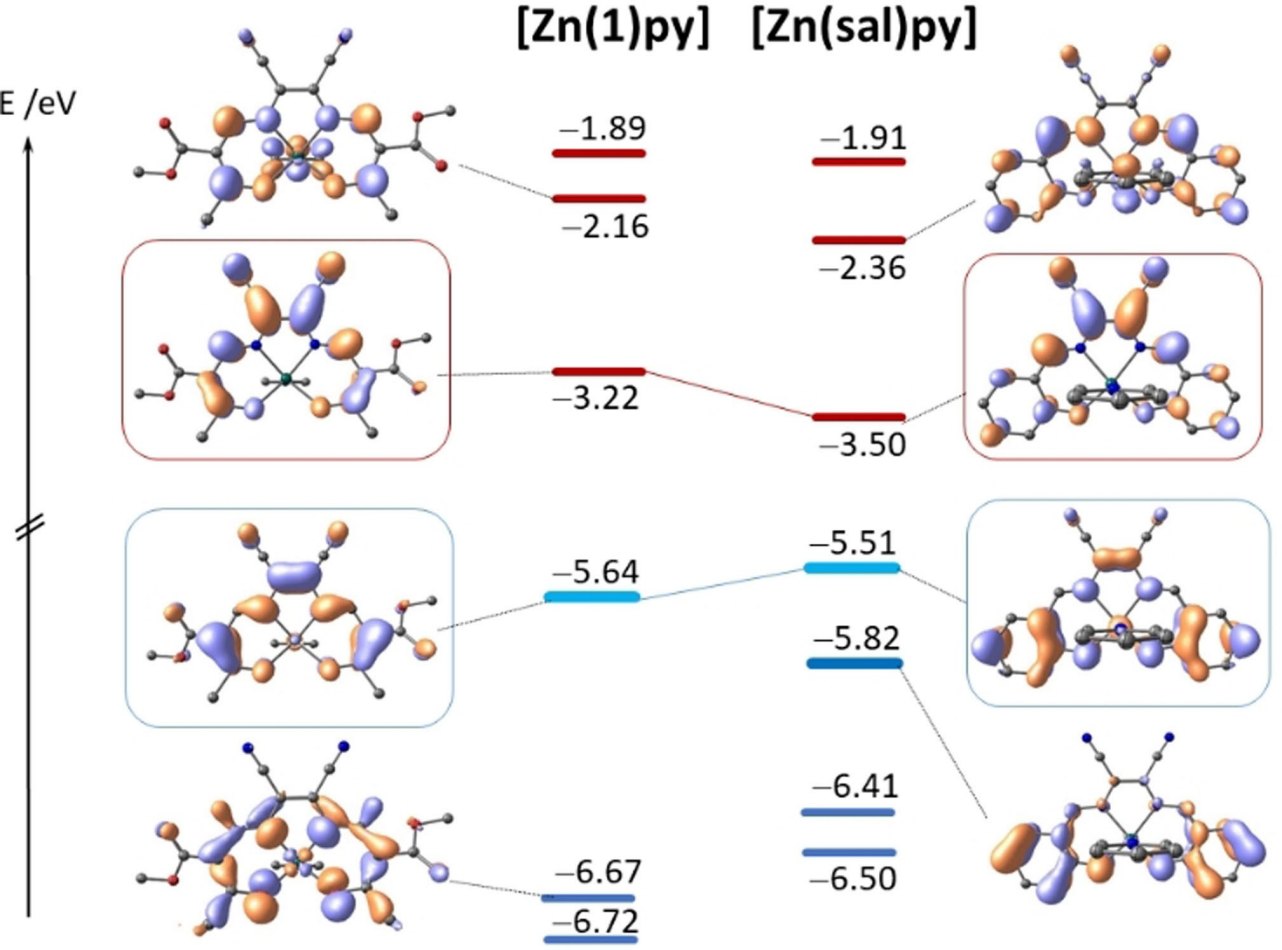
DFT‐derived frontier MOs of five‐coordinate zinc complexes (**[Zn(sal)py]** denotes an analogue derived from salicylic aldehyde; blue: occupied; red: virtual; HOMO and LUMO are highlighted.

Both at DFT and CASSCF level of theory, the HOMO and the LUMO are residing predominantly on the ligand (only at DFT level a very slight contribution of zinc d_z2_ features in the HOMO), yet with distinctly different local weights. The HOMO subsumes contributions of the ligand π‐backbone and the σ‐bound donors, whereas the LUMO is more localized on the dinitrile moiety. As a consequence, optical excitation in **[Zn(1)py]** leads to a charge shift toward the dinitrile site, rendering the excited state intra‐ligand CT‐like. Similar conclusions had been drawn previously in studies of the closely related congener **[Zn(sal)py]**, which derives from an enole/imine ligand based on salicylic aldehyde. In keeping with conserved transition energies, the axial ligand X does not feature significantly in the frontier molecular orbitals, as exemplarily shown in Figure 5 for **[Zn(1)py]** (data of the entire X‐series is summarized in Figure S20 in the Supporting Information). The optimized CASSCF natural orbitals for all states are similar to each other and to the Kohn‐Sham (KS) orbitals derived from DFT. They are reported in Figure S21 in the Supporting Information. As for the KS‐orbitals, the CAS natural orbitals do not show any significant admixture from the axial pyridine ligand. This indifference is reflected by a small decrease in the transition energies upon axial coordination. MC‐PDFT and TD‐DFT predict a red‐shift of ∼1000 cm^−1^ and ∼1400 cm^−1^, respectively, of the S_0_→S_1_ transition upon adding pyridine to the hypothetical four‐coordinate model system **[Zn(1)]**, with no axial ligands. Even smaller red‐shifts of 700–900 cm^−1^ are induced by the weaker Lewis‐bases (see Table 2). N_2_O_3_ coordination inherent to the dimeric complexes **[Zn(1)]_2_** likewise results in a red‐shifted S_0_→S_1_ transition. It is predicted at≈430 nm, irrespective of dimer structure (see also Discussion below).

Overall, both methods yield consistent results concerning the nature and energies of electronic states and transitions in the five‐coordinate species. It is not uncommon that KS‐DFT and the subsequent TD‐DFT are able to describe excited states (here, the S_1_ state) with a reasonable level of accuracy. Neither a bi‐configurational wave function nor spin‐adaptation is enforced by the method often leading to unphysical eigensolutions to the Schrödinger equation (not spin eigenstates). Nonetheless, KS‐DFT can predict accurate energies of electronic states, in virtue of the fact that DFT relies on correct local spin‐densities and not to the detailed structure of the wave function. Given the widespread utilization of KS‐DFT also beyond its inherent single‐reference limits, this aspect is discussed in the following. A more detailed treatment is given in Ref. [17c].

Inspection of the CAS wave functions of the S_0_ and S_1_ states of **[Zn(1)py]** clearly corroborates the ILCT nature of the S_0_→S_1_ transition involving a HOMO to LUMO single excitation, adding value to our DFT computations. Both S_0_ and S_1_ wave functions are predominantly single‐reference. The leading contribution to the S_0_ state (79 % weight) is the closed‐shell configuration (single‐reference) found also by KS‐DFT for the same state. In the lowest excited singlet state an electronic configuration corresponding to the HOMO‐to‐LUMO one‐electron excitation dominates with a weight of 73 %. It is important to point out here that while the S_1_ wave function is predominantly single‐configurational (in terms of spin‐adapted configuration state functions, CSFs), it is inherently bi‐configurational. That is, the two unpaired electrons residing in the HOMO and the LUMO are precisely coupled to a singlet spin state, ${\left(a_{H}^{\alpha }\right)}^{†}{\left(a_{L}^{\beta }\right)}^{†}-{\left(a_{H}^{\beta }\right)}^{†}{\left(a_{L}^{\alpha }\right)}^{†}$ (here H and L refer to HOMO and LUMO respectively). Thus, while both states can be considered single‐reference, the S_1_ excited state requires a more careful theoretical treatment, to ensure the correct spin‐symmetry and to correctly capture electron correlation effects. These aspects are explicitly considered in the CASSCF/MC‐PDFT methodology, due to the multi‐configurational character of the method, and their formulation in a spin‐adapted basis (via the graphical unitary group approach, GUGA, algorithm).^[18]^

**Figure 6:**
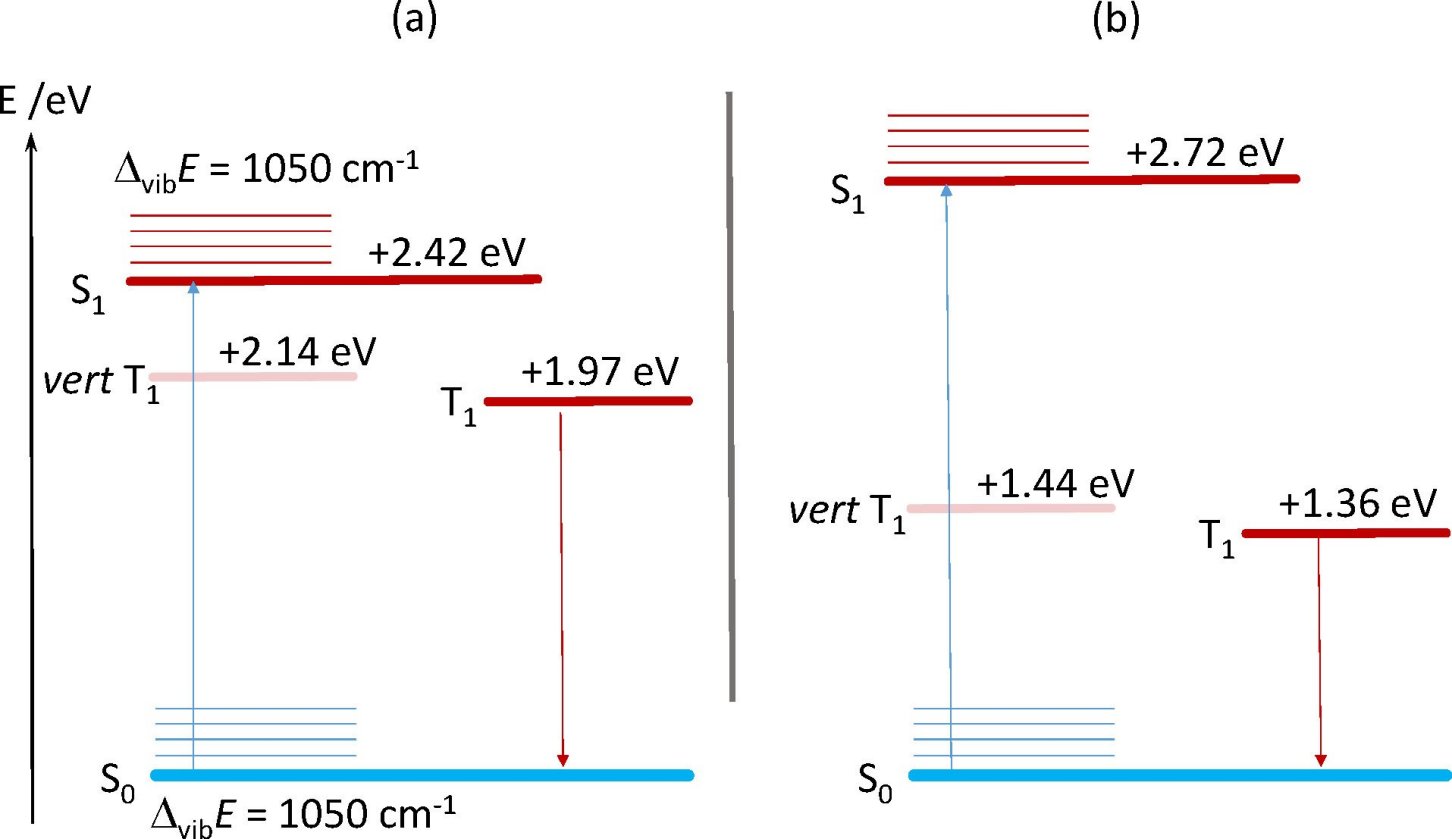
Jablonski diagrams of **[Zn(1)py]**; (a) data from MC‐PDFT (ANO‐RCC‐VDZP); vibrational splitting Δ_vib_E from experiment; (b) data from KS‐DFT and TD‐DFT (S_0_→S_1_) (TPSSh/TZVP).

The MC‐PDFT‐derived vertical and adiabatic singlet‐triplet gaps of **[Zn(1)py]**, Δ_S/T_ *E*, are reported in the Jablonski diagram in Figure 6a; they are given in terms of the S_0_→T_1_ transition energies at the relaxed geometry of S_0_ (vertical excitation) and T_1_ (adiabatic excitation). Close‐lying excited singlet and triplet states are generally taken as a basic requirement of rapid and efficient intersystem crossing, which would competitively limit the fluorescence quantum yield. A vertical gap of Δ_S/T_ *E*=2.14 eV places the triplet state only 0.28 eV below the computed energy of the Franck‐Condon state, that is, relative to S_1_ in an excited vibronic state. In the relaxed geometry of T_1_ the somewhat smaller adiabatic gap of Δ_S/T_ *E*=1.97 eV arises, concomitant with a slightly widening gap between S_1_ and T_1_. Nevertheless, these calculations indicate an efficient ISC to the triplet state as the reducing factor of the fluorescence quantum yield. In the related zinc(II) system **[ZnTPP]** (TPP: tetraphenylporphine), fluorescence and phosphorescence have been recorded at 600 nm and ca. 780 nm (77 K), respectively, giving an upper limit for the S_1_‐T_1_ gap of 0.48 eV.^[19]^ In keeping with the narrow S_1_‐T_1_ gap, a large triplet quantum yield of 0.88 was observed.

For comparison we plot in Figure 6b the data that derive from KS‐DFT and TD‐DFT. These are in qualitative agreement with the MC‐PDFT values, but indicate larger S_1_‐T_1_ splitting. We believe that a gap of 1.3 eV is hardly in agreement with efficient intersystem crossing.

In agreement with experiment the Vis transition of **[Zn(sal)py]** (λ_DFT_=565 nm; λ_exp_=560 nm^[16]^) is predicted at significantly smaller energy than in **[Zn(1)py]** (λ_DFT_=440 nm; λ_exp_=481 nm). This tendency is maintained in the (*hypothetical*, see discussion) planar CN 4 species and is reflected by the shrinking HOMO‐LUMO gap of **[Zn(sal)py]**. The presence of additional phenolate‐borne π‐donor states in the latter gives rise to further Vis transitions at higher energy that are clearly absent in **[Zn(1)py]**. Nevertheless, both complex families share similar optoelectronic properties, in a qualitative sense; that is, conserved nature and intensity of the leading excitation and significant CT‐state emission with minor Stokes‐shift. As is detailed below, however, they differ substantially in quantitative terms, with respect to their affinity for Lewis bases.

**Figure 7:**
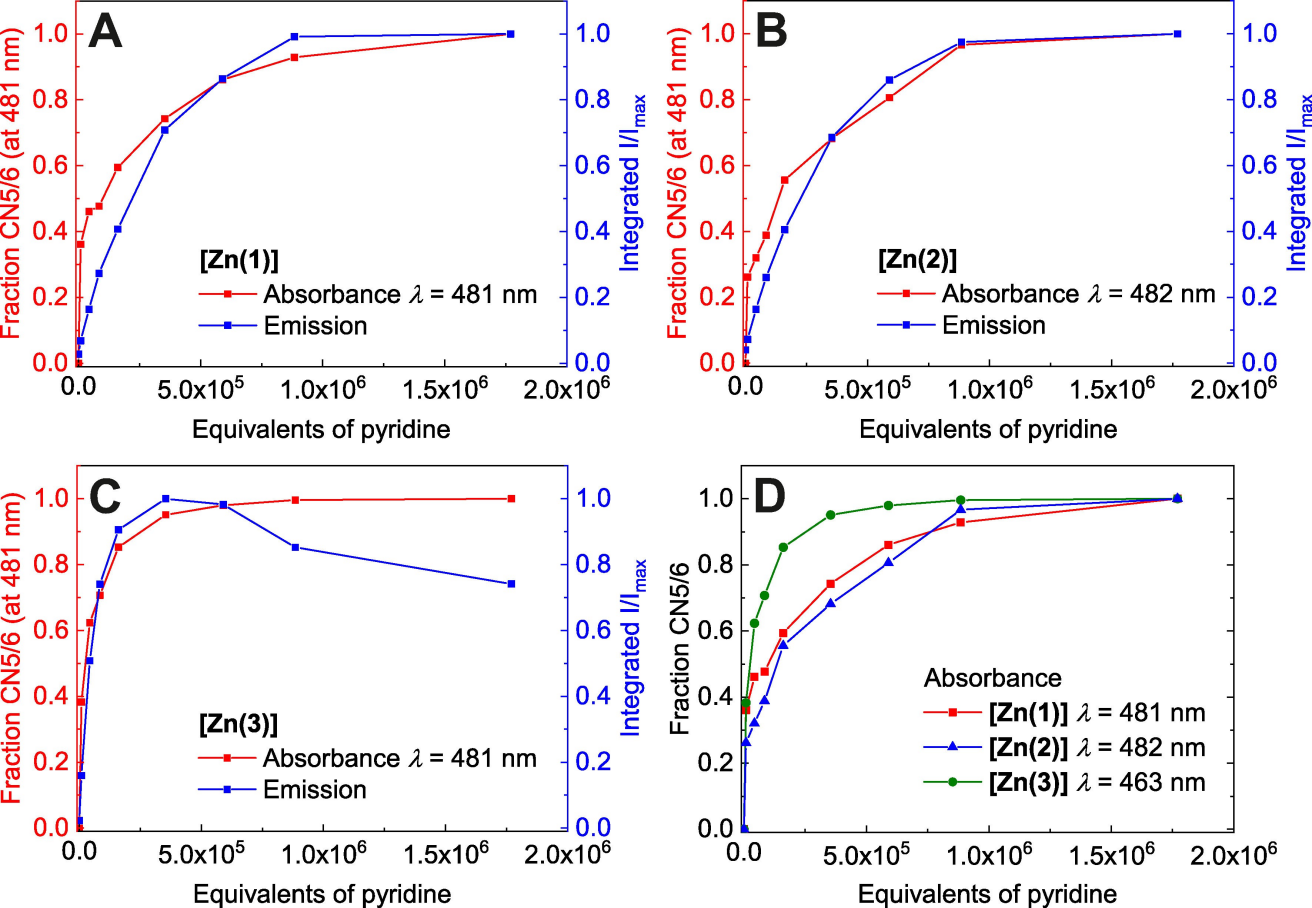
Fraction γ(CN5/6) of **[Zn(1–3)]** and integrated emission intensity I/I_max_ vs. equivalents of pyridine (**A**: **[Zn(1)]**; **B**: **[Zn(2)]**; **C**: **[Zn(3)]**). Fraction_CN5/6_=(A–A_0_)/(A_max_–A_0_) with A: Absorbance at λ=481 nm, A_0_: Absorbance in neat CHCl_3_ at λ=481 nm, and A_max_: Absorbance in neat pyridine at λ=481 nm. **D** Comparison of fractions γ(CN5/6) of **[Zn(1–3)]**.

**Quenched Lewis‐acidity and emission properties**: To discuss the optical phenomena in more quantitative terms, the concentration dependence in the spectroscopic titrations with pyridine has been analyzed. Plots of the integrated emission intensity and the differential changes of absorptivity (abstracted in terms of speciation; that is, reporting the fraction CN 5/6) as a function of pyridine concentration are shown in Figure 7.

As a first result, the above Lewis‐base hypothesis is corroborated by the higher affinity for pyridine expressed by **[Zn(3)]**. Bearing electron withdrawing CF_3_ substituents, **[Zn(3)]** is set apart quantitatively of the other zinc complexes in this study. With respect to **[Zn(1/2)]** the absorbance and emission bands of **[Zn(3)]** are all blue‐shifted by approximately 15 nm (see Figure S15B/D/F in the Supporting Information). These shifts to larger energy reflect the stabilized Schiff‐base borne donor levels (see MO diagram in Figure S22 in the Supporting Information). They further support the ILCT character of the underlying transitions. As a more diagnostic effect of enhanced Lewis acidity, the pyridine‐dependent spectral evolution of **[Zn(3)]** saturates above approximately 590 keq, whereas much larger amounts are necessary with **[Zn(1/2)]** (see Figure 7D). Similarly, enhanced affinity for Lewis‐bases has been recently reported for nickel(II) complexes of CF_3_‐decorated ligands.^[20]^ The experimentally observed order of affinity is well captured by DFT model calculations. Reaction energies of the Lewis acid‐base association of pyridine with (*hypothetical*) planar monomeric zinc complexes (CN 4) are all highly negative, indicating a significant thermodynamic driving force even if unfavorable entropies are considered (equation 1–3). Association is predicted to be more exothermic in **[Zn(3)]** than in **[Zn(1)]** by ca. 8 kJ mol^− 1^.

(1) $$\left[Zn\left(1\right)\right]+\mathrm{py}\vec{\leftarrow }\left[Zn\left(1\right)py\right];\;\Delta {}_{\mathrm{DFT}}E=-110.2\;\mathrm{kJ}\;\mathrm{mol}{}^{-1}$$

(2) $$\left[Zn\left(3\right)\right]+\mathrm{py}\vec{\leftarrow }\left[Zn\left(3\right)py\right];\;\Delta {}_{\mathrm{DFT}}E=-118.4\;\mathrm{kJ}\;\mathrm{mol}{}^{-1}$$

(3) $$\left[Zn\left(sal\right)\right]+\mathrm{py}\vec{\leftarrow }\left[Zn\left(sal\right)py\right];\;\Delta {}_{\mathrm{DFT}}E=-101.1\;\mathrm{kJ}\;\mathrm{mol}{}^{-1}$$

It is of particular importance for the following argumentation to note that computed Lewis‐base affinities of **[Zn(1)]** and **[Zn(3)]** are *larger* than that of the known system **[Zn(sal)]**, ΔΔ_LB_ *E*=Δ_LB_ *E*(**[Zn(1)]**) – Δ_LB_ *E*(**[Zn(sal)]**)≈−9 kJ mol^−1^ (for X=MeCN, pyridine, THF). That is, according to the chemistry indicated in equation 1–3 we must expect **[Zn(1)]** and **[Zn(3)]** to be more potent Lewis acids than **[Zn(sal)]**.

In clear contrast with this statement is the drastically smaller affinity of **[Zn(1)]** and **[Zn(3)]** toward axial ligation observed in experiment. Whereas ligation of mononuclear **[Zn(sal)]** and derivatives thereof has been reported to saturate already at slightly super‐stoichiometric doses of pyridine (2–10 eq),^[8b]^ *all* complexes **[Zn(1–3)]** in the present study require pyridine doses that are higher by a factor of ≫10^4^! Therefore, the central premises of our analysis must be called into question. As we discuss in the following, speciation in solution of **[Zn(sal)]** and **[Zn(1–3)]** is not identical, as it has been silently assumed.

There is general agreement that four‐coordinate planar monomeric zinc complexes (CN 4) will add one or two additional axial ligands to saturate the Lewis‐acidic center as is implied by equation 1–3. In the absence of potent donors self‐complementary stacking of such zinc(II) units occurs to yield dimers with a Zn_2_O_2_ core.^[8]^ Crystal structures including stacked dimers with a Zn_2_O_2_ core have been reported for several derivatives of the *salophen* type and dimers have been generally suggested as the dominant species in solution under non‐coordinating conditions. Such dimeric structures are generally found to have reduced fluorescence quantum yields, whereas the on‐switch of fluorescence in the presence of Lewis‐bases has been interpreted in terms of base‐induced de‐aggregation. Stacking into dimers in non‐coordinated media was likewise suggested to reduce fluorescence in *malnant* derivatives with maleodinitrile backbones.^[8d]^ The ligation equilibria of **[Zn(sal)]** could be convincingly treated in terms of a theoretical three‐component model, yielding very large equilibrium constants for both, the dimerization step and the ligation of the sub‐coordinate monomer (equation 4–5).^[21]^ Alluding to this model, speciation successively moves from one *single* non‐fluorescent species, dimeric (**[Zn(sal)]_2_**), to one *single* fluorescent species, monomeric (**[Zn(sal)py]**) (CN 5), with only vanishing contributions of four‐coordinate **[Zn(sal)]**.

(4) $$2\;\left[Zn\left(sal\right)\right]\vec{\leftarrow }\left[Zn\left(sal\right)\right]{}_{2};\;K{}_{\dim}\approx 10{}^{8}\;M{}^{-1}$$

(5) $$\left[Zn\left(sal\right)\right]+\mathrm{py}\vec{\leftarrow }\left[Zn\left(sal\right)py\right];\;K{}_{\mathrm{py}}\approx 10{}^{5}-10{}^{6}\;M{}^{-1}$$

**Figure 8:**
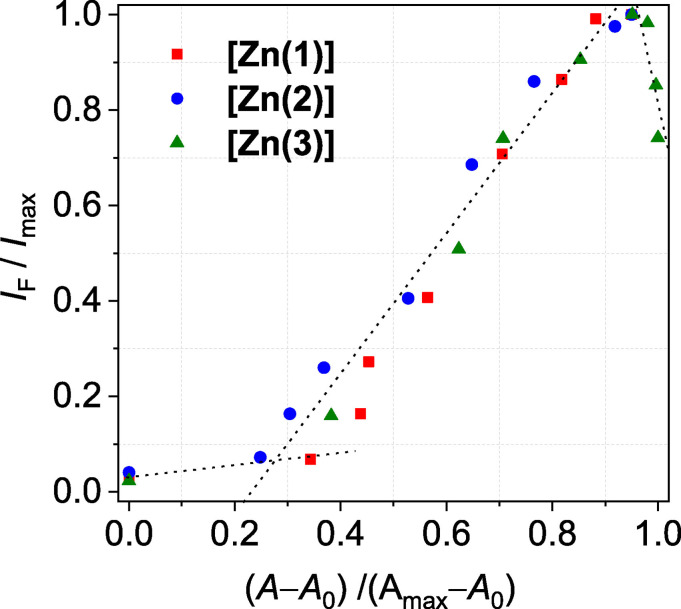
Correlation of pyridine‐dependent absorption and emission changes. Plot of the normalized integrated emission intensity vs. the CN5/6 molar fraction of **[Zn(1–3)]**. Fraction_CN5/6_=(A–A_0_)/(A_max_–A_0_) with A: Absorbance at λ=481 nm, A_0_: Absorbance in neat CHCl_3_ at λ=481 nm, and A_max_: Absorbance in neat pyridine at λ=481 nm. Fraction_5/6_ of **[Zn(1/2)]** scaled by a factor of 0.95.

Different from **[Zn(sal)]**, there is no such simple correlation for **[Zn(1–3)]**. Closer inspection of the low‐concentration sections of the plots in Figure 7A/B reveals that the concentration dependence of the absorption and the emission is not identical. In particular, the plot in Figure 8 of the normalized integrated emission intensity vs. the CN 5/6 molar fraction of **[Zn(1–3)]** reflects pyridine‐dependent transformations among (at least) four different species, from which (at least) the first two are non‐emissive. The close similarity of **[Zn(1–3)]** in the plot of Figure 8 points to an overall conserved phenomenology among all three compounds.

The first chemical step connects two non‐fluorescent species; notably almost one third of the overall absorption change is involved in this step, whereas there is hardly any increase in emission. In accordance to previous findings of non‐emissive stacked dimers,[^8b^, ^8d^] it appears reasonable to ascribe the lack of fluorescence to the dimeric or oligomeric nature of *both* non‐fluorescent species. This assignment is supported by means of dynamic light scattering (DLS) in MeCN. Solutions in CHCl_3_ are expected to behave similarly, but could not be studied by DLS due to limited solubility. Non‐emitting, filtered solutions of **[Zn(1)]** in neat MeCN (3 ⋅ 10^−4^ M < *c* <1 ⋅ 10^−3^ M) all contain nanoparticles with broad particle size distributions (*d*_max_≈120 nm) (see Figure S23 in the Supporting Information). Mean particle size scales inversely with the concentration of the complex. The presence of pyridine drastically reduces the amount of nanoparticles, whereas in neat pyridine, DLS gives no response (see Figure S24 in the Supporting Information). In keeping with the destacking of the dimers/oligomers in the presence of pyridine, and concomitant formation of fluorescent monomeric **[Zn(1–3)py]**, emission enhancement and absorption changes during the spectroscopic titrations are proportional, once a threshold concentration is passed (rising branch in Figure 8). For **[Zn(3)]** emission decreases in presence of very high doses of pyridine. We associate this unique behavior with the enhanced Lewis‐acidity of **[Zn(3)]** which allows for the formation of six‐coordinate species as seen also in the solid state molecular structure of **[Zn(3)(H_2_O)(EtOH)]** (Figure 1). While we cannot judge with absolute certaintythe decreased emission efficiency of six‐coordinate species may be associated with the higher flexibility of the octahedral coordination sphere due to the intrinsically longer Zn−N/O bonds leading to a higher rate of non‐radiative decays. The coordination of the second pyridine might also result in a decreased singlet‐triplet gap leading to emission quenching, which was found indeed in DFT calculations (see Figure S25 in the Supporting Information).

**Figure 9:**
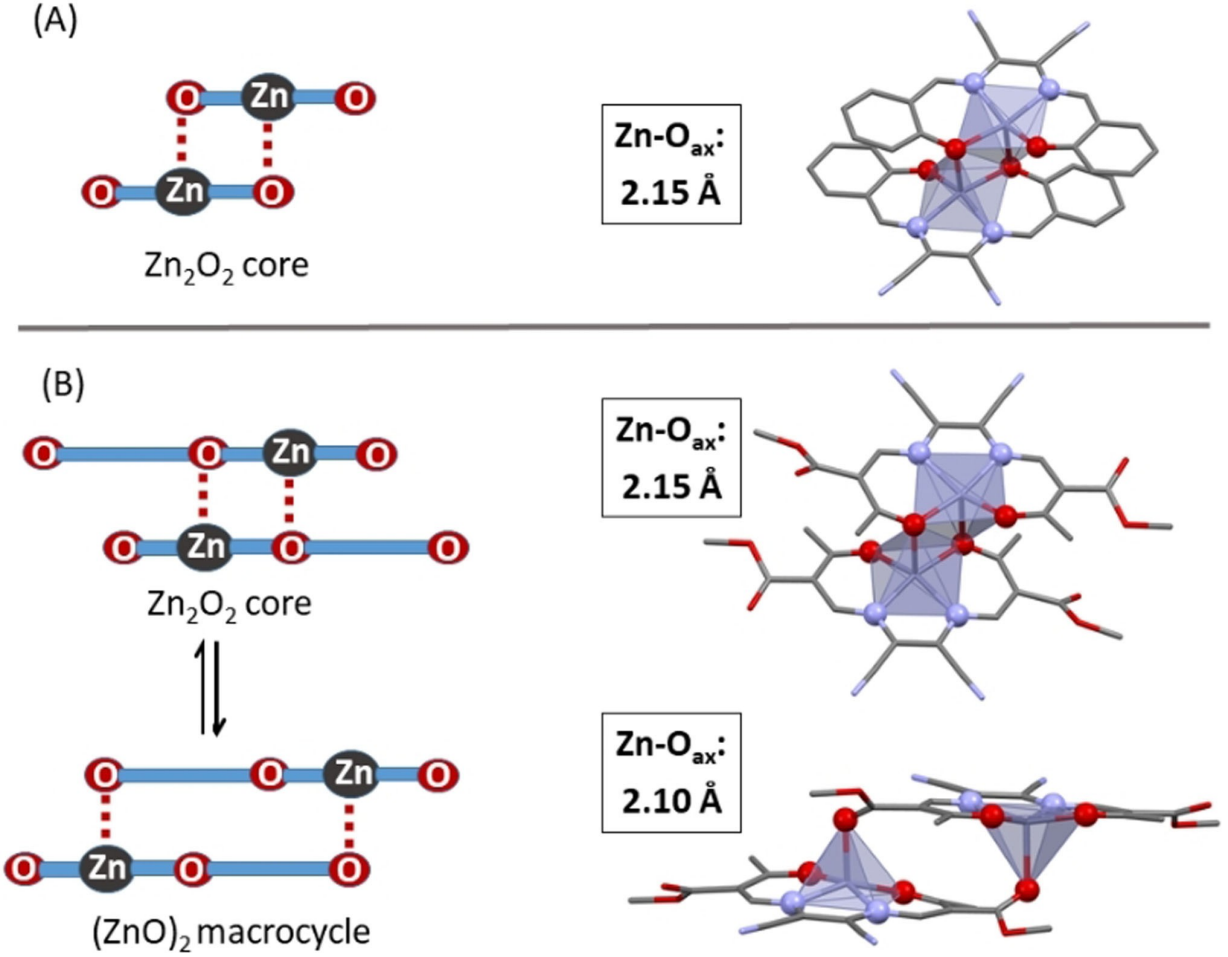
(A) Dimer speciation of **[Zn(sal)]_2_** (left) and its DFT‐optimized structures with pertinent metrics (right). (B) Dimer speciation of **[Zn(1–3)]_2_** (left) and its DFT‐optimized structures with pertinent metrics (right).

Kleij et al. had previously observed a diminished sensitivity toward Lewis‐bases of some dinuclear zinc(II) complexes.^[22]^ Akin to our observations with **[Zn(1–3)]**, >10^5^ molar equivalents of pyridine were necessary to drive the destacking. The authors associated the markedly reduced sensitivity with strong stacking into large oligomers. Based on the observation of nanoparticles in dilute solutions via DLS, we likewise associate the *seemingly* reduced Lewis‐acidity of **[Zn(1–3)]** with the formation of stable oligomers of unusual stability. That is, the strong Lewis‐acidity of **[Zn(1–3)]** which is indicated by DFT calculations must be overridden by an additional ligand‐borne effect not accessible to mononuclear *salophen* derivatives. Indeed, a DFT structure search served to identify plausible dimer structures of high stability for **[Zn(1)]_2_**. For sake of comparison, stacked structures of **[Zn(sal)]_2_** were studied on the same level of theory. The computed metrics of the Zn_2_O_2_ core of **[Zn(sal)]_2_** shown in Figure 9A are similar to the reported crystal structures of *salophen*‐type complexes.^[21a]^ Details of all optimized structures are summarized in Table S7 in the Supporting Information. In particular, the short axial Zn−O distance of 2.15 Å indicates tight binding within the dimers; electronic reaction energies of the dimerization equilibrium amount to −16 kJ mol^−1^. Similar structures could be extracted from optimization of Zn_2_O_2_‐bound dimers of **[Zn(1)]**, with similar energies of dimerization.

Most importantly, however, these Zn_2_O_2_‐core dimers are not the minimum structures in the case of **[Zn(1–3)]**. By contrast, DFT calculations identify a completely different stacking motif at significantly lower energy, as shown in Figure 9B. In these (ZnO)_2_ macrocycles, ligand‐appended carbonyls saturate the N_2_O_3_ coordination sphere of zinc to establish fully relaxed square‐pyramidal environments on both centers. A somewhat related constellation has been observed by Weber et al. in the solid‐state structure of some iron(II) complexes of this general ligand type.^[23]^ In the cited system, the axially arranged carbonyls rather assist coordination, given the long Fe−O distance of 2.70 Å. In **[Zn(1–3)]**, tight axial binding is indicated by short Zn−O_ax_ bond lengths of 2.10 Å, which are even shorter than in the reported and computed dimers of **[Zn(sal)]**. Favorable dispersive interactions between the chelate planes of the stacked complexes support the laterally shifted dimer structure. Both aspects sum up in a strongly increased stability compared to the Zn_2_O_2_‐core dimer. Computed energies of dimerization are in favor of the macrocyclic (ZnO)_2_ dimer by −20 kJ mol^−1^.

## Conclusions

The family of planar zinc(II) complex platforms, **[Zn(1–3)]**, based on planar‐directing tetradentate Schiff base‐like ligands with appended nitrile groups supports *turn‐on* fluorescence behavior, sensitive to axial ligation with potent Lewis bases. While the overall optical spectroscopic features of **[Zn(1–3)X]** comply with the established stacking/destacking hypothesis of zinc(II)‐based fluorescence sensors in qualitative terms, a massive deviation is noticed in terms of a quantitative treatment. While only 2–3 equivalents of pyridine were reported to switch‐on fluorescence in the closely related congener **[Zn(sal)]**, a large excess of ≫10^4^ equivalents is required in the case of **[Zn(1–3)]**. This enormous difference in sensitivity cannot be traced to a qualitative divergence in Lewis acidity of **[Zn(1–3)]** and **[Zn(sal)]**; in fact, KS‐DFT calculations suggested an even stronger Lewis‐acidity of **[Zn(1–3)]**. The higher intrinsic acidity of **[Zn(1–3)]** is quenched through formation of stable aggregates via axial Zn−O bonds. Qualitatively different from the established picture of a Zn_2_O_2_ core which prevails for **[Zn(sal)]** derivatives, KS‐DFT calculations of dimeric species of **[Zn(1)]** support an alternative, very stable macrocyclic stacking motif which involves ligand‐appended, remote carbonyl moieties. As the stability of the non‐emissive dimers or oligomers obviously controls the sensitivity of the sensor material, tuning through introduction of sterically demanding substituents might enable the design of sensor materials with consciously adjustable sensitivity areas.

## Experimental Section

Methoxymethylenemethylacetoacetate, ethoxymethyleneethyl‐acetoacetate, ethoxymethylene‐1,1,1‐trifluoroacetylacetone, and **H_2_(2)** were synthesised as described in literature.[^11^, ^24^] Diaminomaleonitril (98 %, Sigma Aldrich), *p*‐toluenesulfonic acid (98 %, Merck), and Zn(OAc)_2_ ⋅ 2 H_2_O (97+%, Alfa Aesar) were used without further purification. Methanol, ethanol, THF, and pyridine were of analytical grade and used without further purification. Chloroform and acetonitrile were extracted with aqueous saturated NaHCO_3_ solution and dried over CaCl_2_. NMR spectra were recorded with a 500 MHz *Avance III HD* NMR spectrometer from Bruker. CHN analyses were measured with an Unicube from Elementar Analysen Systeme. The samples were prepared in a tin boat, sulfanilamide was used as standard and the samples measured at least twice. Mass spectra were recorded with a Finnigan MAT 8500 with a data system MASPEC II. IR spectra of the solid samples were recorded on a *Perkin Elmer Spectrum* 100 FTIR spectrometer.

X‐ray Structure Analysis. The X‐ray analysis were performed with a Stoe StadiVari diffractometer using graphite‐monochromated MoK_α_ radiation. The data were corrected for Lorentz and polarization effects. The structures were solved by direct methods (SIR‐97)^[25]^ and refined by full‐matrix least‐square techniques against Fo2–Fc2 (SHELXL‐97).^[26]^ All hydrogen atoms were calculated in idealized positions with fixed displacement parameters. ORTEP‐III was used for the structure representation,^[27]^ Mercury‐3.10 to illustrate molecule packing.^[28]^ Deposition Numbers 2086160 (for **[Zn(1)(MeOH)] ⋅ MeOH**), 2086684 (for **[Zn(1)(py)]**), 2086163 (for **[Zn(1)(H**_**2**_ **O)]**), 2086161 (for **[Zn(2)(H**_**2**_ **O)]2⋅H**_**2**_ **O**), 2086162 (for **[Zn(3)(H**_**2**_ **O)(EtOH)]**) contain the supplementary crystallographic data for this paper. These data are provided free of charge by the joint Cambridge Crystallographic Data Centre and Fachinformationszentrum Karlsruhe Access Structures service.

X‐ray Powder Diffraction. Powder diffractograms were recorded with a STOE StadiP diffractometer using Cu K*α*1 radiation with a Ge monochromator, and a Mythen 1 K Stripdetector in transmission geometry.

Optical Measurements. Absorbance spectra were performed on a Cary 60 UV‐Vis spectrometer from Agilent Technologies. Steady‐state PL measurements were performed on a FP‐8600 fluorescence spectrometer from JASCO that is equipped with a Xe lamp as excitation source. Time‐resolved measurements were performed on a FluoTime 300 fluorospectrometer from PicoQuant, using a 405 nm diode laser for excitation (Coherent COMPASS 405–50 CW), which was controlled by the PDL 820 PicoQuant laser driver. Quantum yields were determined at room temperature using an integrating sphere and a Xe lamp as excitation source. All measurements were performed in quartz cells with a 1 cm light path from Hellma.

**H_2_(1)**. Diaminomaleonitrile (1.50 g, 13.88 mmol, 1 eq), methoxymethylene‐methylacetoacetate (4.83 g, 30.53 mmol, 2.2 eq), and *p*‐toluenesulfonic acid (0.13 g, 0.69 mmol, 0.05 eq) were dissolved in 37 mL MeOH. The red solution was heated under reflux for 5 h. After cooling at room temperature overnight the orange precipitate was filtered off and washed with MeOH. Yield: 2.18 g (44 %). ^1^H NMR (500 MHz, DMSO, 25 °C): δ=8.11 (s, 2 H, NC−H); 3.74 (s, 6 H, −CH_3_); 2.44 (s, 6 H, −CH_3_) ppm. MS (DEI‐(+), 70 eV): m/z=360 (M^+^, 36 %). C_16_H_16_N_4_O_6_ (360.33 g/mol) found (calculated): C 53.18 (53.33); H 4.33 (4.48); N 15.31 (15.55)%. IR: $\tilde{\nu }$ =2954 (s, C−H), 2228 (s, C≡N), 1719 (s, C=O), 1585 (s, C=O) cm^−1^.

**[Zn(1)(H_2_O)(MeOH)]**. H_2_(1) (0.40 g, 1.11 mmol, 1 eq) and zinc(II) acetate dihydrate (0.32 g, 1.44 mmol, 1.3 eq) were dissolved in 40 mL MeOH. The solution was heated to reflux for 2 h. After cooling to room temperature and addition of 20 mL H_2_O, the red crystalline precipitate was filtered off and washed with MeOH. Yield: 0.37 g (70 %). ^1^H NMR (500 MHz, DMSO, 25 °C): δ=8.51 (s, 2 H, NC−H); 3.69 (s, 6 H, −CH_3_); 2.49 (s, 6 H, −CH_3_) ppm. MS (DEI‐(+), 70 eV): m/z=422 (M^+^, 58 %). C_17_H_20_N_4_O_8_Zn (473.75 g/mol) found (calculated): C 42.97 (43.10); H 4.11 (4.26); N 11.98 (11.83)%. IR: $\tilde{\nu }$ =3394 (b, O−H), 2954 (s, C−H), 2217 (s, C≡N), 1677 (s, C=O), 1574 (s, C=O) cm^−1^.

**[Zn(2)(H_2_O)_1.5_]**. H_2_(2) (0.40 g, 1.03 mmol, 1 eq) and zinc(II) acetate dihydrate (0.29 g, 1.34 mmol, 1.3 eq) were dissolved in 40 mL MeOH. The solution was heated to reflux for 2 h. After cooling to room temperature and addition of 20 mL H_2_O, the orange precipitate was filtered off and washed with MeOH. Yield: 0.23 g (47 %). ^1^H NMR (500 MHz, DMSO, 25 °C): δ=8.53 (s, 2 H, NC−H); 4.16 (q, ^3^ *J*(CH_2_−CH_3_)=7.0 Hz, 4 H, −CH_2_); 2.49 (s, 6 H, −CH_3_); 1.25 (t, ^3^ *J*(CH_2_−CH_3_)=7.0 Hz, 6 H, −CH_3_) ppm. MS (DEI‐(+), 70 eV): m/z=450 (M^+^, 100 %). C_18_H_21_N_4_O_7.5_Zn (478.77 g/mol) found (calculated): C 45.37 (45.16); H 4.24 (4.42); N 11.68 (11.70)%. IR: $\tilde{\nu }$ =3311 (b, O−H), 2987 (s, C−H), 2217 (s, C≡N), 1674 (s, C=O), 1583 (s, C=O) cm^−1^.

**[Zn(3)(H_2_O)(EtOH)]**. Diaminomaleonitrile (0.05 g, 0.46 mmol, 1 eq), ethoxymethylene‐1,1,1‐trifluoroacetylacetone (0.21 g, 1.02 mmol, 2.2 eq), and zinc(II) acetate dihydrate (0.13 g, 0.60 mmol, 1.3 eq) were dissolved in 2.5 mL EtOH. The solution was heated to reflux for 2 h. After cooling to room temperature, 1 mL H_2_O was added. After storing in the fridge overnight, the red precipitate was filtered off and washed with EtOH. Yield: 0.17 g (66 %). ^1^H NMR (500 MHz, DMSO, 25 °C): δ=8.35 (s, 2 H, NC−H); 2.54 (s, 6 H, −CH_3_) ppm. MS (DEI‐(+), 70 eV): m/z=498 (M^+^, 30 %), 429 (M^+^‐CF_3_, 53 %). C_18_H_16_F_6_N_4_O_6_Zn (563.72 g/mol) found (calculated): C 38.32 (38.35); H 2.84 (2.86); N 9.98 (9.94)%. IR: $\tilde{\upsilon }$ =3511 (b, O−H), 3355 (b, O−H), 2987 (s, C−H), 2224 (s, C≡N), 1597 (s, C=O), 1538 (s, C=O), 1116 (s, C−F) cm^−1^.

### Computational Details

This section describes the most relevant details of DFT and CASSCF model calculations.

**(TD‐)DFT**. Electronic structure calculations on the complexes have been performed through density‐functional theory (DFT) methods using the ORCA program package.^[29]^ For all optimizations triple‐ξ‐valence TZVP basis sets^[30]^ were used with the generalized gradient approximated functional BP86.^[31]^ Optimized complexes were verified as stationary points through the absence of imaginary modes in numerical frequency calculations. Molecular orbitals and electronic properties were extracted from single‐point calculations in the optimized positions with the global hybrid functional TPSSh^[32]^ and triple‐ξ‐valence TZVP basis sets. Grimme's third generation D3 correction of dispersion was used;^[33]^ medium effects were approximated in a dielectric continuum approach (COSMO), parameterized for MeCN.^[34]^ Coordinates of the computed structures are assembled in the Supporting Information file COORDINATES, frontier orbital landscapes are shown in Figures S19–S21/22 in the Supporting Information. For each complex the 70–80 lowest optical electronic transitions were assessed with ORCA implemented TD‐DFT methods within the Tamm‐Dancoff approximation.

**CASSCF/MC‐PDFT**. Considering the marginal role of the closed‐shell Zn(II) metal center in terms of correlation effect, as discussed above, the active space chosen for all CASSCF calculations contains MOs predominantly with character of the π‐system of the tetra‐dentate ligand, CAS(18,16), where the 16 orbitals are linear combinations of the 16 2p AOs on the conjugated system of the ligand. State‐averaged calculations with 2 roots were performed for the singlet spin system (S_0_ and S_1_), while only the ground state for the triplet spin symmetry, T_1_.

MC‐PDFT has been largely demonstrated a good and cheap alternative to CASPT2 and it has been utilized as main method of choice in this investigation to recover dynamic correlation outside the active space. Within MC‐PDFT the tPBE translated functional has been chosen, that in our experience outperforms the other translated functional available to date.

A basis set of generally contracted atomic natural orbital (ANO‐RCC) type has been used for all atoms, obtained from the C,N,O(14 s,9p,4d), H(8 s,4p) and Zn(21 s,15p,10d,6 f) primitive functions, contracted to C,N,O(3 s,2p,1d), H(2 s,1p), Zn(5 s,4p,2d,1 f) functions, giving a basis set of split‐valence double‐ζ plus polarization quality (VDZP). This choice of basis set leads to a total of 577 basis functions for the **[Zn(1)py]** model system. Intermediate calculations in a minimal basis set (MB), with contraction scheme C,N,O(2 s,1p), H(1 s), Zn(4 s,3p,1d), have been performed as aid in the choice of the active space for all model system and spin states. The basis set has been subsequently expanded to the VDZP and the MB optimized orbitals augmented and used as starting orbitals for the VDZP CASSCF(18,16) optimization. C1 point group symmetry has been utilized in line with the preceding DFT method. The evaluation of the electron repulsion integrals has been greatly simplified by means of the resolution‐of‐identity Cholesky decomposition technique, with a decomposition threshold of 10^−4^ a.u.

## Conflict of interest

The authors declare no conflict of interest.

## Supporting information

As a service to our authors and readers, this journal provides supporting information supplied by the authors. Such materials are peer reviewed and may be re‐organized for online delivery, but are not copy‐edited or typeset. Technical support issues arising from supporting information (other than missing files) should be addressed to the authors.

Supporting Information

Supporting Information

Supporting Information
