# Supplementary figures and images for: Quenched Lewis Acidity: Studies on the Medium Dependent Fluorescence of Zinc(II) Complexes
Source: Chemistry. 2021 Oct 4;27(61):15159–71. doi: 10.1002/chem.202102086 (PMC8596774; doi:10.1002/chem.202102086)

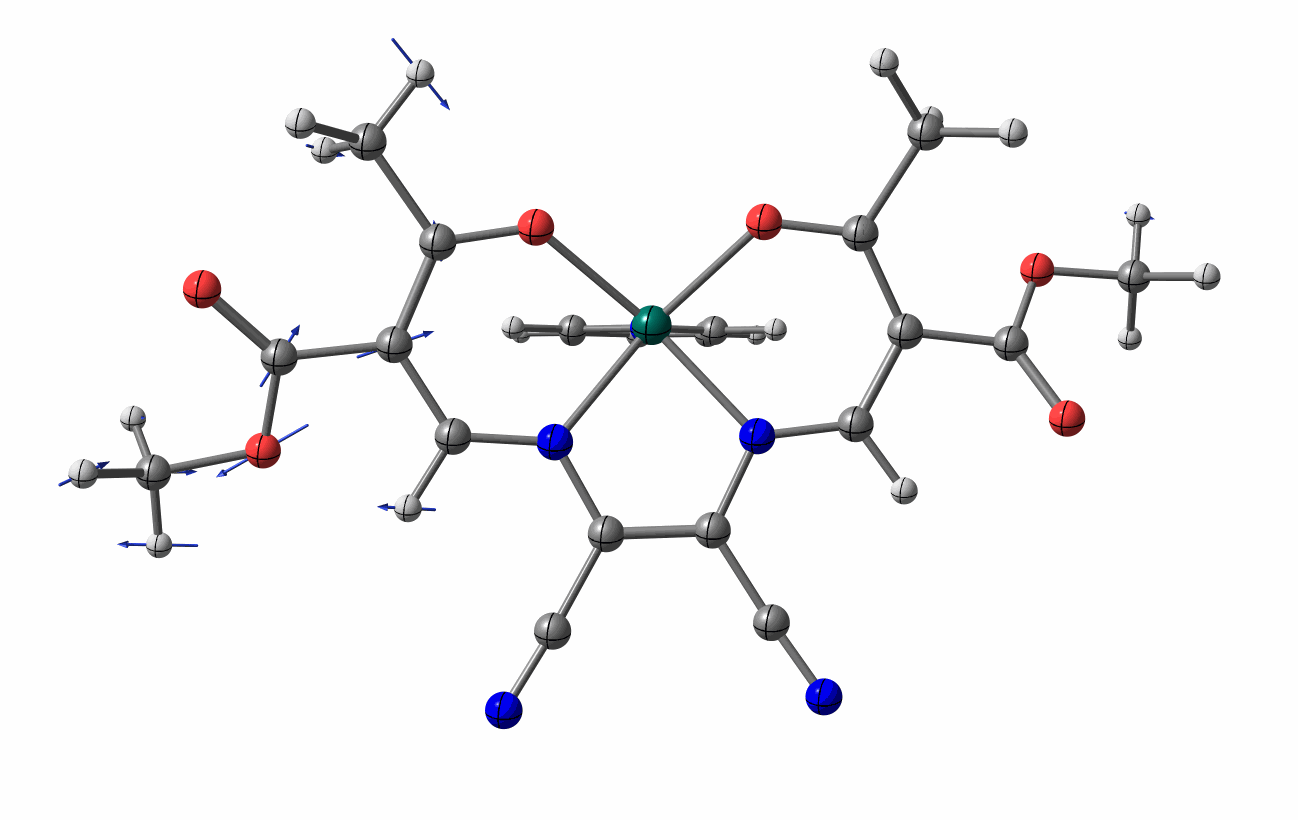

Supplement: Supplementary file 1 — Supporting Information [file CHEM-27-15159-s001.gif]
