# Supplementary material for: Quenched Lewis Acidity: Studies on the Medium Dependent Fluorescence of Zinc(II) Complexes
Source: Chemistry. 2021 Oct 4;27(61):15159–71. doi: 10.1002/chem.202102086 (PMC8596774; doi:10.1002/chem.202102086)
Supplement: Supplementary file 2 — Supporting Information [file CHEM-27-15159-s003.pdf]

[Zn(1)]; *S* = 0.

41
Coordinates from ORCA-job Zn

C	0.296218	5.107803	14.667248
C	0.110054	6.534605	14.697708
C	0.195956	7.380277	13.575088
H	0.054238	8.450758	13.761884
C	0.477941	7.860922	11.260405
C	0.696610	7.317813	9.991986
C	1.007511	5.308200	8.752588
H	1.069657	5.897844	7.828690
C	1.122164	3.914278	8.593017
C	1.131343	2.964011	9.671108
C	0.270782	4.273774	15.917821
C	1.407368	1.500541	9.456922
H	2.190873	1.327995	8.709954
H	0.499635	0.998874	9.090275
H	1.691941	1.060325	10.419373
C	1.279233	3.523277	7.164313
C	0.964126	1.839791	5.515420
H	0.402667	2.492557	4.835936
H	0.558026	0.824718	5.499677
H	2.025531	1.838858	5.238561
C	0.325871	9.262577	11.440487
C	0.770751	8.149188	8.841890
N	0.417100	6.985266	12.325157
N	0.829331	5.947008	9.903267
N	0.194076	10.406382	11.638945
N	0.836821	8.790354	7.867420
O	0.524611	4.448361	13.591744
O	0.947653	3.292865	10.897826
O	1.749164	4.262221	6.301372
O	0.802598	2.288728	6.888952
Zn	0.678671	5.060585	11.725005
H	0.694364	3.290227	15.685387
H	-0.765695	4.150989	16.263654
H	0.821140	4.745416	16.740878
H	0.218567	8.853877	18.061386
O	0.139184	8.510448	15.989602
C	-0.223383	7.198069	15.985173
C	-0.256351	9.268418	17.163842
H	0.096604	10.286588	16.976726
O	-0.773526	6.666792	16.944144
H	-1.347645	9.250708	17.278199

[Zn(1)]; *S* = 1.

41
Coordinates from ORCA-job Zn

C	0.714586	5.203135	14.616493
C	0.209643	6.551515	14.646504
C	0.143164	7.426130	13.543064
H	-0.130816	8.465968	13.745199
C	0.540197	7.946105	11.249287
C	0.793927	7.361354	9.915388
C	1.172775	5.323520	8.738126
H	1.381020	5.878281	7.816374
C	1.107397	3.919780	8.630519
C	0.777237	3.007348	9.692151
C	0.876476	4.382015	15.863707
C	0.833688	1.514116	9.522799
H	1.663983	1.192989	8.883183
H	-0.094354	1.153974	9.053211
H	0.922948	1.058602	10.515667
C	1.423662	3.448023	7.249970
C	1.010888	1.827430	5.561133
H	0.754030	2.571135	4.796718
H	0.381412	0.938952	5.465643
H	2.072467	1.562650	5.482282
C	0.493190	9.338265	11.428920
C	0.795904	8.170163	8.767081
N	0.391827	7.074316	12.273252
N	0.971026	6.020573	9.865297
N	0.452875	10.497498	11.611847
N	0.795741	8.823076	7.791256
O	1.106683	4.621310	13.536957
O	0.437819	3.395330	10.873548
O	2.204488	4.025401	6.498354
O	0.721952	2.353741	6.886533
Zn	0.718595	5.152197	11.689423
H	1.581233	3.569426	15.651806
H	-0.091899	3.945147	16.151393
H	1.223728	4.976811	16.716880
H	-0.180268	8.688141	18.104285
O	-0.108970	8.447836	16.017676
C	-0.282171	7.103635	15.939165
C	-0.661738	9.084490	17.201976
H	-0.440100	10.148880	17.081799
O	-0.807491	6.447897	16.831808
H	-1.744975	8.915132	17.252353

[Zn(1)py]; *S* = 0.

52
Coordinates from ORCA-job Zn

C	0.106567	5.051300	14.748717
C	0.152970	6.493916	14.810850
C	0.267546	7.329250	13.676424
H	0.168679	8.405288	13.861039
C	0.439389	7.782319	11.355817
C	0.599661	7.234665	10.079952
C	0.893416	5.239704	8.827187
H	0.941011	5.828203	7.900674
C	0.983607	3.842055	8.660853
C	0.745560	2.886216	9.714179
C	-0.194057	4.210717	15.960190
C	0.523797	1.422581	9.427324
H	1.493576	0.909183	9.349905
H	-0.010793	1.249829	8.486520

H -0.032442 0.991708 10.268756
C 1.243555 3.445989 7.254562
C 2.113184 1.783512 5.791675
H 1.174460 1.708515 5.228699
H 2.573893 0.798479 5.908095
H 2.797244 2.473462 5.281363
C 0.245347 9.181442 11.526212
C 0.578392 8.065289 8.925103
N 0.449852 6.918311 12.429528
N 0.752861 5.869220 9.984733
N 0.085066 10.322129 11.723712
N 0.571366 8.701034 7.944572
O 0.318787 4.395761 13.675132
O 0.648251 3.213774 10.943393
O 0.991362 4.148496 6.276164
O 1.855371 2.241535 7.145976
Zn 0.993594 4.994553 11.857442
H -0.113211 3.155329 15.675126
H -1.202318 4.422641 16.341728
H 0.497578 4.436458 16.783027
H 0.768233 8.834606 18.093917
O 0.422244 8.481745 16.050794
C -0.010640 7.186009 16.110220
C 0.213440 9.267535 17.252139
H 0.596864 10.263397 17.011064
O -0.465953 6.706374 17.144935
H -0.854948 9.314454 17.498555
N 3.042637 4.951283 12.132619
C 3.859300 4.532892 11.141381
C 3.586392 5.375744 13.293738
H 2.889692 5.709544 14.063469
C 4.961802 5.392857 13.505804
H 5.358238 5.743043 14.458108
H 3.377603 4.204476 10.219386
C 5.244220 4.520608 11.277915
H 5.866022 4.175249 10.452805
C 5.806200 4.957601 12.480859
H 6.888432 4.959487 12.617573

[Zn(1)py]; *S* = 1.

52
Coordinates from ORCA-job Zn
C 0.073785 5.056733 14.719137
C 0.130409 6.500371 14.789172
C 0.222961 7.363452 13.673677
H 0.148695 8.435913 13.879793
C 0.359692 7.840377 11.359454
C 0.531999 7.262243 10.008548
C 0.855873 5.258679 8.784907
H 0.945344 5.831922 7.854362
C 0.954555 3.856645 8.660935
C 0.683144 2.906134 9.713908
C -0.136857 4.202930 15.939705
C 0.507161 1.435890 9.436344
H 1.486080 0.933174 9.450760
H 0.052547 1.236134 8.459343
H -0.111879 1.010994 10.236223
C 1.282049 3.423027 7.275601
C 2.331620 1.776191 5.918226
H 1.415171 1.586688 5.345489
H 2.877179 0.845196 6.095244
H 2.960444 2.500230 5.384766
C 0.187265 9.225719 11.522840
C 0.537980 8.091730 8.874248
N 0.364408 6.975912 12.399380
N 0.666330 5.919350 9.933588
N 0.040706 10.379099 11.692320
N 0.547624 8.757599 7.906264
O 0.208730 4.418647 13.621030
O 0.517024 3.255439 10.931412
O 0.985470 4.055854 6.264914
O 2.002537 2.278485 7.242393
Zn 0.893918 5.039264 11.817841
H 0.007637 3.154671 15.653182
H -1.152518 4.341398 16.337336
H 0.551368 4.474213 16.750969
H 0.918121 8.683490 18.143863
O 0.565704 8.417720 16.088866
C 0.014167 7.171499 16.109537
C 0.416876 9.189985 17.309668
H 0.895080 10.150647 17.098670
O -0.509062 6.705329 17.117216
H -0.645657 9.329763 17.545006
N 2.930553 4.974363 12.092941
C 3.741260 4.524866 11.110651
C 3.476296 5.403941 13.251200
H 2.784060 5.762206 14.013849
C 4.850780 5.395855 13.468207
H 5.249658 5.751137 14.417512
H 3.257293 4.193370 10.190927
C 5.124772 4.486967 11.254157
H 5.742538 4.117701 10.436439
C 5.690308 4.929423 12.453227
H 6.771703 4.911198 12.594664

[Zn(1)(py)$_2$]; *S* = 0.

63
Coordinates from ORCA-job Zn
C 0.299838 5.210385 14.881112
C 0.187991 6.651023 14.797728
C 0.575817 7.429862 13.683395
H 0.493551 8.516167 13.815162
C 1.340335 7.803495 11.476403
C 1.510369 7.249537 10.201840
C 1.292180 5.321717 8.850340
H 1.412109 5.952053 7.958071
C 1.119839 3.945530 8.581074
C 0.925301 2.926371 9.589179
C -0.087193 4.461421 16.132613
C 0.479204 1.526842 9.234974
H 1.345041 0.919233 8.934152
H -0.232920 1.508214 8.401769

H	0.031791	1.080779	10.131346
C	1.127740	3.653229	7.128285
C	1.469690	2.029171	5.423952
H	0.464379	2.173533	5.007345
H	1.753600	0.973353	5.388869
H	2.190344	2.641700	4.867815
C	1.538344	9.197852	11.686403
C	1.898241	8.076209	9.108721
N	0.980398	6.968607	12.509596
N	1.297477	5.899606	10.041274
N	1.683762	10.333532	11.923321
N	2.212576	8.705357	8.174908
O	0.770495	4.496145	13.943154
O	1.086903	3.145413	10.828351
O	0.885049	4.483041	6.249877
O	1.489778	2.378927	6.832110
Zn	1.026476	4.937219	11.913347
H	0.312916	3.444088	16.057485
H	-1.182516	4.416824	16.220446
H	0.278344	4.951077	17.043386
H	-0.331246	9.159927	17.984408
O	0.039701	8.702764	15.966299
C	-0.383200	7.402603	15.937234
C	-0.571134	9.537506	16.982004
H	-0.137933	10.531046	16.833761
O	-1.162923	6.960351	16.778077
H	-1.660872	9.568263	16.850001
N	3.178586	4.825618	12.203223
C	3.979096	4.384366	11.212870
C	3.746114	5.364727	13.300118
H	3.060858	5.711955	14.073324
C	5.127032	5.478736	13.450007
H	5.539827	5.922573	14.355798
H	3.477173	3.960540	10.342988
C	5.368870	4.460871	11.282796
H	5.975032	4.091262	10.455896
C	5.954967	5.020251	12.421720
H	7.039947	5.098561	12.506083
C	-3.915343	4.656021	11.471440
H	-5.000210	4.598099	11.369919
C	-3.218774	5.780063	11.020085
C	-3.198919	3.606931	12.054634
H	-3.735780	6.622068	10.559774
C	-1.832909	5.811895	11.167176
H	-3.701361	2.711619	12.420476
C	-1.813919	3.719786	12.163838
N	-1.140423	4.802765	11.728303
H	-1.250024	6.668873	10.826887
H	-1.211661	2.928790	12.611627

[Zn(1)(py)$_2$]; *S* = 1.

63

Coordinates from ORCA-job Zn

C	0.276604	5.195693	14.853565
C	0.221904	6.643623	14.804895
C	0.546540	7.456469	13.693969
H	0.503386	8.537979	13.864891
C	1.162326	7.866213	11.450025
C	1.357757	7.276183	10.106238
C	1.243125	5.312186	8.797372
H	1.407473	5.912018	7.893334
C	1.117752	3.920389	8.585210
C	0.881369	2.916139	9.601432
C	-0.034619	4.435711	16.119046
C	0.505883	1.497923	9.243217
H	1.412893	0.895589	9.085777
H	-0.094118	1.431643	8.328546
H	-0.047986	1.074189	10.090213
C	1.224441	3.560245	7.146003
C	1.890484	1.921329	5.556318
H	0.892839	1.876147	5.101725
H	2.343118	0.926699	5.596543
H	2.524756	2.614218	4.989184
C	1.255730	9.259310	11.623870
C	1.694448	8.104778	9.020170
N	0.865058	7.026456	12.467722
N	1.177084	5.942464	9.974043
N	1.331789	10.417258	11.811379
N	1.981803	8.763438	8.090036
O	0.633258	4.486892	13.861414
O	0.938902	3.169421	10.845076
O	0.887412	4.297815	6.220919
O	1.793962	2.348559	6.942146
Zn	0.895997	4.991596	11.857909
H	0.352200	3.416633	16.004754
H	-1.122430	4.392858	16.276107
H	0.393753	4.907328	17.011584
H	0.066494	8.974344	18.162321
O	0.326909	8.614644	16.107562
C	-0.235704	7.377671	16.011937
C	-0.160027	9.443063	17.196097
H	0.374678	10.391388	17.094333
O	-1.042313	6.960192	16.838503
H	-1.242799	9.596709	17.103391
N	3.059040	4.877513	12.151257
C	3.864937	4.425371	11.169991
C	3.623576	5.395054	13.260465
H	2.938064	5.753507	14.028158
C	5.004186	5.476056	13.431846
H	5.412996	5.902888	14.347541
H	3.370441	4.019051	10.287502
C	5.254813	4.470256	11.260580
H	5.863970	4.093335	10.439269
C	5.836780	5.006436	12.412614
H	6.921892	5.058292	12.514135
C	-4.045654	4.743821	11.397714
H	-5.130171	4.686454	11.293369
C	-3.348564	5.873151	10.960607
C	-3.329641	3.688919	11.970736
H	-3.864596	6.720083	10.508539
C	-1.963144	5.903901	11.110506
H	-3.832404	2.789238	12.325310
C	-1.945111	3.800270	12.084134
N	-1.271446	4.888840	11.661629
H	-1.381187	6.765753	10.780746

H	-1.343089	3.004751	12.523143

[Zn(1)(OH_2)]; *S* = 0.

44

Coordinates from ORCA-job Zn

C	0.292527	5.113577	14.721188
C	0.202244	6.553315	14.762659
C	0.246545	7.388432	13.625821
H	0.090079	8.457729	13.809639
C	0.355454	7.849342	11.299527
C	0.495250	7.302436	10.020603
C	0.820643	5.305849	8.771346
H	0.838466	5.890718	7.841737
C	0.939973	3.911152	8.611711
C	0.763143	2.952460	9.673938
C	0.172743	4.272917	15.964227
C	0.610092	1.477659	9.399765
H	1.602547	1.014385	9.297400
H	0.060749	1.272100	8.474135
H	0.098787	1.023191	10.256886
C	1.176078	3.516085	7.199287
C	2.065585	1.881950	5.717162
H	1.113397	1.769920	5.183877
H	2.565220	0.914943	5.823797
H	2.708082	2.593847	5.183598
C	0.122014	9.240824	11.475659
C	0.425312	8.127079	8.863819
N	0.426500	6.986741	12.373897
N	0.681343	5.941025	9.927447
N	-0.069298	10.376378	11.675351
N	0.379111	8.759578	7.882291
O	0.486921	4.456084	13.644024
O	0.661037	3.279932	10.903885
O	0.866587	4.202930	6.226479
O	1.832380	2.337520	7.077205
O	3.030666	4.983148	11.910735
Zn	0.918823	5.071700	11.779887
H	0.442072	3.241669	15.708488
H	-0.857378	4.302811	16.346813
H	0.813001	4.646860	16.773366
H	0.599405	8.925706	18.065420
O	0.356994	8.549294	16.011824
C	-0.025254	7.238861	16.057688
C	0.061771	9.330150	17.198861
H	0.409852	10.341315	16.968287
O	-0.503717	6.739781	17.072305
H	-1.017259	9.328706	17.399173
H	3.388745	4.434095	12.633088
H	3.505869	5.832403	11.972725

[Zn(1)(MeOH)]; *S* = 0.

47

Coordinates from ORCA-job Zn

C	0.321985	5.110331	14.764213
C	0.191436	6.546301	14.793224
C	0.265757	7.379001	13.656625
H	0.107409	8.448958	13.834920
C	0.454546	7.842506	11.338161
C	0.611930	7.300162	10.059745
C	0.875936	5.300766	8.801551
H	0.896645	5.888386	7.873712
C	0.961030	3.904088	8.635337
C	0.763415	2.947542	9.694662
C	0.199226	4.274404	16.010150
C	0.566948	1.478820	9.418124
H	1.544777	0.988099	9.304143
H	0.002720	1.293009	8.497210
H	0.051159	1.035792	10.278555
C	1.182392	3.507560	7.220974
C	2.023683	1.853356	5.733238
H	1.068475	1.770294	5.200012
H	2.495583	0.872173	5.836381
H	2.685872	2.548126	5.201179
C	0.258502	9.240424	11.513999
C	0.585667	8.131541	8.906571
C	3.858755	4.488076	10.908821
N	0.475130	6.975166	12.409848
N	0.767599	5.934002	9.961825
N	0.097875	10.380875	11.712067
N	0.573999	8.769503	7.927510
O	0.566144	4.451777	13.697108
O	0.681833	3.275597	10.926662
O	0.890301	4.206039	6.251237
O	1.804217	2.311210	7.094681
O	3.061451	4.993949	12.016356
Zn	0.964632	5.059358	11.821835
H	4.921532	4.678326	11.104145
H	3.544172	5.042413	10.019236
H	3.683763	3.413593	10.762241
H	0.528021	3.255793	15.774632
H	-0.845082	4.255496	16.352611
H	0.789430	4.685693	16.838602
H	0.412681	8.919366	18.111969
O	0.269684	8.543709	16.048855
C	-0.101410	7.230520	16.076242
C	-0.086366	9.321061	17.221488
H	0.265229	10.334839	17.008502
O	-0.621146	6.723943	17.066587
H	-1.173687	9.311946	17.370483
H	3.360428	4.563870	12.838952

[Zn(1)(thf)]; *S* = 0.

54

Coordinates from ORCA-job Zn

C	0.430403	4.994743	14.676921
C	0.413223	6.435573	14.734920
C	0.374437	7.280401	13.604855
H	0.277110	8.353024	13.809127

C	0.300755	7.754852	11.279427
C	0.399132	7.221412	9.990510
C	0.736527	5.244449	8.713099
H	0.729124	5.839969	7.790257
C	0.877336	3.854388	8.536423
C	0.729984	2.880269	9.589544
C	0.395690	4.148192	15.920837
C	0.609906	1.405610	9.301483
H	1.611532	0.971190	9.167184
H	0.042696	1.197394	8.387130
H	0.132676	0.927078	10.164957
C	1.099918	3.480892	7.115317
C	1.989822	1.880520	5.598282
H	1.031558	1.762220	5.077409
H	2.505000	0.919597	5.683721
H	2.613724	2.609313	5.065421
C	0.063182	9.141889	11.480438
C	0.265245	8.053854	8.845079
N	0.426579	6.882714	12.340327
N	0.608969	5.865216	9.878874
N	-0.128696	10.273048	11.703019
N	0.165092	8.692831	7.871906
O	0.488111	4.340063	13.581475
O	0.626866	3.192806	10.823349
O	0.765555	4.175565	6.156540
O	1.770221	2.313569	6.967923
Zn	0.892662	4.969571	11.714539
H	0.580461	3.106979	15.632861
H	-0.583360	4.229823	16.414053
H	1.139067	4.478271	16.658739
H	1.361239	8.726525	17.973718
O	0.840272	8.399976	15.964051
C	0.376847	7.119323	16.050715
C	0.752330	9.182745	17.183089
H	1.143645	10.168942	16.916976
O	-0.010621	6.638861	17.111720
H	-0.290803	9.257227	17.515341
C	3.642324	4.262790	12.936895
C	3.697414	6.218855	11.693519
C	3.920913	5.366251	13.966235
C	3.933568	6.680258	13.134979
H	3.129785	5.402941	14.726920
H	4.872510	5.188027	14.482126
H	3.129317	7.350550	13.466027
H	4.882534	7.223628	13.223237
H	3.070045	6.886403	11.092831
H	4.642039	6.031926	11.160887
H	4.570788	3.808445	12.560174
H	2.959654	3.478087	13.280000
O	2.996155	4.939952	11.811974

[Zn(1)(acn)]; *S* = 0.

47
Coordinates from ORCA-job Zn

C	0.283503	5.082701	14.654035
C	0.189633	6.522913	14.702133
C	0.255616	7.360839	13.567460
H	0.070979	8.426693	13.744883
C	0.426152	7.815704	11.244861
C	0.626996	7.268828	9.974127
C	1.009876	5.278512	8.739093
H	1.011955	5.865777	7.811011
C	1.145592	3.883794	8.571644
C	1.110541	2.928037	9.651298
C	0.092062	4.232363	15.881754
C	1.246030	1.443984	9.422882
H	2.071052	1.207781	8.739072
H	0.331938	1.041389	8.964181
H	1.408208	0.962217	10.393579
C	1.315733	3.511543	7.143536
C	1.085719	1.844945	5.463335
H	0.505550	2.485774	4.788069
H	0.721143	0.814526	5.424751
H	2.148821	1.890933	5.195995
C	0.148381	9.201493	11.405109
C	0.560480	8.086642	8.812187
N	0.484029	6.960650	12.324214
N	0.861141	5.913357	9.892922
N	-0.081629	10.331674	11.594222
N	0.519218	8.713651	7.826793
O	0.536558	4.434196	13.584590
O	0.986306	3.268603	10.875621
O	1.763471	4.277081	6.288853
O	0.893467	2.260803	6.841743
Zn	1.138225	5.076158	11.767198
H	0.337076	3.195774	15.623250
H	-0.946728	4.294023	16.234951
H	0.720677	4.576730	16.713189
H	0.485766	8.901928	18.008431
O	0.279557	8.522193	15.951374
C	-0.076369	7.202833	15.991455
C	-0.047640	9.295524	17.134244
H	0.283341	10.313799	16.909225
O	-0.557109	6.695382	17.001227
H	-1.129136	9.272846	17.319905
N	3.189436	5.176280	12.028350
C	4.339963	5.307166	12.081638
C	5.770810	5.468871	12.151519
H	6.010279	6.409517	12.666641
H	6.188146	5.494193	11.135634
H	6.210367	4.627726	12.705315

[Zn(3)]; *S* = 0.

39
Coordinates from ORCA-job Zn

C	-0.648792	7.210030	15.980405
C	-0.270990	6.544519	14.730314
C	0.037664	7.365395	13.619592
H	-0.003794	8.449718	13.761973
C	0.603181	7.807828	11.354965
C	0.860952	7.248318	10.105057

C	1.028953	5.233786	8.858145
H	1.180162	5.860808	7.974304
C	1.028094	3.830243	8.678174
C	1.186241	3.320228	7.313754
C	-0.291251	8.738374	16.171321
C	1.726861	4.292218	6.189679
F	2.846672	4.968835	6.576100
F	0.788222	5.220320	5.831938
F	2.041928	3.592541	5.084468
C	0.858679	2.877975	9.766149
C	0.613726	9.217312	11.543619
C	1.142074	8.067892	8.977482
N	0.340376	6.943145	12.401226
N	0.839856	5.869502	10.004480
N	0.609964	10.366115	11.752653
N	1.379041	8.692091	8.019516
O	-1.216435	6.688255	16.934387
O	0.947150	2.177233	6.937654
O	0.637854	3.231771	10.970623
C	0.967946	1.397584	9.559977
Zn	0.434729	5.004065	11.799375
F	-0.477174	9.102675	17.453347
F	-1.084125	9.542225	15.399975
F	1.005830	9.012363	15.852220
C	-0.544653	4.249807	15.895472
C	-0.265549	5.089937	14.686372
O	0.004677	4.428379	13.630537
H	0.000785	4.606796	16.777842
H	-0.271552	3.214518	15.663010
H	-1.613006	4.301668	16.151125
H	1.039705	0.919472	10.542933
H	1.829721	1.131252	8.934323
H	0.076489	1.023868	9.034632

[Zn(3)py]; *S* = 0.

50

Coordinates from ORCA-job Zn

C	-0.182384	7.167620	16.044994
C	-0.063153	6.510243	14.747888
C	0.072132	7.329747	13.596517
H	-0.067285	8.408285	13.727279
C	0.300627	7.750061	11.278797
C	0.540626	7.197000	10.021815
C	1.010867	5.215522	8.812812
H	1.104976	5.835573	7.914553
C	1.116628	3.808056	8.652512
C	1.580476	3.294393	7.369829
C	0.233709	8.690067	16.153162
C	2.274843	4.306102	6.372878
F	3.238373	5.057343	6.984417
F	1.375155	5.170652	5.812085
F	2.868946	3.640256	5.364933
C	0.706967	2.870465	9.697431
C	0.053456	9.144676	11.423769
C	0.545901	8.012373	8.854840
N	0.297324	6.900936	12.366956
N	0.753354	5.836083	9.949401
N	-0.152077	10.280436	11.601581
N	0.563970	8.629408	7.863442
O	-0.523761	6.650728	17.107293
O	1.554687	2.125798	6.985981
O	0.541277	3.222022	10.903335
C	0.438811	1.425532	9.396870
Zn	0.915136	4.986216	11.843847
F	0.326191	9.058874	17.444826
F	-0.683980	9.517054	15.563264
F	1.443129	8.938990	15.571051
C	-0.613767	4.227937	15.838467
C	-0.160239	5.054090	14.671787
O	0.125632	4.405406	13.621142
N	2.934632	4.964077	12.182793
C	3.784657	4.512403	11.234206
C	3.439374	5.465951	13.331143
H	2.716799	5.819526	14.067232
C	4.808400	5.532369	13.570258
H	5.174738	5.944133	14.509775
H	3.332880	4.118484	10.323268
C	5.165119	4.545325	11.401548
H	5.815003	4.169900	10.612102
C	5.687054	5.065533	12.589119
H	6.765331	5.106338	12.748003
H	0.090903	4.326727	16.677259
H	-0.670956	3.182374	15.515908
H	-1.584910	4.570649	16.218308
H	0.020169	0.959660	10.296249
H	1.371404	0.915707	9.113155
H	-0.242066	1.310440	8.543475

[Zn(3)py]; *S* = 1.

50

Coordinates from ORCA-job Zn

C	-0.114038	7.131407	16.043784
C	-0.069075	6.506367	14.721180
C	0.051357	7.357894	13.593570
H	-0.063831	8.434190	13.754672
C	0.227010	7.796997	11.277179
C	0.480884	7.206046	9.943092
C	0.972185	5.217794	8.762625
H	1.067793	5.821227	7.855240
C	1.089964	3.810454	8.635018
C	1.551071	3.278847	7.351773
C	0.472727	8.588073	16.193172
C	2.327001	4.254224	6.383582
F	3.288008	4.973048	7.032229
F	1.484695	5.146498	5.776338
F	2.938514	3.557725	5.406523
C	0.718680	2.872416	9.697518
C	-0.010960	9.177935	11.406448
C	0.486084	8.020702	8.795808
N	0.235059	6.954073	12.329872
N	0.695360	5.875271	9.895943

N	-0.214520	10.324870	11.548386
N	0.494598	8.670064	7.818361
O	-0.523183	6.622601	17.086701
O	1.446423	2.119435	6.952580
O	0.524245	3.252282	10.890804
C	0.541567	1.406925	9.440080
Zn	0.864082	5.032384	11.800681
F	0.674615	8.880055	17.493192
F	-0.384755	9.530613	15.694282
F	1.667245	8.741358	15.552777
C	-0.645428	4.213045	15.785140
C	-0.210097	5.051953	14.622385
O	0.028235	4.425420	13.546859
N	2.867802	5.026299	12.183059
C	3.737947	4.550448	11.265246
C	3.344473	5.541364	13.337713
H	2.606159	5.915304	14.047355
C	4.706612	5.596402	13.613925
H	5.050563	6.018224	14.557403
H	3.307890	4.146577	10.348206
C	5.113230	4.571775	11.471550
H	5.780821	4.177113	10.706811
C	5.607091	5.104468	12.665516
H	6.680699	5.135560	12.854717
H	0.054428	4.315042	16.627317
H	-0.691705	3.169036	15.456008
H	-1.623043	4.540571	16.163580
H	0.221959	0.931270	10.374032
H	1.481792	0.956907	9.089966
H	-0.193156	1.228985	8.643360

[Zn(3)(py)$_2$]; *S* = 0.

61

Coordinates from ORCA-job Zn

C	-0.656965	7.384453	15.859076
C	-0.115837	6.648538	14.722879
C	0.342955	7.400818	13.608097
H	0.313147	8.491283	13.700213
C	1.167365	7.759951	11.430318
C	1.394324	7.212945	10.165868
C	1.215863	5.308919	8.787213
H	1.319403	5.995369	7.940475
C	1.047051	3.932342	8.480139
C	0.967950	3.563484	7.071422
C	-0.445280	8.951451	15.933701
C	1.469955	4.592436	5.978631
F	2.694685	5.121063	6.270242
F	0.597105	5.635900	5.823966
F	1.577122	3.985176	4.780260
C	0.944284	2.882252	9.497261
C	1.361429	9.154685	11.650832
C	1.815253	8.047698	9.090099
N	0.756938	6.923687	12.447833
N	1.209305	5.857279	9.989027
N	1.499498	10.289065	11.893497
N	2.157760	8.679000	8.168722
O	-1.268605	6.910790	16.817324
O	0.560737	2.495447	6.614330
O	1.092284	3.119933	10.726795
C	0.707955	1.442692	9.131872
Zn	0.885760	4.886751	11.850863
F	-0.872276	9.423273	17.121147
F	-1.145738	9.616900	14.964363
F	0.868192	9.306583	15.808648
C	-0.658893	4.412051	15.927477
C	-0.074111	5.183706	14.777636
O	0.485340	4.496025	13.881312
N	2.991618	4.820158	12.280371
C	3.869779	4.435847	11.331600
C	3.470140	5.324433	13.435941
H	2.726958	5.623279	14.174258
C	4.833910	5.458398	13.686307
H	5.172407	5.871850	14.635924
H	3.441172	4.034847	10.413718
C	5.248453	4.536290	11.502560
H	5.917957	4.212361	10.706261
C	5.741168	5.059115	12.701468
H	6.815462	5.154460	12.865544
H	-0.232990	4.735479	16.886813
H	-0.456489	3.348140	15.757743
H	-1.741095	4.587239	16.004338
H	0.791916	0.843808	10.045596
H	1.420780	1.091225	8.373650
H	-0.291103	1.316784	8.690828
C	-3.987402	4.390026	11.152100
H	-5.061424	4.286270	10.992031
C	-3.252362	3.353514	11.734394
H	-3.729279	2.423063	12.041444
C	-3.323764	5.561381	10.778424
C	-1.882203	3.524079	11.920221
H	-3.856146	6.395831	10.322533
C	-1.951057	5.651803	10.998668
H	-1.267796	2.743872	12.369860
N	-1.240240	4.653789	11.558499
H	-1.394927	6.548421	10.722010

[Zn(3)(py)$_2$]; *S* = 1.

61

Coordinates from ORCA-job Zn

C	-0.332450	7.349204	16.014932
C	-0.056571	6.641922	14.763066
C	0.302274	7.433246	13.642980
H	0.311322	8.518851	13.785437
C	0.892825	7.828272	11.396106
C	1.115826	7.240473	10.054758
C	1.160393	5.278375	8.752197
H	1.272412	5.923900	7.875319
C	1.087002	3.882151	8.517013
C	1.170352	3.441298	7.123495
C	0.358091	8.747597	16.252153
C	1.931944	4.353861	6.087623

F	3.099070	4.851856	6.585758
F	1.169662	5.418385	5.687974
F	2.238756	3.646315	4.981310
C	0.935208	2.859649	9.558023
C	1.005926	9.222244	11.570445
C	1.361464	8.089753	8.957236
N	0.574251	6.993673	12.407702
N	1.054683	5.897526	9.934222
N	1.096610	10.377636	11.756361
N	1.574680	8.764399	8.020651
O	-1.025855	6.944272	16.948505
O	0.726776	2.387992	6.666288
O	0.950059	3.148517	10.785849
C	0.810606	1.398130	9.226919
Zn	0.715298	4.953311	11.811393
F	0.334702	9.061505	17.563792
F	-0.286642	9.754014	15.585306
F	1.662406	8.765337	15.855370
C	-0.764664	4.417172	15.901816
C	-0.163602	5.177739	14.754572
O	0.282716	4.485366	13.799368
N	2.837314	4.943133	12.255193
C	3.727931	4.626820	11.292884
C	3.301509	5.415047	13.430087
H	2.551962	5.657268	14.182828
C	4.660091	5.587630	13.684943
H	4.986846	5.972463	14.650572
H	3.315476	4.247944	10.357982
C	5.102408	4.769260	11.466222
H	5.781760	4.500009	10.657936
C	5.578942	5.262416	12.683904
H	6.649346	5.390586	12.850429
H	-0.274347	4.664793	16.853409
H	-0.662204	3.346818	15.688902
H	-1.824601	4.678302	16.027768
H	0.909050	0.831866	10.159743
H	1.561896	1.070131	8.496525
H	-0.170207	1.193033	8.773630
C	-4.128058	4.372005	10.952226
H	-5.191997	4.238623	10.752513
C	-3.378765	3.342567	11.528627
H	-3.834685	2.388552	11.792239
C	-3.491330	5.574751	10.636118
C	-2.021738	3.550204	11.765258
H	-4.035228	6.405352	10.187003
C	-2.130145	5.702217	10.904491
H	-1.396327	2.776586	12.210305
N	-1.405970	4.710282	11.457277
H	-1.597055	6.625111	10.671687

[Zn(sal)]; *S* = 0.

35

Coordinates from ORCA-job Zn

C	0.493752	5.113818	14.718265
C	0.313934	6.556682	14.742943
C	0.307433	7.389788	13.599345
H	0.163993	8.464797	13.781318
C	0.449706	7.831313	11.272398
C	0.608119	7.273645	10.002051
C	0.903934	5.268628	8.768327
H	0.903825	5.855622	7.838499
C	1.061698	3.869866	8.625902
C	1.085254	2.920637	9.727004
C	0.287808	9.234583	11.437591
C	0.611403	8.094456	8.840723
N	0.458688	6.973520	12.351103
N	0.759079	5.906218	9.920163
N	0.151528	10.379795	11.622716
N	0.619336	8.726672	7.858614
O	0.668426	4.404422	13.634652
O	0.961268	3.240491	10.988084
Zn	0.721245	5.004537	11.780231
C	1.205058	3.385898	7.288067
C	1.255704	1.544701	9.397557
C	0.476881	4.433394	15.969993
C	0.130378	7.211958	16.000710
C	0.296583	5.109231	17.162285
C	0.118654	6.514819	17.188086
C	1.393451	1.122674	8.088294
C	1.368613	2.046364	7.014210
H	1.273573	0.832235	10.223951
H	1.524205	0.058864	7.878708
H	1.478716	1.697339	5.987489
H	1.182042	4.116569	6.476266
H	0.614823	3.350887	15.952094
H	0.289911	4.548924	18.099493
H	-0.024625	7.033764	18.135848
H	-0.005507	8.296288	15.996559

[Zn(sal)]; *S* = 1.

35

Coordinates from ORCA-job Zn

C	0.855325	5.186687	14.594851
C	0.286725	6.532036	14.672496
C	0.126279	7.414384	13.570040
H	-0.162872	8.448875	13.795399
C	0.380428	7.929782	11.251629
C	0.646962	7.360331	9.938407
C	1.086011	5.329481	8.760316
H	1.251383	5.877891	7.824131
C	1.096102	3.910173	8.689968
C	0.694506	3.014294	9.773948
C	0.229824	9.321126	11.419280
C	0.630849	8.168716	8.784025
N	0.325039	7.068920	12.298403
N	0.867154	6.022969	9.877439
N	0.105275	10.473457	11.596354
N	0.621869	8.810997	7.803205
O	1.315128	4.641886	13.495221
O	0.246433	3.417796	10.937355

Zn	0.704558	5.143280	11.717531
C	1.470994	3.333419	7.444379
C	0.756284	1.610939	9.537006
C	0.952558	4.432422	15.799533
C	-0.086255	7.032010	15.951256
C	0.567378	4.959365	17.017888
C	0.037737	6.270918	17.098868
C	1.138787	1.098211	8.311583
C	1.503961	1.964717	7.252055
H	0.463228	0.955129	10.358191
H	1.165107	0.017466	8.160789
H	1.808445	1.553765	6.289276
H	1.754043	4.005643	6.631791
H	1.369374	3.427465	15.723923
H	0.665530	4.358617	17.923546
H	-0.269123	6.678049	18.062408
H	-0.496135	8.042184	16.007049

[Zn(sal)py]; *S* = 0.

46
Coordinates from ORCA-job Zn

C	0.458920	5.084700	14.683711
C	0.328031	6.536161	14.715084
C	0.278591	7.365887	13.566283
H	0.113735	8.438200	13.751086
C	0.304804	7.789243	11.238970
C	0.435688	7.236653	9.961704
C	0.767064	5.259837	8.705019
H	0.721406	5.849665	7.776983
C	0.937693	3.861152	8.547188
C	0.941038	2.899415	9.642285
C	0.048278	9.180548	11.401539
C	0.316748	8.056823	8.803544
N	0.408026	6.944957	12.320275
N	0.654537	5.882317	9.865341
N	-0.159739	10.314216	11.592681
N	0.232290	8.681913	7.820124
O	0.589573	4.378239	13.600056
O	0.836098	3.211818	10.900251
Zn	1.045821	5.006189	11.756019
C	1.073784	3.389945	7.205218
C	1.060883	1.520249	9.288324
C	0.431428	4.408045	15.941645
C	0.200749	7.196907	15.975678
C	0.313100	5.090908	17.137092
C	0.195931	6.503146	17.165945
C	1.198322	1.112090	7.975127
C	1.207900	2.050269	6.912967
H	1.055607	0.795852	10.104789
H	1.300168	0.047508	7.752553
H	1.316271	1.713447	5.881916
H	1.067554	4.130910	6.402096
H	0.524765	3.320400	15.922316
H	0.307364	4.531706	18.075225
H	0.100294	7.028894	18.116067
H	0.101712	8.285619	15.972756
N	3.116583	5.251831	11.844607
C	3.694022	5.822318	12.923084
H	3.024709	6.131766	13.726703
C	3.898921	4.849875	10.820704
H	3.391057	4.394291	9.969699
C	5.070364	6.011278	13.015768
C	5.282516	5.004461	10.838288
C	5.879746	5.596468	11.954684
H	5.493968	6.476893	13.905161
H	5.875349	4.665854	9.989168
H	6.961162	5.732754	11.997154

[Zn(sal)py]; *S* = 1.

46
Coordinates from ORCA-job Zn

C	0.454125	5.090954	14.664969
C	0.323319	6.549161	14.709747
C	0.255912	7.399415	13.572906
H	0.088555	8.466497	13.774625
C	0.290613	7.840370	11.247832
C	0.424082	7.263678	9.916883
C	0.738848	5.273913	8.673679
H	0.691635	5.846721	7.737224
C	0.927555	3.871398	8.543163
C	0.933124	2.915603	9.652954
C	0.071333	9.224975	11.405409
C	0.343377	8.085008	8.772735
N	0.376054	7.000105	12.307624
N	0.616791	5.925648	9.829416
N	-0.109773	10.371447	11.575986
N	0.285449	8.734601	7.797603
O	0.547569	4.405020	13.567900
O	0.785745	3.249270	10.898103
Zn	1.007699	5.065631	11.726173
C	1.093447	3.370699	7.221285
C	1.099921	1.530702	9.334991
C	0.467416	4.387692	15.910550
C	0.224700	7.178186	15.982636
C	0.374635	5.045879	17.120674
C	0.250902	6.457965	17.162410
C	1.265243	1.096217	8.034857
C	1.263063	2.023426	6.961981
H	1.100123	0.829415	10.171190
H	1.398894	0.032361	7.829896
H	1.393274	1.673576	5.937501
H	1.084808	4.088149	6.397609
H	0.565420	3.301535	15.862449
H	0.395005	4.476824	18.051769
H	0.175468	6.972128	18.121044
H	0.122788	8.265890	16.009538
N	3.060748	5.257614	11.833473
C	3.638647	5.805698	12.923394
H	2.970109	6.115648	13.727315
C	3.840475	4.856588	10.807106
H	3.330814	4.420354	9.947192

C	5.016778	5.972326	13.024422
C	5.225490	4.990756	10.834786
C	5.825070	5.559214	11.962009
H	5.442174	6.420133	13.921914
H	5.817298	4.654516	9.984166
H	6.908108	5.678714	12.011788

Zn_2O_2-[Zn(sal)]$_2$; *S* = 0.

70

Coordinates from ORCA-job Zn

C	0.008227	4.898722	14.555665
C	-0.147367	6.338726	14.714307
C	-0.171277	7.275013	13.653109
H	-0.325592	8.326986	13.935104
C	-0.037646	7.904967	11.363958
C	0.144425	7.453554	10.053480
C	0.400874	5.549719	8.665357
H	0.291700	6.191463	7.778855
C	0.539495	4.155289	8.409140
C	0.759034	3.147348	9.414004
C	-0.225610	9.290007	11.637976
C	0.119482	8.360590	8.959294
N	-0.026590	6.972664	12.372915
N	0.340619	6.099632	9.860660
N	-0.379480	10.411143	11.926359
N	0.113219	9.060761	8.024000
O	0.166334	4.286625	13.416741
O	1.046011	3.432102	10.684903
Zn	0.613405	5.131057	11.691961
C	0.435205	3.751166	7.047955
C	0.735604	1.793767	9.017489
C	-0.009964	4.111844	15.744732
C	-0.276922	6.881541	16.029252
C	-0.136302	4.682769	16.996445
C	-0.272546	6.083862	17.151151
C	0.595611	1.435995	7.682138
C	0.467972	2.418548	6.681833
H	0.869623	1.037679	9.791923
H	0.603145	0.379808	7.406977
H	0.390908	2.131414	5.633749
H	0.306649	4.527186	6.290876
H	0.108621	3.033184	15.626395
H	-0.125525	4.042331	17.880773
H	-0.366558	6.521868	18.144788
H	-0.381251	7.965065	16.124717
C	4.101147	2.693693	5.519591
H	4.207929	2.239660	4.534479
C	4.094020	1.914465	6.654517
C	3.960801	4.096819	5.649613
H	4.200209	0.829574	6.578165
H	3.958135	4.722538	4.754751
C	3.947807	2.478573	7.958641
C	3.819775	4.688023	6.890314
C	3.954098	1.559309	9.034962
N	4.102001	-1.553384	10.810840
C	3.791647	3.921017	8.091946
H	4.112034	0.502914	8.772051
C	3.956640	-0.426347	11.079935
N	3.787278	1.881593	10.307478
H	3.698609	5.768266	6.989668
C	3.781130	0.964852	11.330609
O	3.626251	4.552032	9.219709
Zn	3.153374	3.740253	10.951453
C	3.594250	1.438904	12.632145
C	3.599598	0.549025	13.740559
N	3.412931	2.798174	12.802117
N	3.590283	-0.136493	14.686608
O	2.721562	5.452461	11.938225
C	3.362377	3.366888	13.988732
C	3.014830	5.757840	13.203089
C	3.235927	4.766396	14.223222
C	3.040479	7.117849	13.577733
H	3.469038	2.738229	14.884878
H	2.903659	7.861658	12.791887
C	3.349626	5.192004	15.576885
C	3.189064	7.497030	14.906259
C	3.321133	6.530327	15.921503
H	3.479878	4.428016	16.345742
H	3.184074	8.557557	15.164414
H	3.405276	6.834280	16.964242

Zn_2O_2-[Zn(1)]$_2$; *S* = 0.

82

XYZ file generated by orca_plot on BaseName=Zn

C	0.894282	5.056878	14.963404
C	0.595176	6.439203	15.070358
C	0.611476	7.360292	13.989475
H	0.368095	8.397752	14.244146
C	0.666104	7.974717	11.693083
C	0.693270	7.493088	10.380540
C	0.886064	5.535902	9.020850
H	0.818263	6.143562	8.109055
C	1.029449	4.148128	8.818236
C	0.958545	3.152419	9.861879
C	0.662344	4.067401	16.061976
C	0.844261	1.682786	9.566070
H	1.845867	1.277606	9.355977
H	0.219561	1.471403	8.691576
H	0.444389	1.186953	10.458213
C	1.182281	3.801816	7.379328
C	1.948915	2.215849	5.787039
H	0.976254	2.220997	5.280631
H	2.357650	1.202609	5.824653
H	2.639885	2.894848	5.271529
C	0.482316	9.356632	11.964827
C	0.567539	8.377939	9.272861
N	0.805074	7.053553	12.718128
N	0.835992	6.136670	10.202528
N	0.337530	10.481353	12.246346
N	0.470791	9.056748	8.327603
O	1.414434	4.549680	13.876472
O	0.939092	3.440995	11.107804

O	0.837664	4.535829	6.454857
O	1.798844	2.615896	7.176015
Zn	1.246705	5.206056	11.957255
H	1.172421	3.131404	15.811055
H	-0.418015	3.868162	16.139035
H	0.973271	4.439432	17.043378
H	0.445954	8.462553	18.571045
O	0.467071	8.320814	16.477392
C	0.177609	6.994456	16.386694
C	0.028780	8.975437	17.697300
H	0.410038	9.997444	17.626223
O	-0.355362	6.370409	17.295274
H	-1.067161	8.972296	17.753669
C	3.937716	4.623488	10.762608
C	4.240892	3.242503	10.648755
C	4.223430	2.316053	11.725188
H	4.469194	1.280245	11.466224
C	4.159309	1.688008	14.017478
C	4.130438	2.162238	15.332600
C	3.941909	4.112635	16.702901
H	4.005029	3.499526	17.611537
C	3.804272	5.499837	16.912063
C	3.883858	6.500838	15.874234
C	4.163468	5.618898	9.668144
C	4.010148	7.967528	16.179609
H	4.626851	8.167885	17.062264
H	3.010212	8.382054	16.379606
H	4.424991	8.464294	15.294797
C	3.647434	5.839877	18.352086
C	2.874780	7.419357	19.947874
H	2.181014	6.738895	20.457687
H	2.466869	8.433024	19.912951
H	3.845351	7.411215	20.458207
C	4.339775	0.307234	13.738029
C	4.250954	1.270747	16.435515
N	4.024610	2.615541	12.997379
N	3.991142	3.518092	15.517959
N	4.482218	-0.816022	13.449483
N	4.343604	0.586091	17.376963
O	3.417967	5.124112	11.852672
O	3.901858	6.220062	14.626500
O	3.988621	5.101837	19.274509
O	3.030171	7.025026	18.557838
Zn	3.585256	4.459026	13.769417
H	3.657977	6.554680	9.928928
H	3.841356	5.252819	8.687943
H	5.243358	5.815078	9.580088
H	5.923927	0.735785	7.952675
O	4.387097	1.365607	9.236614
C	4.661196	2.695090	9.330172
C	4.828290	0.719773	8.013224
H	4.459610	-0.306998	8.082928
O	5.183913	3.327602	8.421303
H	4.401898	1.230074	7.142429

Macro $(ZnO)_2$-$[Zn(1)]_2$; S = 0.

82

Coordinates from ORCA-job Zn

C	-0.156644	5.745931	14.738510
C	0.012613	7.149660	14.432057
C	0.183424	7.664798	13.133256
H	0.200002	8.757162	13.043411
C	0.339515	7.505579	10.770236
C	0.427663	6.645929	9.672397
C	0.516298	4.386341	8.967874
H	0.581331	4.734013	7.928558
C	0.480986	2.985376	9.148484
C	0.342842	2.321905	10.420886
C	-0.414542	5.266390	16.140632
C	0.364294	0.820822	10.543664
H	1.108230	0.351715	9.888843
H	-0.612304	0.404921	10.254412
H	0.564360	0.562495	11.589144
C	0.656147	2.256510	7.888515
C	0.276219	0.302432	6.589401
H	-0.142577	0.816345	5.717505
H	-0.227536	-0.650728	6.764753
H	1.354927	0.157843	6.455988
C	0.281376	8.916078	10.582116
C	0.476306	7.146242	8.344096
N	0.295167	6.941409	12.023181
N	0.462968	5.288484	9.932040
N	0.232319	10.078324	10.478400
N	0.523692	7.498912	7.230957
O	-0.095769	4.826607	13.857425
O	0.233774	2.954859	11.520385
O	1.332447	2.701382	6.930860
O	0.024850	1.086816	7.795030
Zn	0.560171	4.904505	11.965563
H	-0.357341	4.171795	16.145327
H	-1.408195	5.590062	16.480511
H	0.307074	5.690267	16.851255
H	0.874709	10.153624	17.066468
O	0.518155	9.334223	15.164105
C	-0.076222	8.158103	15.517972
C	0.383514	10.417132	16.121381
H	0.880479	11.272601	15.655049
O	-0.611416	8.003557	16.611197
H	-0.675805	10.637047	16.303410
N	4.542593	-1.836363	5.810534
N	4.034275	-1.318366	9.916480
C	4.358199	-0.798806	6.314216
C	4.016712	-0.451042	9.132972
C	4.123846	0.493150	6.866017
C	3.965286	0.657182	8.246044
H	3.535715	1.617667	14.446011
O	4.354449	1.193209	2.019684
H	4.489855	0.623197	4.286673
C	4.245611	1.583134	4.759976
N	4.044274	1.613069	6.072755
O	3.650359	2.619987	12.678682
C	3.187431	2.580041	14.062818
H	3.714014	1.425163	10.734246
N	3.759298	1.940975	8.711673
C	3.663350	2.235970	9.995999
H	2.092928	2.646047	14.090592

C	4.524436	2.317266	2.489202
C	4.193417	2.688915	3.892053
Zn	3.366662	3.245537	7.133974
H	4.483340	2.737890	-0.144356
C	3.506018	3.543199	10.511115
C	3.219626	3.648792	11.940595
C	3.836821	4.030842	4.286911
H	3.629911	3.409463	14.625710
H	3.180097	4.827182	2.374578
O	3.580630	4.358842	5.493145
C	5.394378	3.028543	0.393546
C	3.603435	4.748194	9.722343
O	3.570472	4.757233	8.449807
O	5.087197	3.324145	1.782744
H	2.885840	6.591348	10.561311
C	3.691331	5.150000	3.289138
H	6.135841	2.221844	0.332694
O	2.575121	4.587777	12.462629
C	3.843148	6.085333	10.372679
H	3.136702	5.964090	3.769241
H	4.413016	6.705285	9.669572
H	5.801819	3.959415	-0.011034
H	4.682376	5.518129	2.987872
H	4.368951	6.009284	11.329882

Macro $(ZnO)_2$-[Zn(3)]$_2$; *S* = 0.

78
Coordinates from ORCA-job Zn

C	-0.844382	7.188155	16.072386
C	-0.342732	6.599445	14.828856
C	0.082664	7.476729	13.807163
H	0.106250	8.547642	14.030580
C	0.940529	8.024008	11.666375
C	1.332412	7.544159	10.418425
C	1.532270	5.623562	9.045453
H	1.908669	6.278858	8.252096
C	1.391446	4.235941	8.747256
C	1.510979	3.828261	7.378100
C	-0.564324	8.714760	16.369957
C	2.290602	4.710031	6.345025
F	3.467922	5.167467	6.845451
F	1.543271	5.794117	5.982717
F	2.559870	4.005925	5.232903
C	1.122566	3.215144	9.761388
C	1.057404	9.409653	11.978930
C	1.857582	8.410722	9.423027
N	0.459261	7.119693	12.586001
N	1.194423	6.183605	10.186875
N	1.129558	10.533041	12.286391
N	2.289913	9.070285	8.561451
O	-1.479939	6.597791	16.940388
O	1.002702	2.789269	6.896932
O	0.789340	3.500257	10.945859
C	1.340371	1.755843	9.477814
Zn	0.250022	5.254286	11.786862
F	-0.900467	9.012042	17.638115
F	-1.295281	9.530361	15.551669
F	0.750965	9.041863	16.210060
C	-0.509726	4.248032	15.900722
C	-0.282058	5.142140	14.717855
O	0.014221	4.544917	13.639947
H	-0.028101	4.629057	16.809109
H	-0.134151	3.250042	15.649131
H	-1.585344	4.187146	16.121682
H	1.561518	1.259091	10.429181
H	2.137917	1.575773	8.748686
H	0.423351	1.311589	9.067173
F	-2.342717	2.082025	12.230490
H	-1.202305	6.534281	9.128788
O	-1.789105	5.075109	11.294659
F	0.546367	-1.724272	2.694762
C	-2.300340	4.035350	10.817942
N	-3.037164	-1.217751	9.689837
C	-2.613516	-0.564126	8.819461
C	-2.178234	3.618828	9.451663
C	-2.313487	2.228549	9.164657
C	-3.088098	3.162498	11.852557
C	-2.098206	0.295461	7.812934
F	-3.366347	3.876791	12.956160
H	-2.682674	1.577560	9.964937
C	-1.800833	-1.586500	6.276602
H	0.762140	3.629195	2.073089
C	-1.905683	4.632959	8.431864
N	-1.975191	1.660052	8.027369
C	-1.698109	-0.196020	6.572153
N	-1.861808	-2.714246	5.982947
C	-2.116816	6.094541	8.708069
Zn	-1.032372	2.580815	6.420801
O	0.704168	1.198975	1.282413
C	-0.845952	0.334397	4.425175
N	-1.224912	0.702181	5.642493
O	-1.574272	4.339353	7.248996
C	0.073880	0.609928	2.155095
C	-0.435526	1.205277	3.392048
H	-0.855145	-0.739567	4.215038
C	-0.523398	2.662399	3.484438
O	-0.832371	3.267246	4.554250
C	-0.192478	-0.921560	1.870979
H	-2.325149	6.590046	7.753276
C	-0.312869	3.545710	2.290117
F	0.144213	-1.226350	0.604757
H	-2.921560	6.280589	9.427974
F	-4.261174	2.699365	11.348046
H	-0.711796	4.538019	2.527797
F	-1.504742	-1.258382	2.035979
H	-0.781592	3.142173	1.384848

Macro $(ZnO)_2$-[Zn(1)]$_2$ · py; *S* = 0.

93
Coordinates from ORCA-job Zn

C	-0.307220	5.627209	14.695924
C	-0.147286	7.046874	14.476024

C	0.111345	7.634425	13.221152
H	0.125467	8.730401	13.193930
C	0.451777	7.608799	10.870731
C	0.611882	6.807114	9.736132
C	0.723011	4.585513	8.910927
H	0.899129	4.980860	7.902502
C	0.632316	3.183322	9.010111
C	0.400562	2.457523	10.228984
C	-0.633464	5.066557	16.054074
C	0.346471	0.952510	10.263352
H	1.061191	0.483266	9.578796
H	-0.651907	0.608937	9.953424
H	0.529755	0.622126	11.291734
C	0.855667	2.522582	7.705979
C	0.353904	0.708683	6.255789
H	0.017617	1.340355	5.424885
H	-0.242714	-0.204280	6.320702
H	1.415691	0.471593	6.128609
C	0.426965	9.026996	10.759867
C	0.769902	7.383933	8.446921
N	0.307964	6.975175	12.084708
N	0.605018	5.439535	9.918147
N	0.402266	10.194484	10.721090
N	0.909084	7.805829	7.366110
O	-0.177543	4.757441	13.772602
O	0.260378	3.025412	11.364402
O	1.599355	2.980394	6.832645
O	0.140307	1.393626	7.521464
Zn	0.573000	4.942100	11.922863
H	-0.539074	3.975519	16.005879
H	-1.657125	5.339406	16.345863
H	0.027716	5.473670	16.829961
H	0.450267	9.912587	17.327297
O	0.254983	9.197872	15.360408
C	-0.336120	7.991612	15.604387
C	0.027028	10.221717	16.363356
H	0.540757	11.110091	15.985041
O	-0.945692	7.767661	16.645952
H	-1.047232	10.414970	16.475765
N	3.976895	-2.017706	6.054480
N	3.669718	-1.282754	10.112161
C	3.974041	-0.944668	6.516312
C	3.761706	-0.443758	9.303039
C	3.968911	0.393320	7.008554
C	3.858283	0.634765	8.382219
H	3.377110	1.643000	14.512245
O	3.825125	0.774118	2.136236
H	4.071800	0.333881	4.404155
C	4.040672	1.342225	4.839772
N	4.029852	1.469462	6.160116
O	3.600761	2.651691	12.759261
C	3.067854	2.614102	14.117161
H	3.560701	1.463300	10.820242
N	3.825922	1.947348	8.810953
C	3.658879	2.268749	10.081030
H	1.974718	2.702393	14.091646
C	4.101602	1.915391	2.504828
C	3.997960	2.394450	3.906632
Zn	4.072341	3.275214	7.197959
H	3.700018	2.181316	-0.120428
C	3.578906	3.585669	10.592371
C	3.226552	3.694640	12.005855
C	3.787035	3.786915	4.242377
H	3.497378	3.431399	14.707550
H	2.759420	4.386134	2.413127
O	3.864927	4.239619	5.426052
C	4.689950	2.458042	0.263489
C	3.743288	4.791968	9.811186
O	3.801048	4.796153	8.544205
O	4.599326	2.850325	1.658920
H	2.983150	6.589189	10.729832
C	3.402882	4.804122	3.195866
H	5.380873	1.613060	0.150110
O	2.578026	4.643687	12.505234
C	3.947607	6.126425	10.484257
H	2.898365	5.634300	3.704195
H	4.474664	6.783883	9.782779
H	5.072471	3.341561	-0.255862
H	4.305782	5.191374	2.701666
H	4.508808	6.040076	11.421840
N	6.196422	3.434120	7.294983
C	6.935109	3.364521	6.168475
C	6.832416	3.519499	8.480933
H	6.202268	3.563203	9.370257
C	8.220917	3.546971	8.585163
H	8.689353	3.618153	9.566454
H	6.383100	3.287395	5.231122
C	8.327695	3.386894	6.185959
H	8.881897	3.329273	5.249580
C	8.983722	3.480842	7.416239
H	10.073477	3.500991	7.463617

Macro $(ZnO)_2$-$[Zn(1)py]_2$; *S* = 0.

104
Coordinates from ORCA-job Zn

C	-0.059857	5.512558	14.757993
C	0.116309	6.921391	14.482768
C	0.257277	7.480627	13.195258
H	0.426356	8.563621	13.161422
C	0.394570	7.428083	10.831233
C	0.516261	6.617126	9.698286
C	0.642092	4.397745	8.884037
H	0.868639	4.786605	7.883026
C	0.593382	2.991220	8.989391
C	0.420319	2.262107	10.218189
C	-0.027384	4.978698	16.167536
C	0.550061	0.760807	10.278150
H	1.268391	0.355212	9.559451
H	-0.422281	0.299524	10.046007
H	0.829372	0.481386	11.300573
C	0.867581	2.331097	7.695735
C	0.511621	0.451528	6.286418
H	0.123526	1.028961	5.438684
H	-0.003403	-0.508376	6.373062
H	1.591039	0.305968	6.165203

C	0.505280	8.842794	10.717644
C	0.737002	7.189420	8.415476
N	0.210905	6.815311	12.049388
N	0.455311	5.252078	9.876402
N	0.591356	10.007703	10.680669
N	0.920238	7.604361	7.338219
O	-0.230435	4.636843	13.853681
O	0.184950	2.820622	11.339497
O	1.573064	2.824270	6.811077
O	0.235521	1.146385	7.533369
Zn	-0.109066	4.774546	11.831139
H	0.112678	3.892403	16.116964
H	-0.978514	5.196563	16.675486
H	0.763399	5.440872	16.771125
H	1.303029	9.733700	17.209877
O	0.816217	9.031123	15.289480
C	0.158488	7.878525	15.612448
C	0.790823	10.071694	16.300225
H	1.320929	10.917322	15.852324
O	-0.338075	7.705998	16.722648
H	-0.243709	10.347853	16.539777
N	4.350183	-1.967388	6.148865
N	3.957844	-1.218432	10.192631
C	4.262183	-0.893458	6.601509
C	3.984342	-0.378201	9.380122
C	4.150979	0.443758	7.078335
C	4.004139	0.695923	8.447636
H	2.920617	1.552047	14.446615
O	4.006879	0.792602	2.197265
H	4.261045	0.375244	4.469268
C	4.168265	1.381371	4.901314
N	4.145544	1.514939	6.218572
O	3.297044	2.606299	12.748953
C	2.694654	2.554092	14.070111
H	3.523427	1.518562	10.853716
N	3.872109	2.005305	8.857850
C	3.621390	2.326940	10.118989
H	1.612140	2.716916	13.995074
C	4.195471	1.952918	2.563632
C	4.060613	2.427056	3.962557
Zn	4.085501	3.325568	7.248513
H	3.764864	2.177690	-0.059940

C	3.455271	3.633383	10.624746
C	3.020017	3.726840	12.032014
C	3.761043	3.802189	4.294875
H	3.138373	3.319786	14.718572
H	2.692064	4.325426	2.464519
O	3.818184	4.265299	5.476844
C	4.732785	2.530315	0.317967
C	3.596724	4.838471	9.837717
O	3.705511	4.834295	8.571577
O	4.619955	2.920189	1.712050
H	2.886293	6.374877	11.187372
C	3.299782	4.788908	3.249837
H	5.485344	1.739716	0.203384
O	2.411017	4.674098	12.536097
C	3.715310	6.186184	10.499309
H	2.731345	5.575301	3.761083
H	3.770803	6.957999	9.724140
H	5.044451	3.438044	-0.206876
H	4.169822	5.248901	2.759088
H	4.635598	6.214088	11.104938
N	6.195873	3.592128	7.362792
C	6.950601	3.578366	6.245214
C	6.810194	3.698245	8.558530
H	6.165557	3.694144	9.438675
C	8.193571	3.802830	8.681146
H	8.645826	3.888068	9.668916
H	6.414073	3.482757	5.300323
C	8.339498	3.678682	6.281551
H	8.908522	3.664376	5.352423
C	8.973447	3.794333	7.521600
H	10.059548	3.875929	7.583649
C	-2.744599	4.208647	10.426585
C	-4.106263	4.275990	10.141508
N	-2.203165	4.806872	11.507537
H	-2.056892	3.666075	9.776481
C	-3.008272	5.499052	12.339300
C	-4.939476	4.992439	11.004906
C	-4.379377	5.615272	12.123712
H	-4.500390	3.777176	9.256581
H	-2.527403	5.968258	13.198388
H	-6.009950	5.065373	10.808107
H	-4.991763	6.184763	12.822364
