# Supplementary material for: Quenched Lewis Acidity: Studies on the Medium Dependent Fluorescence of Zinc(II) Complexes
Source: Chemistry. 2021 Oct 4;27(61):15159–71. doi: 10.1002/chem.202102086 (PMC8596774; doi:10.1002/chem.202102086)
Supplement: Supplementary file 3 — Supporting Information [file CHEM-27-15159-s002.pdf]

Chemistry–A European Journal

Supporting Information

Quenched Lewis Acidity: Studies on the Medium Dependent Fluorescence of Zinc(II) Complexes

Chemistry–A European Journal

Supporting Information

Quenched Lewis Acidity: Studies on the Medium Dependent Fluorescence of Zinc(II) Complexes

Supporting Information

X-Ray crystal structure determinations

Crystallographic information: CCDC-2086160 (for **[Zn(1)(MeOH)]·MeOH**), CCDC-2086684 (for **[Zn(1)(py)]**), CCDC-2086163 (for **[Zn(1)(H_2O)]**), CCDC-2086161 (for **[Zn(2)(H_2O)]$_2$·H_2O**), and CCDC-2086162 (for **[Zn(3)(H_2O)(EtOH)]**) contain the supplementary crystallographic data for this paper. The data can be obtained free of charge from The Cambridge Crystallographic Data Centre *via* www.ccdc.cam.ac.uk/structures.

Table S1: Crystallographic data of **[Zn(1)(MeOH)]·MeOH**, **[Zn(1)(py)]**, and **[Zn(1)(H_2O)]**.

compound (unit cell)	**[Zn(1)(MeOH)] ·MeOH**	**[Zn(1)(py)]**	**[Zn(1)(H_2O)]**
CCDC	2086160	2086684	2086163
description	orange block	yellow plate	orange rhombohedron
sum formula	C_{17} H_{18} N_4 O_7 Zn · C H_4 O	C_{21} H_{19} N_5 O_6 Zn	C_{16} H_{16} N_4 O_7 Zn
M/ g·mol^{-1}	487.79	502.80	441.72
crystal system	orthorhombic	triclinic	triclinic
space group	*Pbca* *ba-c*	$P\bar{1}$	$P\bar{1}$
a/ Å	13.5885(3)	9.2831(3)	8.2597(2)
b/ Å	16.1753(4)	10.4320(4)	9.4419(3)
c/ Å	19.5542(6)	12.2241(5)	11.7529(3)
α/ °	90	83.479(3)	99.982(2)
β/ °	90	78.454(3)	90.772(2)
γ/ °	90	72.082(3)	96.782(2)
V/ Å^3	4297.98(19)	1101.87(7)	895.85(4)
Z	8	2	2
ρ/ g·cm^{-3}	1.508	1.515	1.638
μ/ mm^{-1}	1.195	1.163	1.420
crystal size/ mm	0.22 x 0.40 x 0.43	0.09 x 0.12 x 0.17	0.14 x 0.22 x 0.24
T/ K	200	170	170
λ (MoKα)/ Å	0.71073	0.71073	0.71073
θ-range/ °	2.2-28.5	2.8-28.6	3.1-28.5
reflns. collected	30326	12925	10564
indep. reflns. (R_{int})	5264 (0.050)	5250 (0.026)	4252 (0.015)
parameters	288	298	261
R1	0.0388	0.0370	0.0237
wR2	0.0870	0.1030	0.0604
GooF	1.00	1.05	1.04

Table S2: Crystallographic data of **[Zn(2)(H_2O)]$_2$·H_2O** and **[Zn(3)(H_2O)(EtOH)]**.

compound (unit cell)	**[Zn(2)(H_2O)]$_2$·H_2O**	**[Zn(3)(H_2O)(EtOH)]**
CCDC	2086161	2086162
description	orange block	red block
sum formula	2 (C_{18} H_{20} N_4 O_7 Zn) · H_2 O	C_{18} H_{16} F_6 N_4 O_6 Zn
M/ g·mol^{-1}	957.56	563.74
crystal system	triclinic	monoclinic
space group	$P\bar{1}$	$P2_1/c$
a/ Å	9.8097(7)	9.0574(2)
b/ Å	10.8009(7)	14.2024(4)
c/ Å	20.7131(13)	17.8475(4)
α/ °	87.254(5)	90
β/ °	79.764(5)	96.792(2)
γ/ °	85.457(5)	90
V/ Å^3	2151.6(3)	2279.73(10)
Z	2	4
ρ/ g·cm^{-3}	1.478	1.643
μ/ mm^{-1}	1.190	1.168
crystal size/ mm	0.09 x 0.17 x 0.17	0.13 x 0.14 x 0.16
T/ K	133	220
λ (MoKα)/ Å	0.71073	0.71073
θ-range/ °	2.1-28.6	1.8-28.4
reflns. collected	23445	16805
indep. reflns. (R_{int})	10092 (0.083)	5445 (0.095)
parameters	574	328
R1	0.0534	0.0568
wR2	0.1243	0.1918
GooF	0.90	1.10

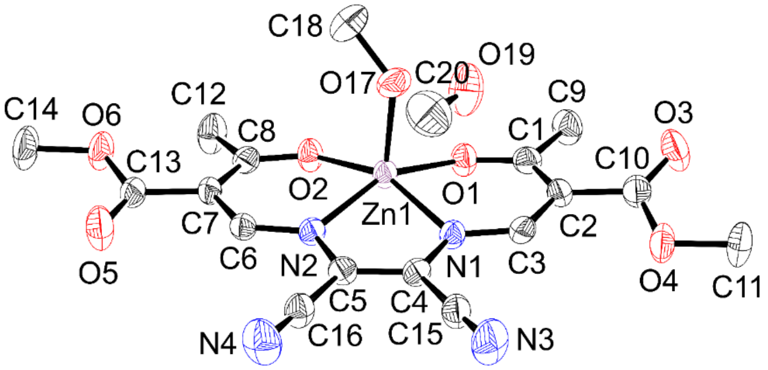

Figure S1: Thermal ellipsoid representation of the molecular structures with the applied numbering scheme of **[Zn(1)(MeOH)]·MeOH**. Hydrogen atoms are omitted for clarity. Ellipsoids are drawn at 50% probability.

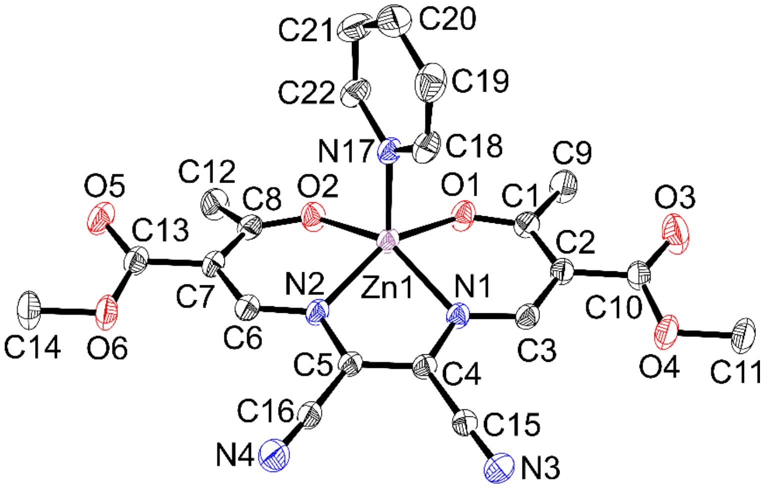

Figure S2: Thermal ellipsoid representation of the molecular structures with the applied numbering scheme of **[Zn(1)(py)]**. Hydrogen atoms are omitted for clarity. Ellipsoids are drawn at 50% probability.

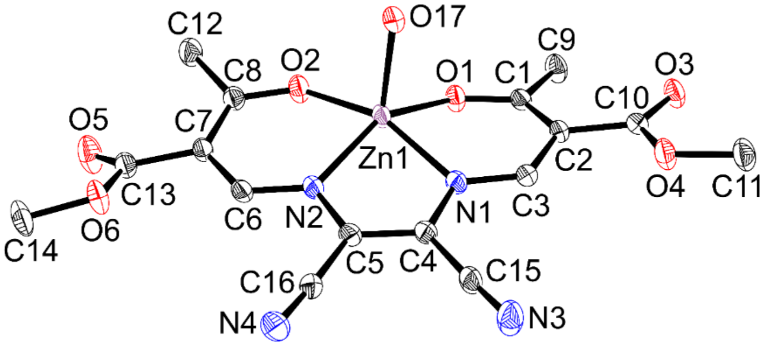

Figure S3: Thermal ellipsoid representation of the molecular structures with the applied numbering scheme of **[Zn(1)(H_2O)]**. Hydrogen atoms are omitted for clarity. Ellipsoids are drawn at 50% probability.

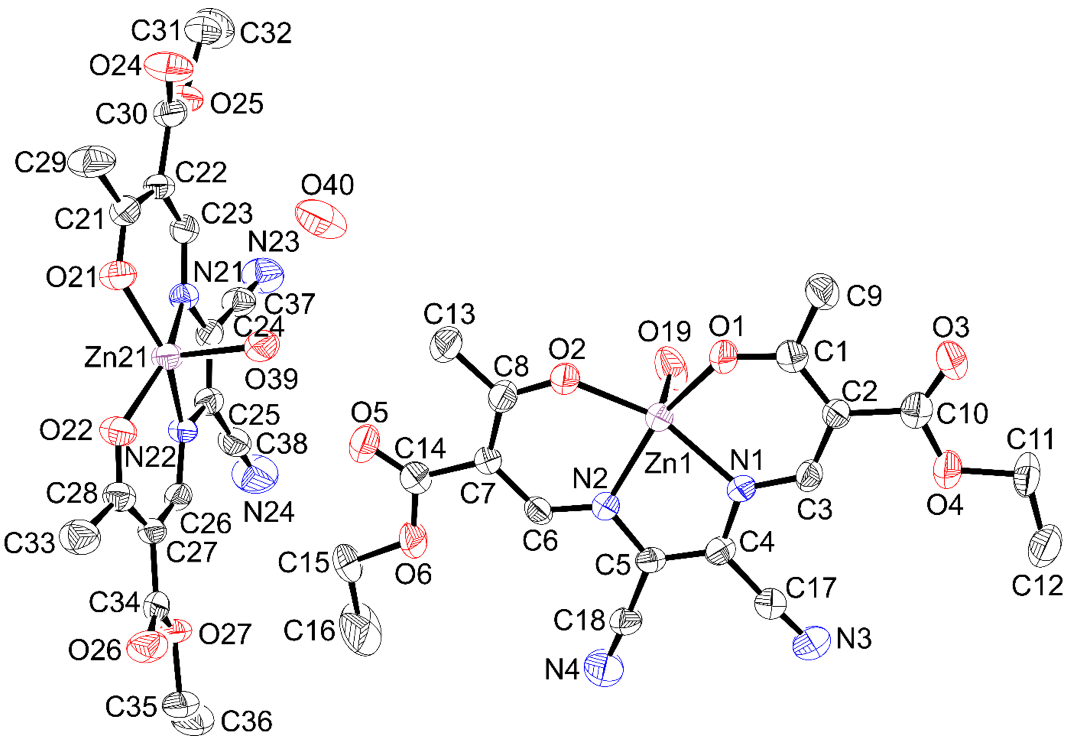

Figure S4: Thermal ellipsoid representation of the molecular structures with the applied numbering scheme of **[Zn(2)(H$_2$O)]$_2$·H$_2$O**. Hydrogen atoms are omitted for clarity. Ellipsoids are drawn at 50% probability.

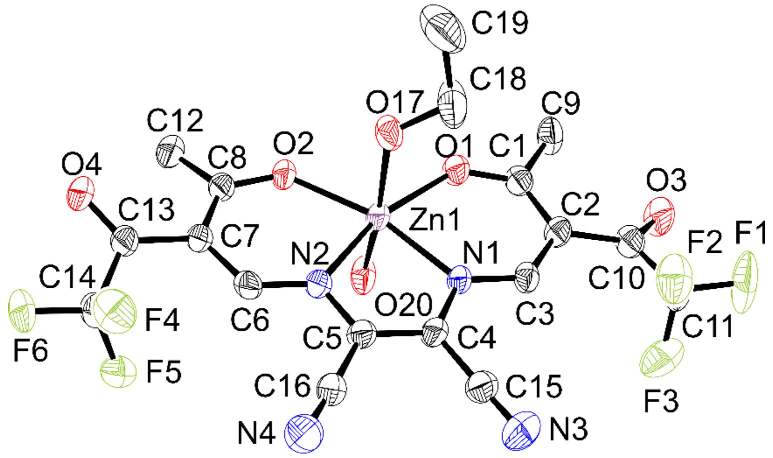

Figure S5: Thermal ellipsoid representation of the molecular structures with the applied numbering scheme of **[Zn(3)(H$_2$O)(EtOH)]**. Hydrogen atoms are omitted for clarity. Ellipsoids are drawn at 50% probability.

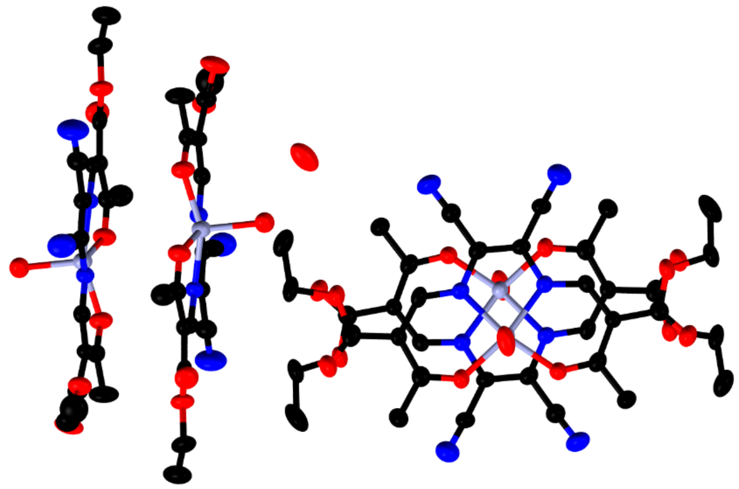

Figure S6: Side view and top view of the stacked dimers of **[Zn(2)(H_2O)]$_2$**·H_2O. Hydrogen bonds are omitted for clarity. Ellipsoids are shown at 50% probability level.

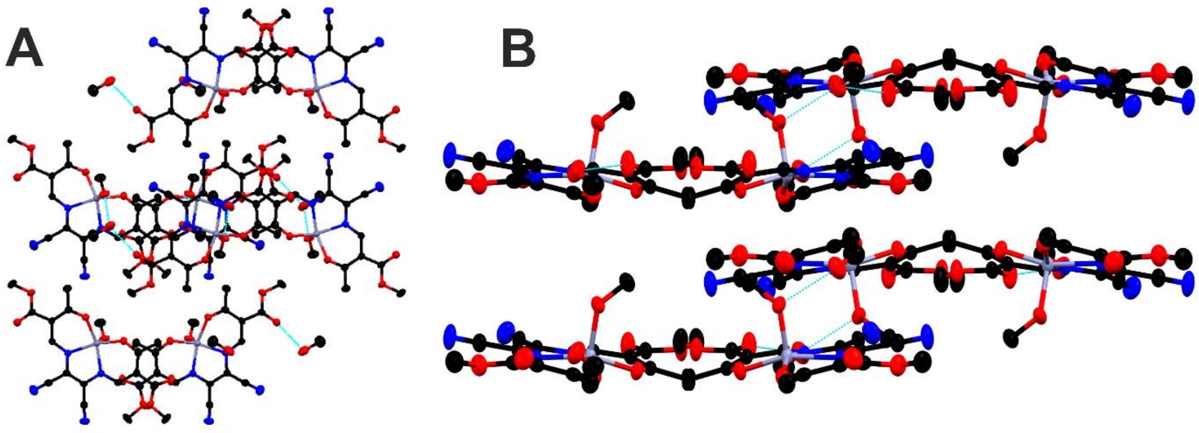

Figure S7: Molecular packing of **[Zn(1)(MeOH)]·MeOH** along [100] (A) and [010] (B). Hydrogen atoms are omitted for clarity.

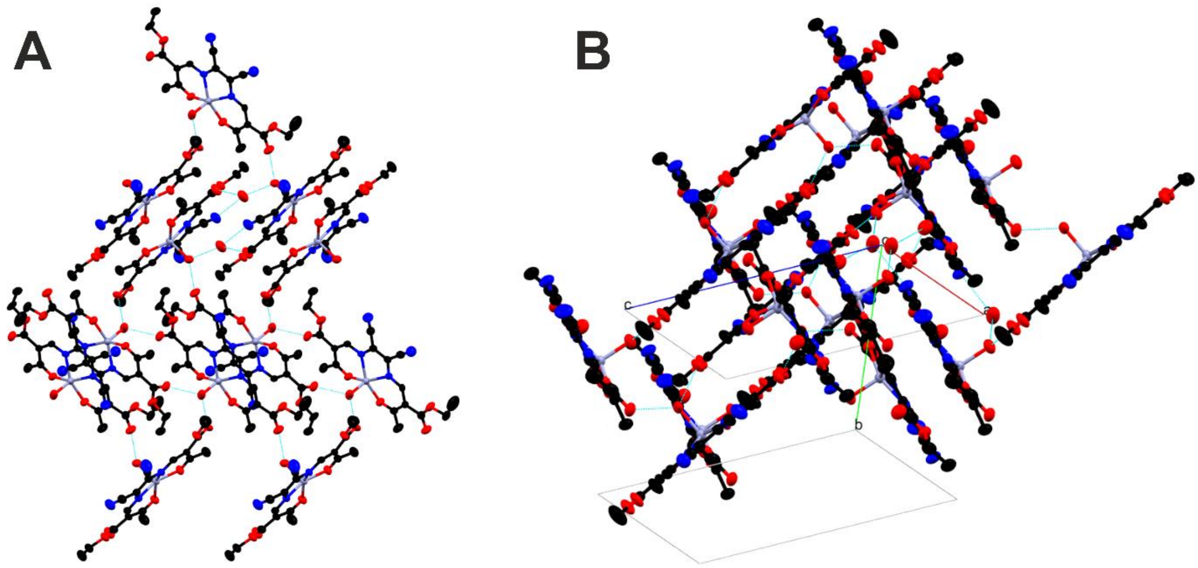

Figure S8: Molecular packing of **[Zn(2)(H_2O)]$_2$·H_2O** along [100] (A) and [???] (B). Hydrogen atoms are omitted for clarity.

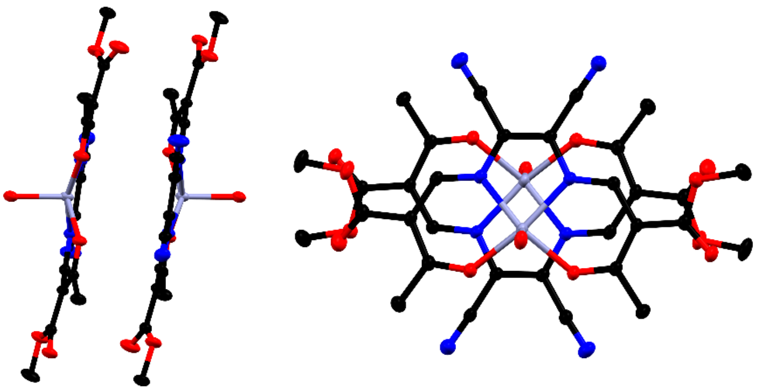

Figure S9: Side view and top view of the stacked dimers in **[Zn(1)(H_2O)]**. Hydrogen bonds are omitted for clarity. Ellipsoids are shown at 50% probability level.

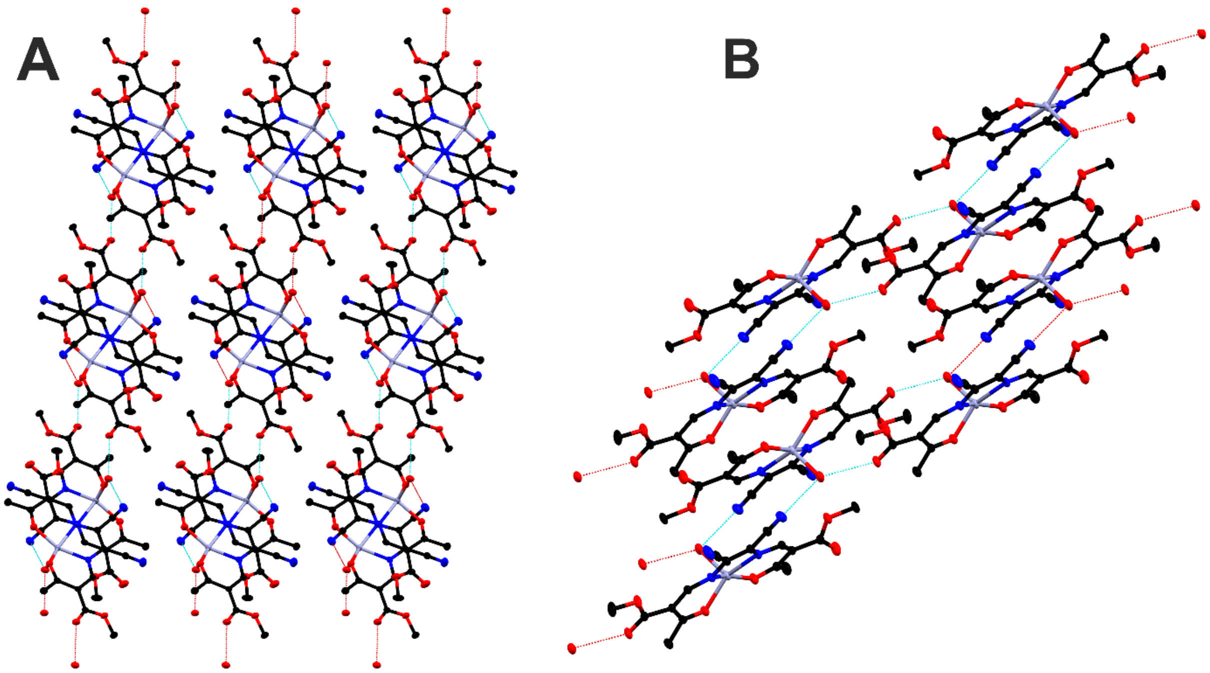

Figure S10: Molecular packing of **[Zn(1)(H_2O)]** along [100] (A) and [010] (B). Hydrogen atoms are omitted for clarity.

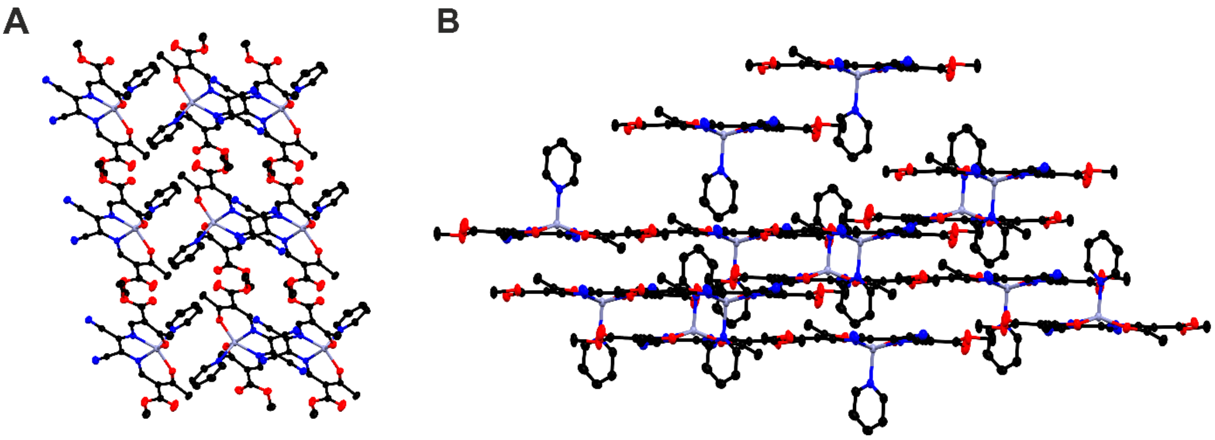

Figure S11: Molecular packing of **[Zn(1)(py)]** along [010] (A) and [???] (B). Hydrogen atoms are omitted for clarity.

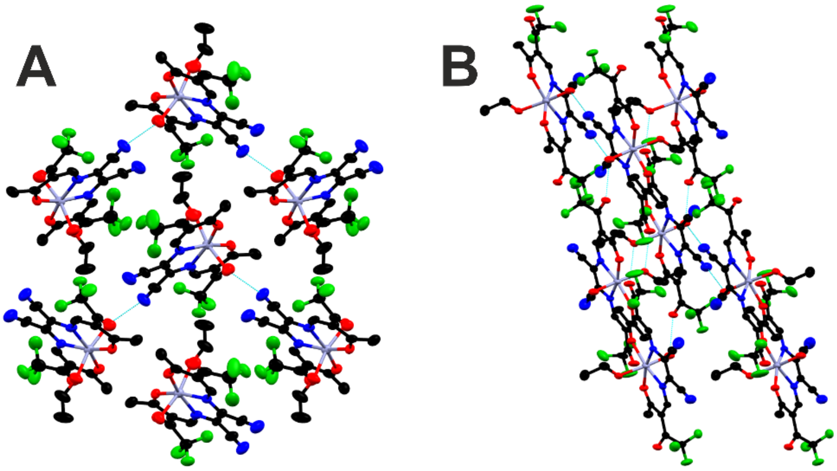

Figure S12: Molecular packing of **[Zn(3)(H_2O)(EtOH)]** along [100] (A) and [010] (B). Hydrogen atoms are omitted for clarity.

Table S3: Selected distances [Å] and angles [°] of the *π*– *π* and M– *π* interactions of **[Zn(1)(MeOH)]·MeOH**, **[Zn(1)(H_2O)]**, **[Zn(1)(py)]**, and **[Zn(2)(H_2O)]$_2$·H_2O.** Cg(I) is the centroid of the ring number I, *α* is the dihedral angle between the rings, *β* is the angle between the vector Cg(I)-Cg(J) and the normal to ring I, *γ* is the angle between the vector Cg(I)-Cg(J) and the normal to ring J.

Compound	Cg(I)	Cg(J)	Cg–Cg [Å]	*α* [°]	*β* [°]	*γ* [°]
**[Zn(1)(MeOH)] ·H_2O**	Zn1–N1–C4–C5–N2	Zn1–N1–C4–C5–N2 [a]	3.7068(12)	0.00(9)	19.1	19.1
	Zn1–N1–C4–C5–N2	Zn1 [a]	3.636	-	11.02	-
**[Zn(1)(H_2O)]**	Zn1–N1–C4–C5–N2	Zn1–O2–C8–C7– C6–N2 [b]	3.7993(7)	8.82(5)	21.7	14.9
	Zn1–N1–C4–C5–N2	Zn1 [b]	3.874	-	10.44	-
**[Zn(1)(py)]**	O3	N17–C18–C19–C20–C21–C22 [c]	3.367(3)	-	-	6.93
**[Zn(2)(H_2O)]$_2$ ·H_2O**	Zn1–N1–C4–C5–N2	Zn1–N1–C4–C5–N2 [d]	4.025(2)	0.00(17)	22.2	22.2
	Zn1–N1–C4–C5–N2	Zn1 [d]	3.801	-	3.61	-

[a] = 1-X, -Y, 1-Z; [b] = 1-X, 1-Y, 1-Z; [c] = -1+X, Y, Z; [d] = -X,-Y,1-Z

Table S4: Hydrogen bonds and angles of **[Zn(1)(MeOH)]·MeOH**, **[Zn(2)(H_2O)]$_2$·H_2O**, and **[Zn(3)(H_2O)(EtOH)]**.

Compound	Donor	Acceptor	D–H [Å]	H···A [Å]	D···A [Å]	D–H···A [°]
**[Zn(1)(MeOH)]·H_2O**	O17–H17 [a]	O19	0.76(3)	1.84(3)	2.604(3)	175(3)
	O19–H19 [b]	O5	0.82(3)	0.82(3)	2.679(3)	170(3)
	C18–H18A [c]	N3	0.98	2.54	3.296(4)	134
**[Zn(1)(H_2O)]**	O17–H17A [d]	O3	0.79(2)	2.02(2)	2.8012(16)	170.8(19)
	O17–H17B [e]	N4	0.75(3)	2.34(2)	2.9044(19)	133(3)
**[Zn(1)(py)]**	C11–H11B [f]	O4	0.98	2.54	3.470(3)	159
	C20–H20 [g]	O3	0.95	2.48	3.348(3)	152
**[Zn(2)(H_2O)]$_2$·H_2O**	O19–H19A [h]	O26	0.60(4)	2.12(4)	2.692(5)	162(5)
	O19–H19B [i]	O3	0.84(7)	1.83(6)	2.671(5)	177(10)
	O39–H39A [j]	O40	0.77(6)	1.91(6)	2.672(6)	171(5)
	O39–H39B [k]	O5	0.68(7)	2.06(6)	2.733(5)	178(10)
	O40–H40A [l]	O24	0.94(10)	2.52(10)	2.838(6)	100(7)
	O40–H40A [m]	N23	0.94(10)	2.29(10)	3.015(6)	134(8)'
	O40–H40B [l]	O24	1.04(12)	2.15(10)	2.838(6)	121(7)
**[Zn(3)(H_2O)(EtOH)]**	O17–H17 [n]	O3	0.77(8)	2.00(8)	2.761(5)	172(11)
	O20–H20A [o]	F6	0.83(7)	2.54(7)	3.092(5)	125(6)
	O20–H20A [o]	O4	0.83(7)	1.99(7)	2.792(5)	163(7)'
	O20–H20B [p]	N4	0.67(6)	2.28(6)	2.952(7)	177(8)

[a] [?] [b] ½-x, ½+y, 1-z; [c] ½-x, y, -1/2+z; [d] 1-x, 1-y, 2-z; [e] 2-x, 1-y, 1-z; [f] –x, -y, 2-z; [g] 1-x, -y, 1-z; [h] x,1+y,z; [i] -x,1-y,1-z; [j] ?; [k] ?; [l] -x,-y,-z; [m] 1-x,-y,-z; [n] 1+x,y,z; [o] -1+x,y,z; [p] 2-x,1/2+y,3/2-z.

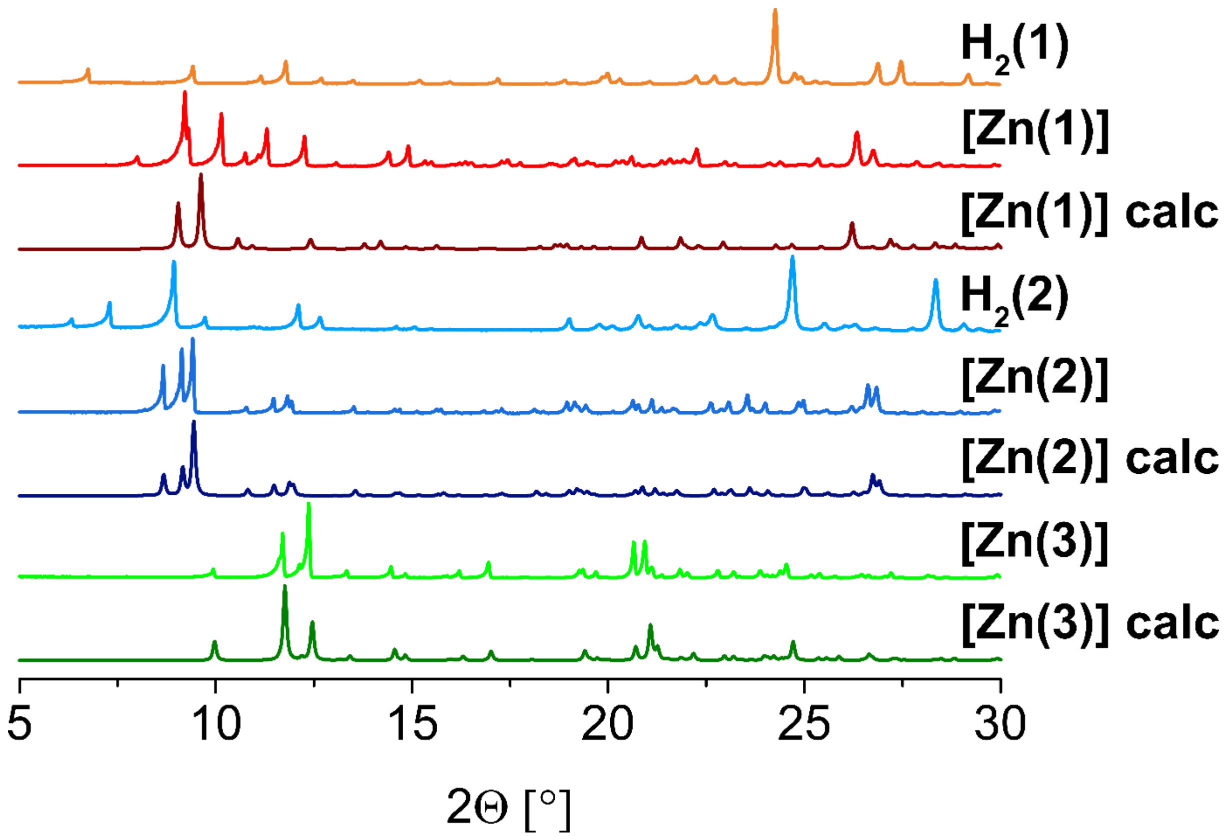

Figure S13: PXRD pattern of **[Zn(1–3)]** in the range of 5–30° 2θ at room temperature and the calculated PXRD pattern of the single crystal of **[Zn(1)(MeOH)]·MeOH**, **$[Zn(2)(H_2O)]_2 \cdot H_2O$**, and **$[Zn(3)(H_2O)(EtOH)]$** measured at 200/133/220 K.

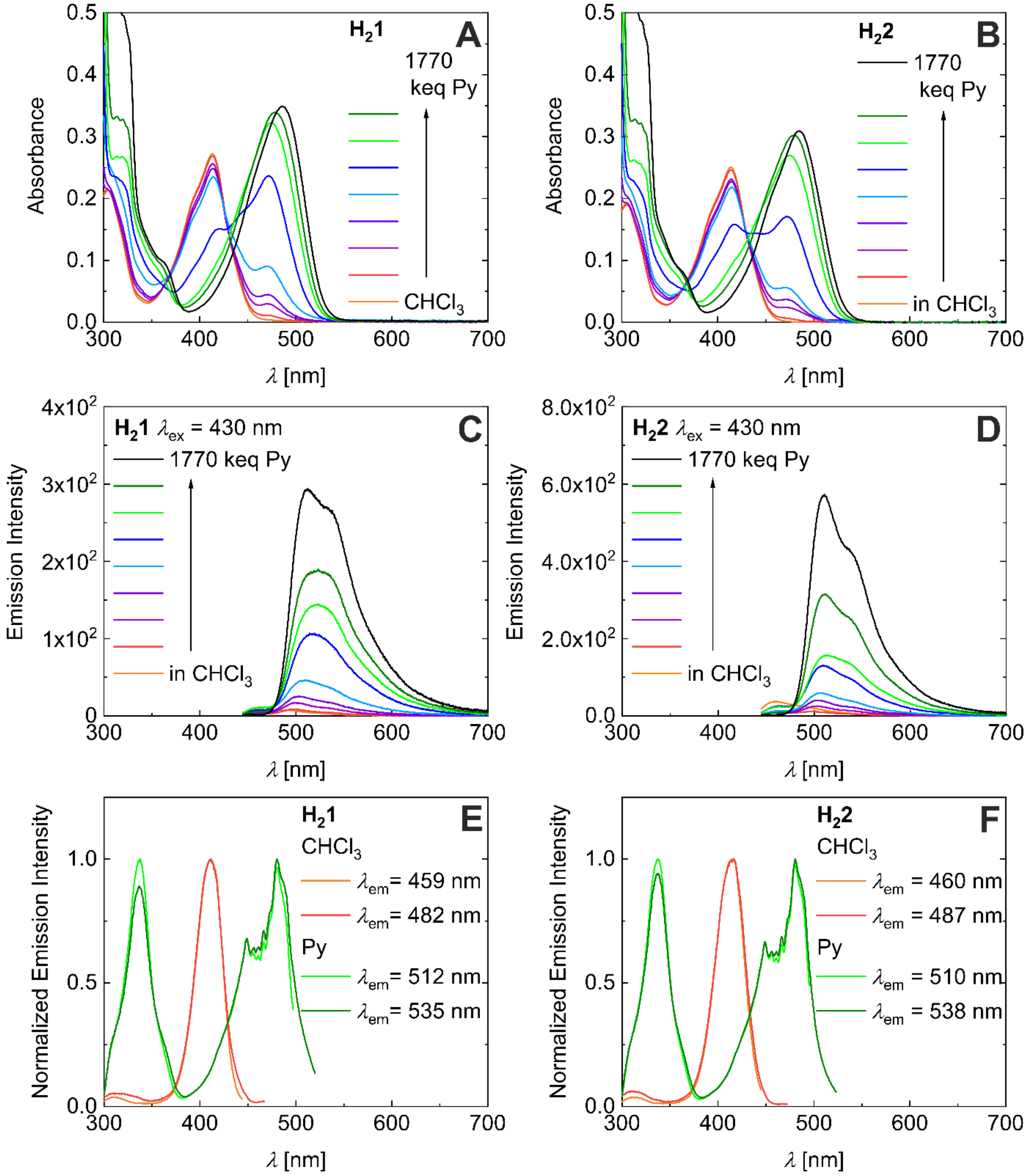

Figure S14: Absorbance (A, B), emission (C, D), and fluorescence excitation (E, F) spectra of **$H_2$1** and **$H_2$2** in various chloroform/pyridine mixtures (7 x 10^{-6} M).

Titration steps: 0 keq; 8.8 keq; 43.2 keq; 84.3 keq; 160.9 keq; 354.0 keq; 590.0 keq; 885.0 keq; 1769.9 keq.

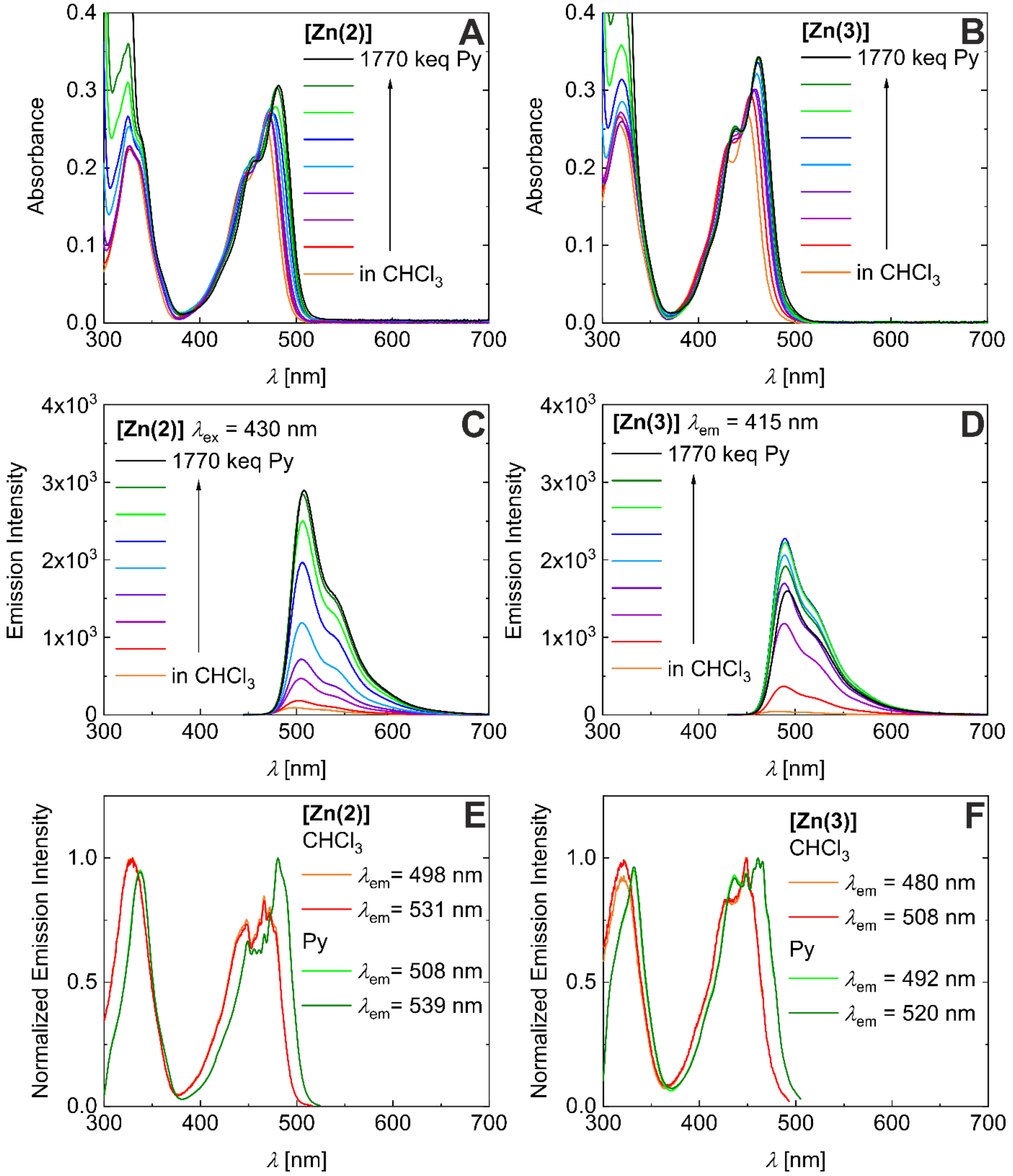

Figure S15: Absorbance (A, B), emission (C, D), and fluorescence excitation (E, F) spectra of **[Zn(2)]** and **[Zn(3)]** in various chloroform/pyridine mixtures (7 x 10^{-6} M).

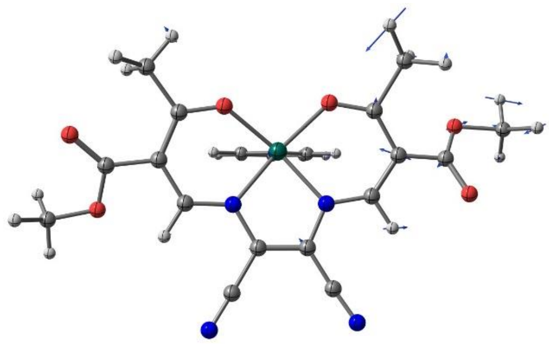

Figure S16: Visualization of harmonic vibrational modes in **[Zn(1)py]**; (left) mode at 1067 cm^{-1}; (right) mode at 1050 cm^{-1}; vectors denote dominant directions of nuclear motion (an animated gif-file of mode *1050* can be found in the ESI).

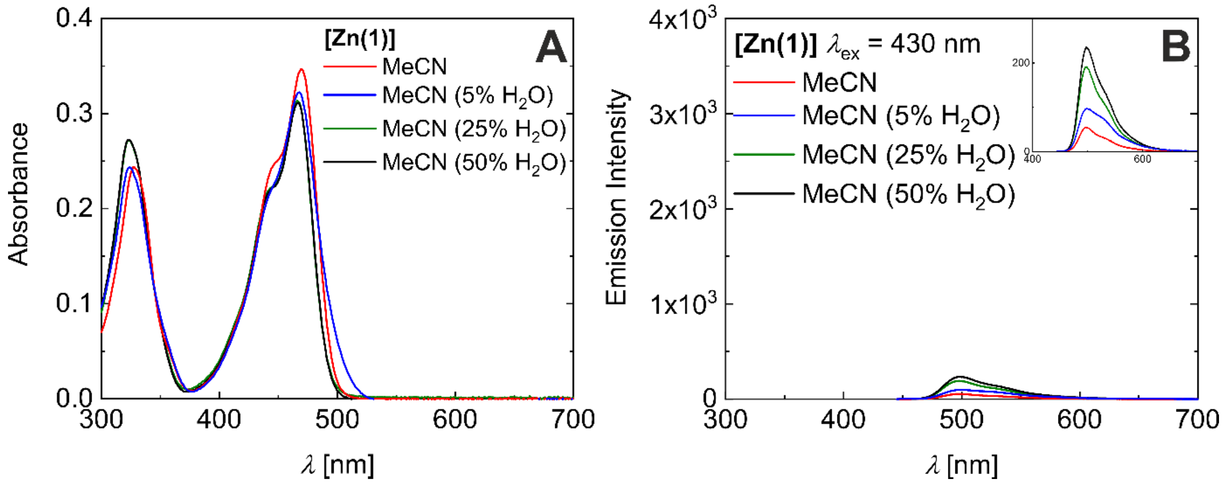

Figure S17: Absorbance (A) and emission (B) spectra of **[Zn(1)]** in MeCN with varying amount of water (7 x 10^{-6} M).

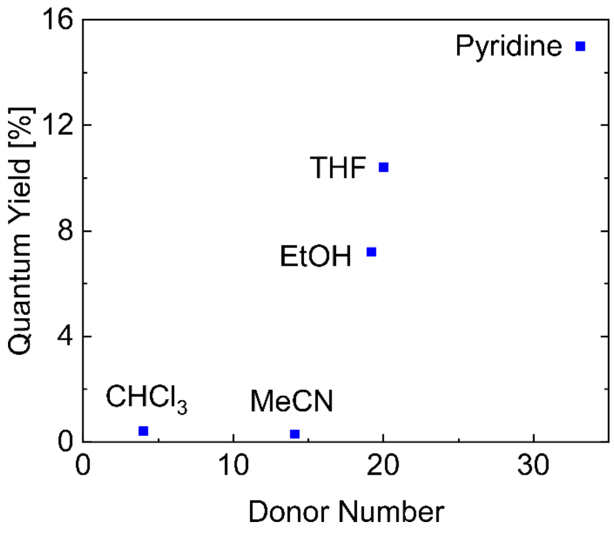

Figure S18: Plot of the quantum yield of **[Zn(1)]** solutions *vs* the donor number of the respective solvent.

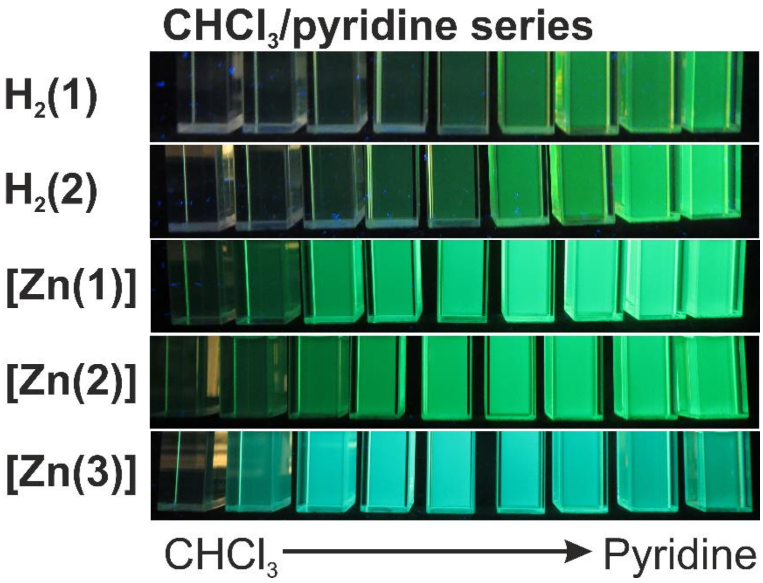

Figure S19: Photographs of the $CHCl_3$/pyridine series of the ligands and the zinc(II) complexes upon irradiation with λ_{ex} = 365 nm.

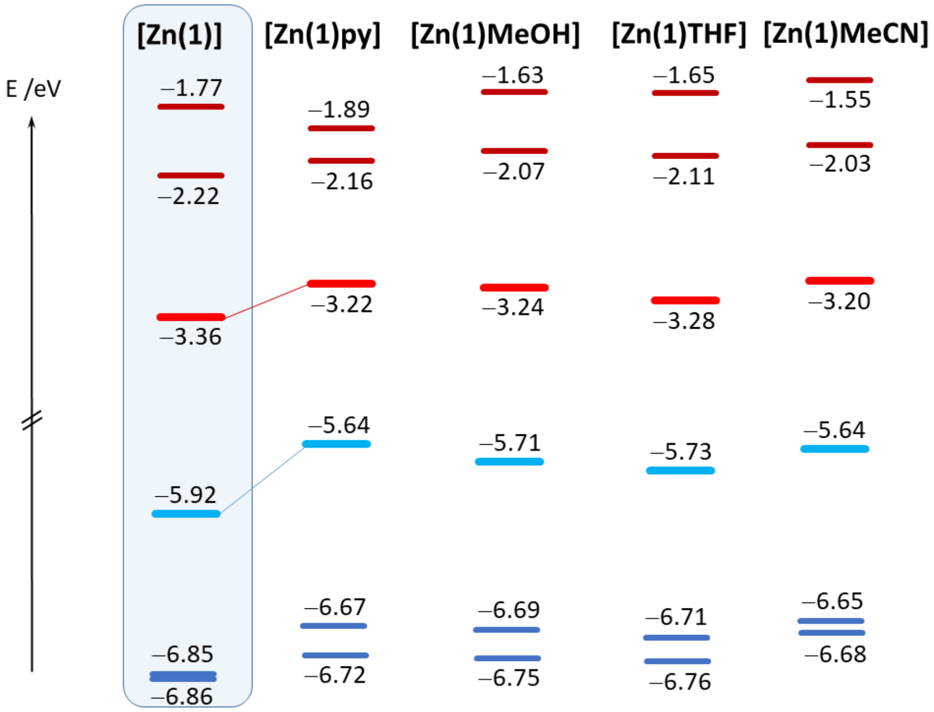

Figure S20: Frontier Kohn-Sham MO diagrams four-coordinate and five-coordinate complexes of **[Zn(1)]**; blue: occupied; red: virtual.

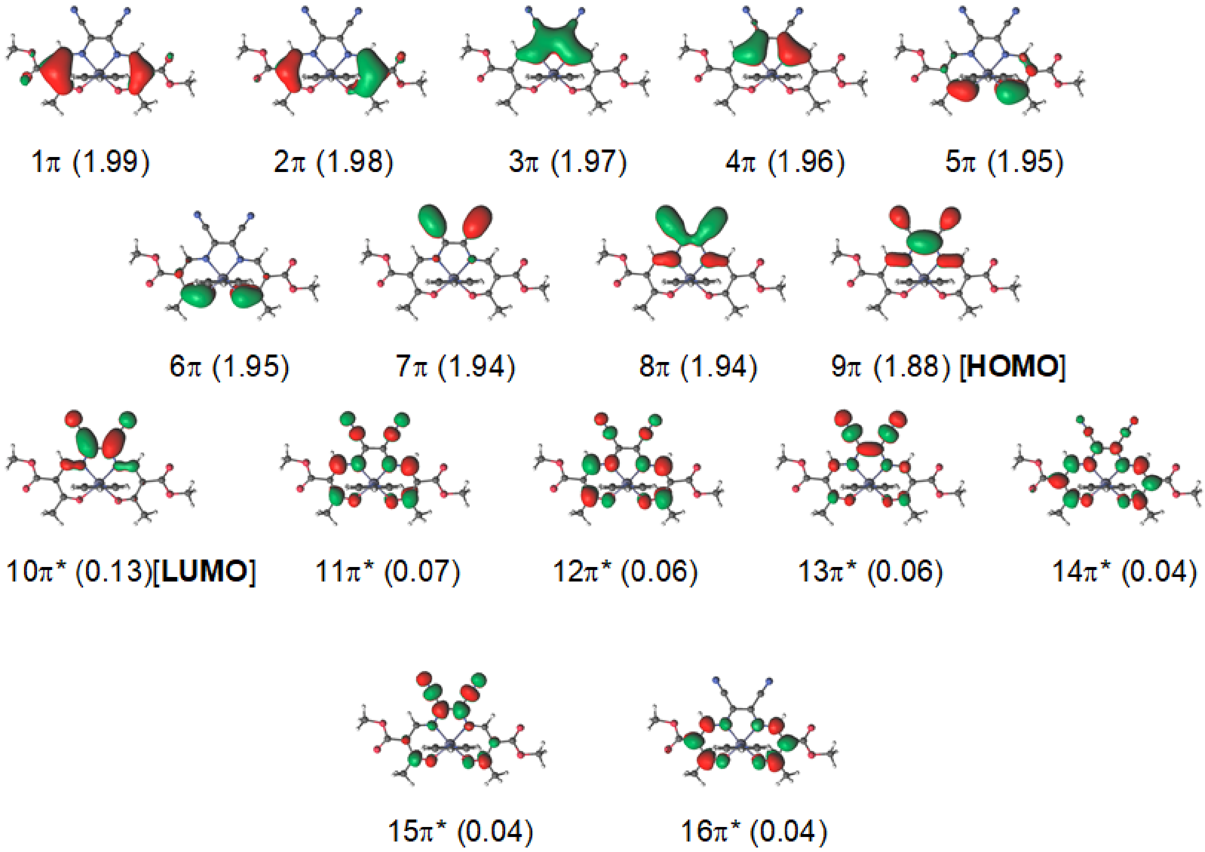

Figure S21: CASSCF(18,16) natural orbitals of the singlet ground state, S_0. In parenthesis the corresponding occupation numbers. HOMO and LUMO are marked. The HOMO markedly has a π-bonding character between the two backbone C atoms and a π-anti-bonding character with the cyano-groups (nodal plane between the nitrile groups and the C=C backbone). On the contrary, the LUMO exhibits a π-bonding character between the C atoms of the π-backbone and the nitrile groups, and an anti-bonding character between the two C atoms of the backbone. Similar features have been reported from our KS-DFT analysis. Orbitals of the S_1 and T_1 states are qualitatively very similar to the ones reported for the S_0 state. Thus, the S_1 state, as characterized by a HOMO-to-LUMO excitation experience a partial transfer of charge from the π-backbone to the nitrile group.

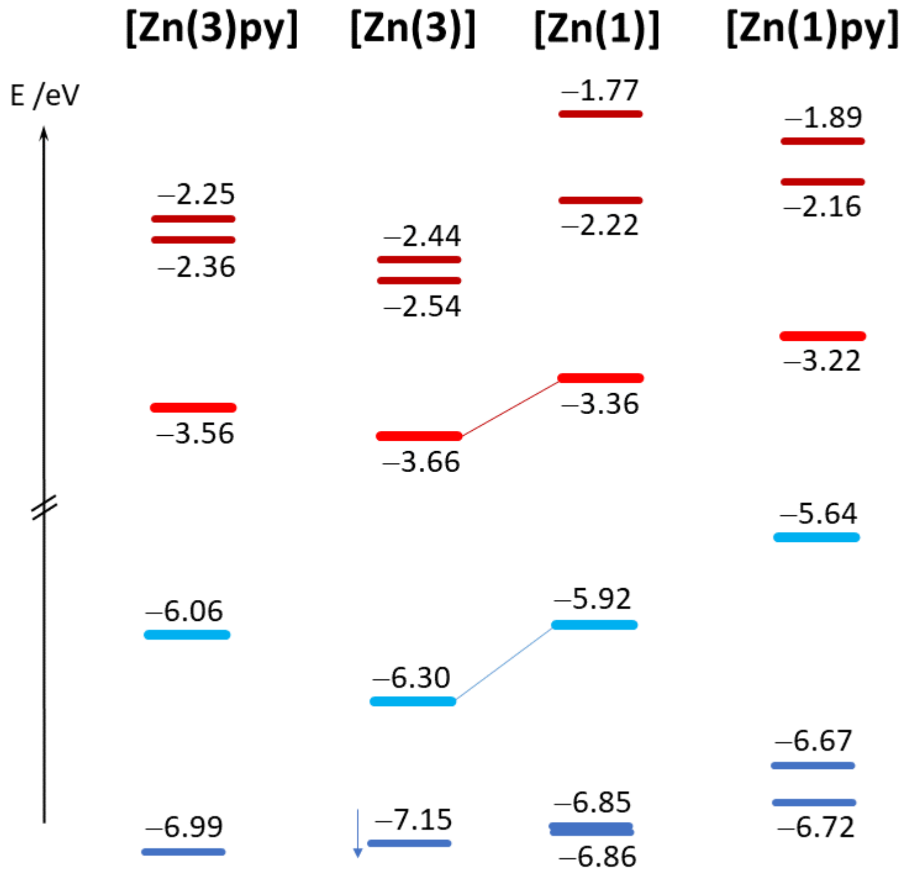

Figure S22: Frontier Kohn-Sham MO diagrams four-coordinate and five-coordinate complexes of **[Zn(3)]** and **[Zn(1)]**; blue: occupied; red: virtual.

Table S5: Selected coordination metrics in optimized structures of **[Zn(1)X]** (BP86/TZVP).

	[Zn(1)]	[Zn(1)(py)]	[Zn(1)(H_2O)]	[Zn(1)(MeCN)]	[Zn(1)(MeOH)]	[Zn(1)(THF)]
**Zn-O_{eq}**	1.970	2.029	2.010	2.020	2.011	2.011
**Zn-O_{eq}**	1.971	2.031	2.011	2.021	2.016	2.005
**Zn-N_{eq}**	2.033	2.081	2.060	2.071	2.065	2.062
**Zn-N_{eq}**	2.031	2.079	2.065	2.071	2.063	2.066
**Zn-X_{ax}**	-	2.068	2.118	2.070	2.107	2.106
**O_{eq}-Zn-O_{eq}**	97.4	95.0	95.9	95.2	96.8	96.2
**O_{eq}-Zn-X_{ax}**	-	104.0	100.9	103.1	103.8	100.6
**O_{eq}-Zn-X_{ax}**	-	104.1	105.0	103.6	101.3	103.0
**N_{eq}-Zn-X_{ax}**	-	101.8	98.3	101.4	95.8	98.8
**N_{eq}-Zn-X_{ax}**	-	102.1	96.7	100.0	98.8	98.1

Table S6: Selected coordination metrics in optimized structures of **[Zn(3)X]** and **[Zn(sal)X]** (BP86/TZVP).

	**[Zn(3)]**	**[Zn(3)(py)]**	**[Zn(sal)]**	**[Zn(sal)(py)]**
**Zn-O_{eq}**	1.967	2.030	1.948	2.001
**Zn-O_{eq}**	1.967	2.034	1.950	1.999
**Zn-N_{eq}**	2.033	2.083	2.067	2.120
**Zn-N_{eq}**	2.032	2.079	2.067	2.117
**Zn-X_{ax}**	-	2.048	-	2.087
**O_{eq}-Zn-O_{eq}**	98.7	94.9	96.4	95.1
**O_{eq}-Zn-X_{ax}**	-	105.1	-	100.3
**O_{eq}-Zn-X_{ax}**	-	103.4	-	99.9
**N_{eq}-Zn-X_{ax}**	-	103.6	-	103.2
**N_{eq}-Zn-X_{ax}**	-	104.4	-	102.9

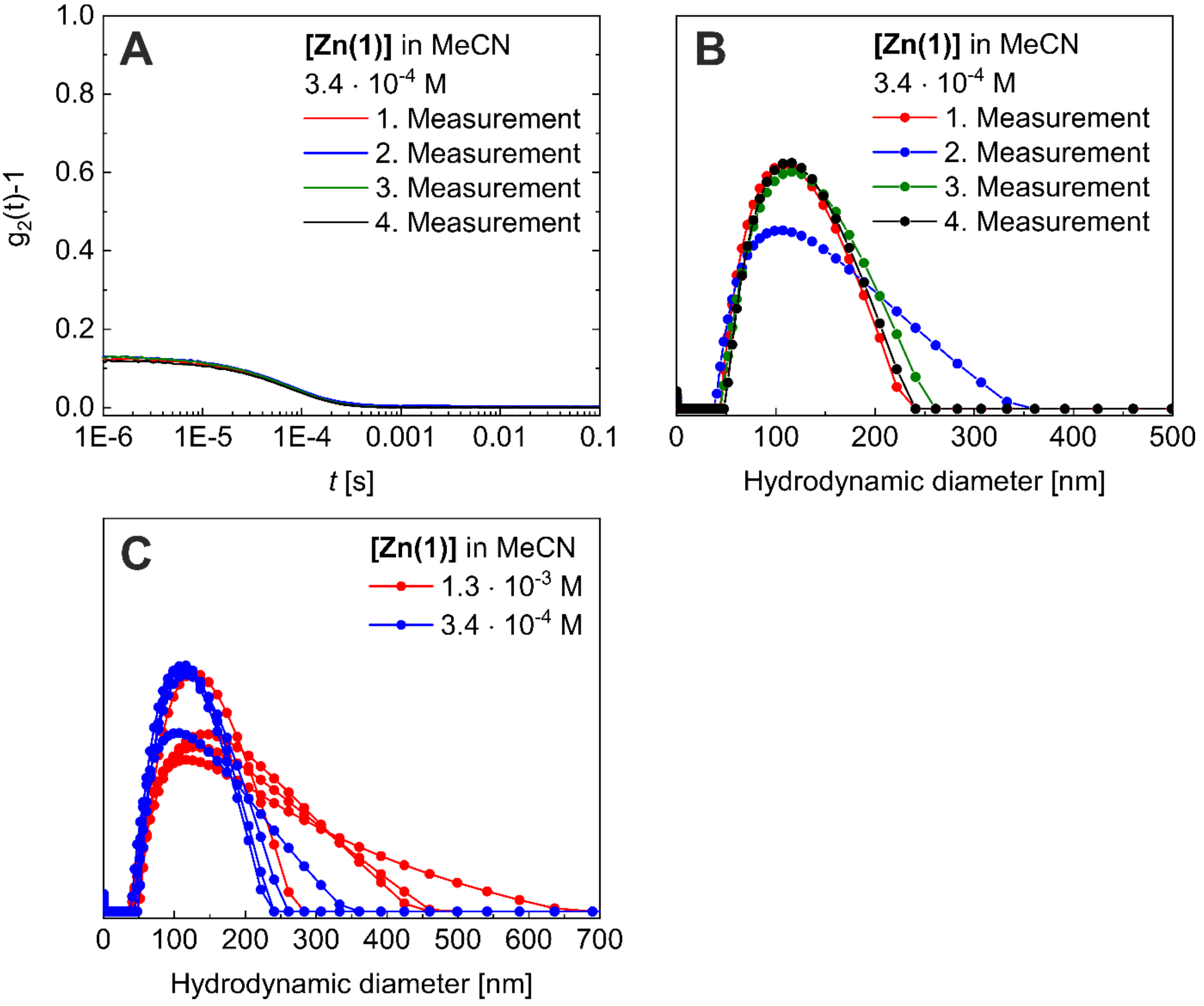

Figure S23: DLS measurements of **[Zn(1)]** in MeCN (A/B) (3.4 x 10^{-4} M): Autocorrelation function g_2(t)-1 vs t (A) and corresponding D_{hydro} distribution (B). D_{hydro} distribution of **[Zn(1)]** in MeCN at different concentrations (1.3 x 10^{-3} M: red; 3.4 x 10^{-4} M: blue) (C). All measurements were repeated 4 times and measured at 25°C. The solutions were filtered through a prefilter with pore size 1.0/0.45 μm to remove dust particles.

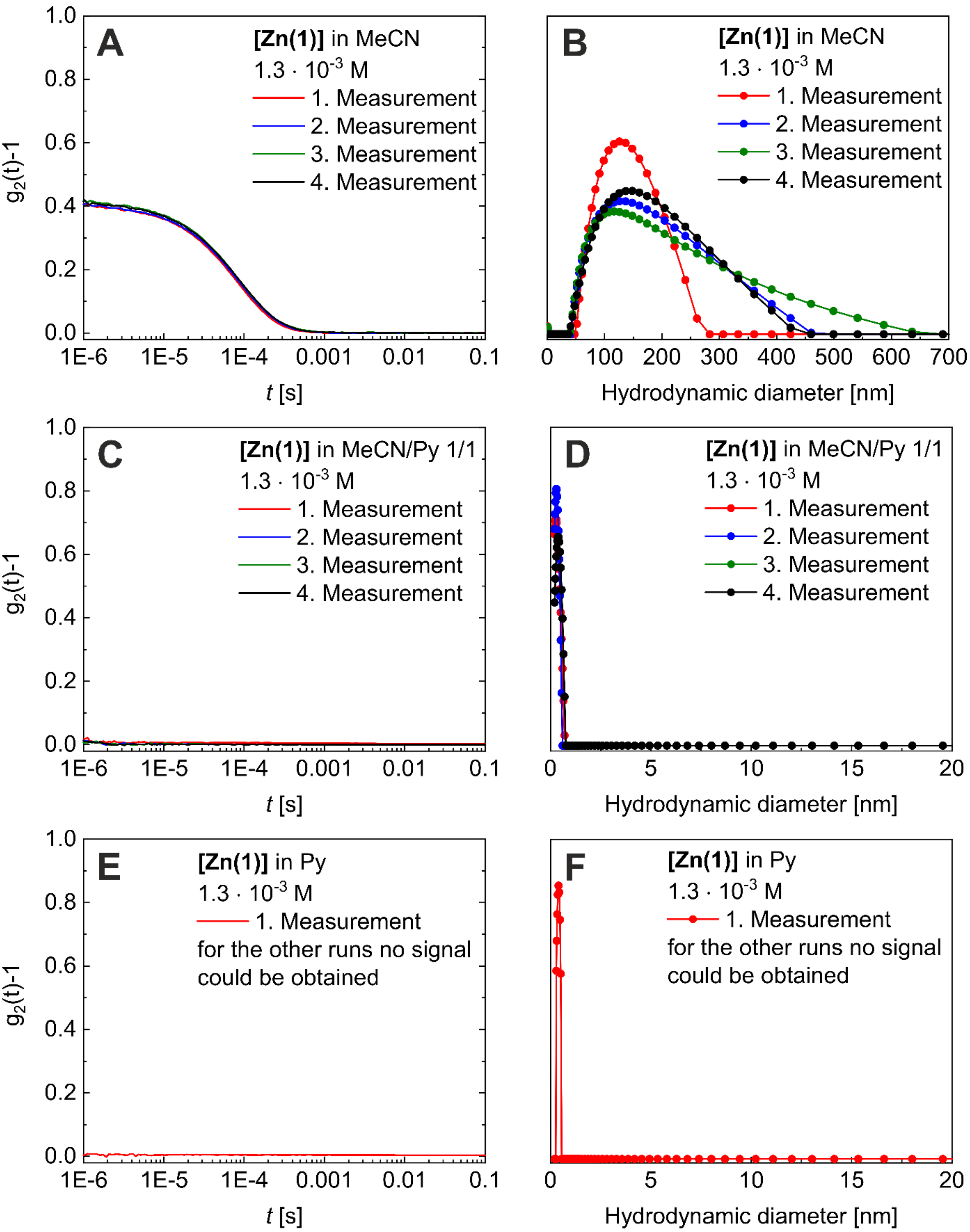

Figure S24: DLS measurements of **[Zn(1)]** in MeCN (A/B), MeCN/Py 1/1 (C/D), and pyridine (E/F) (1.3 x 10^{-3} M): Autocorrelation function $g_2(t)$-1 vs t (A/C/E) and corresponding D_{hydro} distribution (B/D/F). All measurements were repeated 4 times and measured at 25°C. The solutions were filtered through a prefilter with pore size 1.0/0.45 μm to remove dust particles.

Table S7: Selected coordination metrics in optimized dimeric structures of **[Zn(1)]** and **[Zn(sal)]** (BP86/TZVP).

	Zn_2O_2 core		macrocyle $(ZnO)_2$		
	$[Zn(sal)]_2$	$[Zn(1)]_2$	$[Zn(1)]_2$	$[Zn(1)]_2 \cdot py$	$[Zn(1)py]_2$
**Zn1-O$_{eq}$**	2.023	2.035	2.014	2.028	2.023
**Zn1-O$_{eq}$**	1.970	1.983	1.994	2.049	2.042
**Zn1-N$_{eq}$**	2.093	2.046	2.062	2.104	2.092
**Zn1-N$_{eq}$**	2.067	2.028	2.084	2.083	2.084
**Zn1-O$_{ax}$**	2.146	2.175	2.115	2.517	2.599
**Zn1-N$_{ax}$**	-	-	-	2.132	2.130
**Zn2-O$_{eq}$**	2.022	2.036	2.026	2.021	2.036
**Zn2-O$_{eq}$**	1.972	1.984	2.004	2.005	2.031
**Zn2-N$_{eq}$**	2.089	2.046	2.055	2.057	2.077
**Zn2-N$_{eq}$**	2.065	2.027	2.071	2.066	2.090
**Zn2-O$_{ax}$**	2.147	2.175	2.099	2.109	2.618
**Zn2-N$_{ax}$**	-	-	-	-	2.119
**Zn1-Zn2**	2.989	3.051	5.829	6.111	6.379

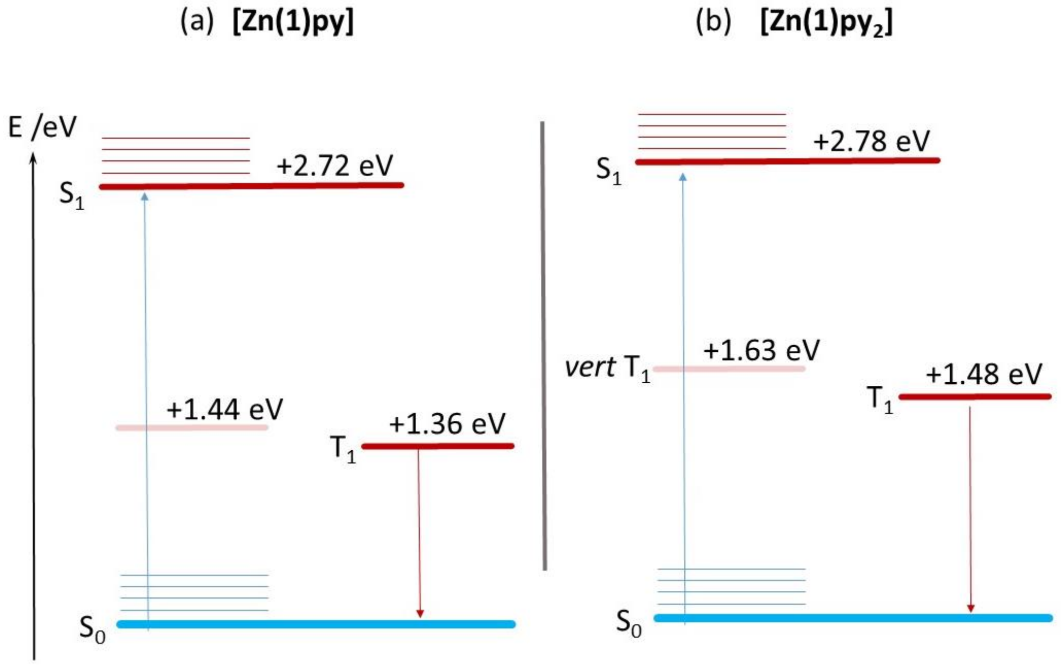

Figure S25: Jablonski diagrams of **[Zn(1)py]** (a) and **[Zn(1)py$_2$]** (b); data from KS-DFT and TD-DFT ($S_0 \rightarrow S_1$) (TPSSh/TZVP).
